# The Influence of Individual Resistance Training Variables on Muscle Strength: A Systematic Review and Meta-analysis

**DOI:** 10.1007/s40279-026-02432-5

**Published:** 2026-04-17

**Authors:** Philip Lyristakis, Daniel Wundersitz, Stephen Cousins, Minh Huynh, Emma Zadow, Brett A. Gordon

**Affiliations:** 1 Holsworth Research Initiative, La Trobe Rural Health School, La Trobe University, Flora Hill, VIC, Australia; 2 Department of Rural Allied Health, La Trobe Rural Health School, La Trobe University, Flora Hill, VIC, Australia; 3 Sport, Performance, and Nutrition Research Group, School of Allied Health, Human Services, and Sport, La Trobe University, Melbourne, VIC Australia; 4 School of Health Sciences, Sports Performance Optimisation Research Team, University of Tasmania, Launceston, TAS Australia

## Abstract

**Background:**

Resistance training (RT) is used to develop muscle strength for a variety of health and performance benefits. Because of the complexity of variable integration in a RT programme, it is unclear how manipulating RT variables influences the overall dosage (sets × repetitions × exercises × intensity × frequency × duration) expressed as a relative dosage (arbitrary units [au]) or absolute dosage (kilograms) and its effect on muscle strength development.

**Objectives:**

We aimed to investigate how RT volume, intensity and dosage influence muscle strength, and if any individual prescription variable is more important than others for developing muscle strength.

**Methods:**

Four databases were systematically searched. Only randomised controlled trials that recorded dynamic muscle strength and provided sufficient training variable data were included. Meta-regressions were performed on pooled muscle strength data, individually for the quadriceps and chest muscle groups, and RT dosage calculations. Quadratic non-linear regressions were performed to investigate if a change in volume, intensity, duration and dosage as continuous variables, as well as frequency, sex and age as categorical variables predicted the change in muscle strength.

**Results:**

There were 157 articles that contained appropriate data for analysis. A significant dose response for muscle strength for all outcomes was identified (*p* < 0.01). A plateau in muscle strength was identified at 887,000 au for chest and 773,000 au for quadriceps strength, where further increasing the dosage did not maintain or increase the rate at which muscle strength developed. Non-linear models identified volume and intensity as significant predictors of the relationship between dosage and muscle strength development for relative chest strength. Duration was a significant predictor for relative quadriceps strength.

**Conclusions:**

There is a non-linear dose–response effect for RT dosage and muscle strength, indicating there is no further benefit obtained from increasing dosage beyond 773,000–887,000 au. The variables that influence muscle strength are different between muscle groups, suggesting that the interaction between dosage and individual variables may differ between muscle groups and therefore, to optimise muscle strength development, specific training variables should be prioritised when developing RT programmes. These findings reflect relative changes in strength among primarily untrained individuals and a clear relationship with absolute strength in trained populations could not be determined.

**Supplementary Information:**

The online version contains supplementary material available at 10.1007/s40279-026-02432-5.

## Key Points

**Table:** 

Resistance training dosage is associated with an increase in muscle strength.
A resistance training dosage greater than 887,000 au does not result in further strength benefits across all relative measures.
Duration and intensity appear to be consistent predictors of muscle strength development.

## Introduction

Resistance training (RT) is a common method of physical activity that is performed across the lifespan to develop and maintain muscle strength. Muscle strength is an important outcome for improvement in quality of life, rehabilitation, functional capacity and physical performance [1, 2]. To develop muscle strength, the manipulation of training variables, such as (1) exercise selection and order, (2) rest intervals, (3) sets and repetitions, (4) intensity, (5) frequency and (6) duration, is necessary. Three ways in which training variables interact with one another have been proposed [3]. Resistance training dose (RT volume [sets × repetitions × exercises] × intensity), which considers a single RT session, RT dosing (RT dose × frequency) indicates the RT stimulus over the course of a week and RT dosage (RT dosing × duration of a programme) encompasses all RT sessions performed across a training programme. Other training variables such as the duration of eccentric and concentric phases of muscle contraction, which allows for the calculation of training density ([sets × repetitions × external load] ÷ time) [4], or the utilisation of velocity-based training data, could be included in these composite variables (dose, dosing and dosage). For the current intended purpose, including time or velocity would limit the practical application of the dose metric to quantify the total training stimulus and modify exercise programming. This is because of the difficulty in accurately adhering to cadences for each repetition and exercise, device availability and accuracy, cost and the required knowledge of specific velocity profiles to develop specific outcomes such as muscle strength [5, 6].

At present, exercise prescription guidelines have been established for both apparently healthy adults and older adult populations. For example, the American College of Sports Medicine recommends 3–4 sessions per week, composed of 1–3 sets of 8–12 repetitions at 60–70% 1-repetition maximum (RM) to develop muscle strength in healthy adults [7, 8]. Although prescription guidelines are valuable, they are broad and lack specificity. For instance, when presented with two examples, (a) a single set of 12 repetitions, across seven exercises, at 60% 1RM (RT dose = 5040 arbitrary units [au]) has a substantially different dose than (b) 3 sets of 8 repetitions, across seven exercises, at 75% 1RM (RT dose = 12,600 au). This difference is amplified further when a range is recommended for the frequency of training sessions. If example (a) was prescribed 3 days of RT, the dosing would increase to 15,120 au, whereas, if example (b) was prescribed 4 days, the dose would be 50,400 au. This difference only increases when considering the duration of a training programme, for instance, over the course of 16 weeks the dosage would increase to (a) 241,920 au and (b) 806,400 au, respectively. Given that the guidelines are broad, and that the RT dose, dosing and dosage can vary greatly, more information is needed to optimise the development of strength across the broad spectrum of guidelines established [7, 8].

Despite the body of literature examining individual training variables and their influence on muscle strength development, it is not yet clear if a single training variable is more important or how these variables in combination with one another contribute to the dose–response function of muscle strength. Although studies have attempted to ascertain a dose–response function between RT variables and muscle strength, this has not yet been attained [9–15]. Largely, this is because of the statistical approaches and methodological design of previous reviews. For instance, most of these reviews have investigated training variables as categorical values (e.g. low, moderate and high intensity/volume), which limits the data representing higher or lower training volumes, intensities and frequencies. This potentially underpowers these categories and skews results in favour of moderate categories where more data are available. Therefore, the aims of this study were to (1) investigate how variations in RT volume, intensity, frequency, duration and the composite variable RT dosage influence muscle strength, and (2) determine if any RT variable is primarily responsible for the development of muscle strength.

## Methods

This systematic review and meta-analysis is reported in accordance with the Preferred Reporting Items for Systematic Reviews and Meta-Analyses (PRISMA) protocol guidelines [16]. The review was registered prior to commencing the initial search on 1 December, 2021 with OSF (10.17605/OSF.IO/EH94V). The literature search was subsequently updated on 10 July, 2025. The protocol for this review has been published elsewhere [3].

### Search Strategy

A keyword search strategy was initially conducted across three databases; CINAHL, MEDLINE and SPORTDiscus, with Scopus included at the time of the literature search update. The PICO (populations, intervention, comparison and outcome) framework (Table 1 of the Electronic Supplementary Material [ESM]) was designed, with assistance from a research librarian, to identify relevant articles that incorporated the following constructs: healthy adults, RT, non-exercise/placebo controls and muscle strength. The search strategy across the four journal databases can be found in Table 2 of the ESM.

### Inclusion and Exclusion Criteria

Randomised controlled trials published from the earliest date possible until 10 July, 2025; investigating individuals aged 18 years and older, who were without a current diagnosed medical or musculoskeletal condition comparing a 4-week or longer RT intervention to a non-exercise control were considered eligible. Dynamic muscle strength was considered when measured by RM assessments and isokinetic dynamometer testing at velocities between 60 and 120°/second. Studies not written in English and any studies other than peer-reviewed randomised controlled trials were excluded. Concurrent exercise interventions (e.g. training for team sport and/or running) were excluded.

### Study Selection and Screening

All manuscripts underwent title, abstract and full-text screening by two independent reviewers (PL and DW, SC or EZ), with a third reviewer (BG) providing a majority decision as to whether the manuscript met the inclusion criteria. Online software (Covidence; Veritas Health Innovation, Melbourne, VIC, Australia, available at www.covidence.org) was used to screen all manuscripts and perform the assessment of methodological quality.

### Assessment of Methodological and Outcome Quality (Risk of Bias)

Risk of bias was assessed independently by two reviewers (PL and DW) using the Tool for the assEssment of Study qualiTy and reporting in EXercise (TESTEX) Checklist [17] and the Physiotherapy Evidence Database (PEDro Scale) [18], with conflicts resolved through discussion between the two reviewers [19]. The PEDro Scale consists of 11 items, one of which is not included in the overall scoring of methodological quality, meaning a maximum of 10 points can be awarded to a study. Cashin and McAuley [20] suggested that scores ≤ 3 were considered to be ‘poor’, 4–5 ‘fair’, 6–8 ‘good’ and 9–10 were considered to be ‘excellent’. The TESTEX Checklist has a maximum score of 15 points with the following classifications of study quality; “low risk” (TESTEX scale ≥ ll points), “moderate risk” (6–10 points) and “high risk” (≤ 5 points) [21]. Because of the inclusion of different studies between the chest and quadriceps outcomes, PEDro and TESTEX criteria were evaluated for each outcome separately.

Assessment of outcome quality was completed according to the Grading of Recommendations, Assessment, Development and Evaluations (GRADE) approach for systematic reviews [22]. The quality of evidence was downgraded or upgraded according to prespecified criteria. Criteria to downgrade included study limitations (risk of bias), inconsistency (substantial unexplained heterogeneity), indirectness (factors that limit generalisability), imprecision (95% confidence intervals cross minimally important difference of 5%) and publication bias (significant evidence of small-study effects). Criteria to upgrade the certainty of evidence includes a large magnitude of effect, a dose–response gradient and attenuation by plausible confounding factors [23].

Publication bias was assessed using funnel plots and Egger’s tests. Asymmetry was considered if *p* < 0.10 for Egger’s test, indicating potential publication bias [24].

### Data Extraction and Treatment

The mean and variance (standard deviation or standard error) in muscle strength for each exercise reported in eligible articles were extracted. Where standard error or other derivatives were reported, statistical approaches were used to convert to standard deviation [25]. Training variables (sets, repetitions, name of exercise, intensity [%1RM], frequency, duration) were recorded as the exact value presented in the article unless a range was specified, in which case the median was calculated because of large ranges and non-linear progression of RT variables throughout an exercise intervention [26]. Participant demographic, programme intervention, and pre- and post-intervention muscle strength data were extracted from eligible studies into Microsoft Excel (version 2510, Microsoft Corporation, Redmond, WA, USA). Resistance training dose, dosing and dosage were then calculated using the formulae previously reported [3]. Only external load-bearing exercises contributed to the dose, dosing and dosage calculations, abdominal and trunk exercises that were not performed on a weighted machine were excluded from these metrics. The RT variables were separated into relative dosage (au), which represents the integrated training stimulus without direct weight measurement by utilising %1RM to define the prescribed intensity. In contrast, absolute dosage reflects the total weight lifted by estimating the weight loads of each exercise using the %1RM prescribed and the baseline strength recorded for all applicable exercises. When applicable, relative and absolute dosages were adjusted through mid-testing strength assessments performed throughout the included studies. This provided the following RT dosage outcomes; ‘relative chest’, ‘relative quadriceps’, ‘absolute chest’ and ‘absolute quadriceps’.

Muscle strength from the chest and quadriceps was analysed as these muscle groups were most frequently reported in the available literature, and combined, these two muscle groups are an indicator of whole body functionality [27]. There are a number of exercises that can be performed to assess muscle strength changes of the quadriceps and chest musculature; when included studies used multiple exercises, only a single exercise was chosen to represent the muscle group for this study. For transparency on how this process was undertaken, a hierarchical decision (exercise) tree was developed (Table 1). In addition, where a study had more than one exercise intervention group, providing all necessary data were available to calculate dosage outcomes, these intervention groups were included in the ‘treatment group’ analysis and compared to the control group.

**Table 1:** Hierarchical decision tree of ideal exercise extracted from included studies for the chest and quadriceps musculature

Hierarchical order	Chest musculature	Total group number	Quadriceps musculature	Total group number
1	Bench press	138	Barbell squat	58
2	Chest press	84	Leg press	188
3	Incline press	0	Knee extension	122
4	Chest fly	0	Half squat	0
5	Arm cross	2	Deadlift	2
6	Pectoral exercise	2	Snatch	0
7			Clean and jerk	2
8			Isokinetic knee extension 30° > 90° > 100° > 120°	21

Exercise #1 refers to the ideal exercise to represent the chest or quadriceps musculature in a muscle strength assessment, with the following numbers indicative of the next ‘best’ option for exercise testing. Total group number represents the frequency with which an exercise was selected according to the hierarchical decision tree, and subsequently analysed

### Data Analysis

The statistical analysis differs from the initial approach specified in Lyristakis et al. [3] in that model selection via multimodal inference was used as an additional step to enhance the predictability of our results. The primary goal of model selection was to identify the most suitable model that could provide the best predictive performance for our dataset. To achieve this, we implemented permutation-based testing using the permute package in R. A total of 1000 permutations were performed for each model to construct null distributions for test statistics. This number was selected as a standard compromise between computational feasibility and statistical robustness. We explored a permutation method, which is a robust statistical technique used to evaluate the significance of our model’s performance. The permutation method involves repeatedly shuffling the data and recalculating the performance metric to create a distribution of performance scores under the null hypothesis. This allows us to estimate the variability and significance of our model’s performance, helping us to distinguish between genuine predictive power and random chance [28]. By incorporating the permutation method into our model selection process, we aimed to provide an added layer of robustness to the evaluation of model performance, supporting the distinction between models capturing a true signal versus those potentially overfitting random variation. This approach aligns with established statistical practices and theoretical advantages of permutation testing as documented in previous literature [29]. Regular meta-regression models are effective in identifying relationships between variables and providing predictions based on those relationships. However, they may not fully account for the variability and potential overfitting inherent in the data.

A meta-analysis was performed using the “metafor” [30], and “clubSandwich” packages in the R programming language [31, 32]. The included studies reported outcomes across several subgroups (from repeated measures taken on the same sample). The pooled standardised mean differences (SMDs) of the extracted muscle strength data (continuous outcome measure) were estimated for all four outcomes, using the DerSimonian and Laird random effects method [33]. For studies with more than two intervention arms, a shared-control approach was used in which the control group’s sample size was proportionally divided to maintain independence among comparisons. In addition, robust variance estimation was used to adjust for potential dependence among effect sizes drawn from the same study. Linear and non-linear dose–response relationships were estimated by fitting fixed effects and random effects models using the one-stage approach [34], to compare the SMD for strength against the individual prescribed training variables (volume, intensity, frequency and duration), demographic variables (sex and age group) and composite training prescriptions (i.e. dosage). Between-study heterogeneity was estimated with Cochran’s Q and Higgins and Thompson’s I^2^ statistics. Maximised log-likelihood and the Akaike Information Criteria were used to compare alternative models to identify the best-fitting model [35]. This analysis allows for curvilinear relationships by using transformations of the dose (e.g. splines and polynomials) and accounts for between-study heterogeneity in the true dose–response relationships by adding random effects in the regression coefficients of the transformation of the dose (individual RT variable along with calculated dosage). Random effect models also assist in controlling for unobserved heterogeneity when the heterogeneity is contact over time and not correlated with independent variables [33]. Individual study data were tabulated to identify biases and limitations with SMD as a measure of effect represented in forest plots.

Generalised additive models were systematically constructed to explore relationships for each of the four outcomes. These are designed to capture complex and non-linear relationships within data by allowing flexible modelling of each predictor’s effect while accommodating both linear and smoothed components. The initial model (Model 1) focused exclusively on dosage, interpreting it as both a fixed effect and a smoothed effect. Subsequent models investigated the effects of additional predictors (volume, intensity, frequency, duration, sex and age group) as both fixed and smoothed effects. Multicollinearity was assessed using variance inflation factors prior to inclusion of individual predictors. Where strong collinearity was observed, variables were either excluded or principal component diagnostics were used to verify that multicollinearity was not unduly influencing the model results. Fixed effects were interpreted using coefficients ‘*b*’ and standard errors ‘*SE*’, shedding light on the linear relationships between predictors and the response variable. In contrast, smoothed effects were assessed through effective degrees of freedom for the smooth effects (*e.df*) and reference degrees of freedom (*r.df*). The *e.df* serves as a critical indicator of the flexibility and complexity of the smooth effects. A higher *e.df* suggests increased flexibility, allowing the smooth effect to capture intricate non-linear patterns in the data, while a lower *e.df* indicates a smoother, potentially more linear relationship. In contrast, a higher *r.df* suggests a more flexible fit to the data, meaning that smoothing function is capturing more variability in the predictor–outcome relationship. In contrast, a lower *r.df* suggests a more constrained or linear relationship. This dual interpretation approach facilitated a comprehensive understanding of both linear and non-linear aspects of the data, enabling a balance between model complexity and interpretability. To further characterise non-linear dose–response patterns, segmented regression models were applied to the fitted dose–response curves to identify points at which the slope of the relationship between dosage and strength changed.

Model selection, using a maximum likelihood approach and correct Akaike Information Criteria [35], was then explored for different linear combinations of the moderators, which included volume, intensity, frequency, duration, dose, dosing and dosage. The effects of each moderator were estimated (along with 95% confidence and prediction intervals, where appropriate), with all other factors held constant. We considered only main effects models, resulting in 2^7^ = 128 candidate models, focusing on models that contained none, one and up to seven moderators. The variable importance (VI) scores for each predictor were determined using best subset selection, as depicted in the VI plot [36]. This approach ranks predictors based on their contribution to the predictive power of the models considered. A VI score above 0.8 was established as the threshold for determining a variable’s importance within the model. Importantly, a variable deemed “important” by this threshold does not necessarily equate to statistical significance. While statistical significance typically assesses whether the effect of a predictor is unlikely to have occurred by chance (often using *p*-values), VI scores measure the relative contribution of each predictor to the overall model performance. Therefore, a variable might have a high VI score, indicating it contributes meaningfully to the model’s accuracy, but still not achieve statistical significance if its effect size is small or if the data variability is high. Conversely, a statistically significant variable might have a lower VI score if its contribution to model performance is modest relative to other predictors. These VI scores provide a holistic view of the importance of each variable across the entire set of models considered. Given the potential for multiple models to be identified as “best” based on correct Akaike Information Criteria evaluations, we also calculated model-averaged parameter estimates. These estimates offer weighted averages of the coefficients from the various models, enhancing the robustness of our understanding of the relationships between predictors and outcomes. Unless otherwise specified, results are presented as SMDs ± 95% confidence intervals.

## Results (Included Studies, Quality Appraisal and Risk of Bias)

**Fig. 1:**
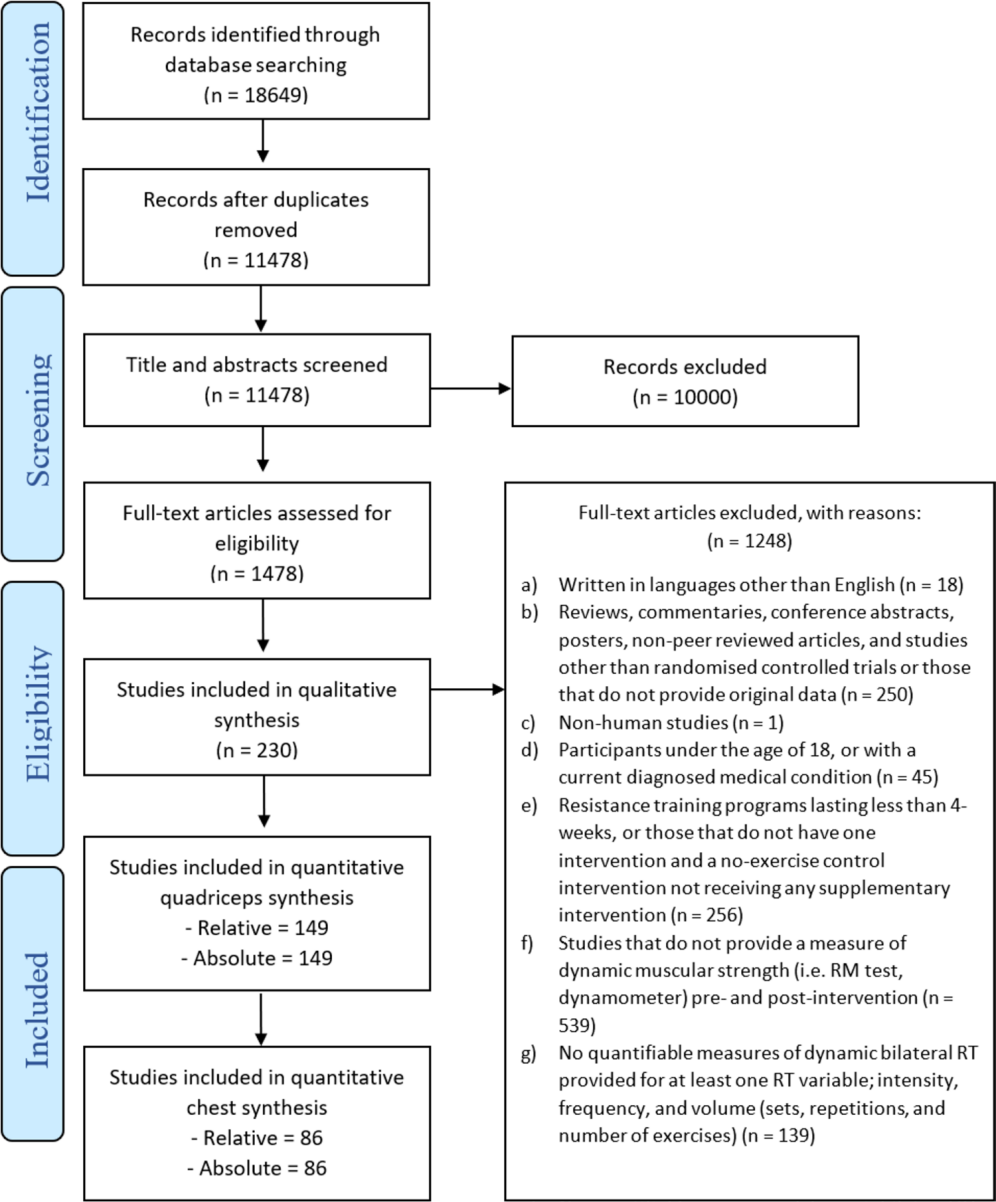
Preferred Reporting Items for Systematic Reviews and Meta-Analyses (PRISMA) flow diagram of the systematic searches, record exclusions and included studies for qualitative and quantitative syntheses (adapted from Moher et al. [55]). *RM* repetition maximum, *RT* resistance training

Of the 18,649 studies identified, a total of 230 were considered eligible for data extraction (Fig. 1). Of the 230 studies, 157 studies contained muscle strength data from the chest and quadriceps (ESM). For the chest outcomes, 86 studies were used in the analysis for both the relative and absolute outcomes. In contrast, 149 studies were used in the analysis for the relative and absolute quadriceps outcomes (Table 3 of the ESM). The final analysis comprised 89 and 79 treatment groups for the relative and absolute dosage analysis, respectively, for the chest outcome, and 165 and 139 for the quadriceps outcomes. Between the chest and quadriceps outcomes, the mean PEDro scale was 5 ± 1, whereas the TESTEX Checklist mean was 9 ± 2. In total, 28 studies were classified as having a low risk of bias according to the PEDro scale in the chest outcomes, and 62 for the quadriceps. In contrast, 13 studies were classified as having a low risk of bias according to the TESTEX Checklist in the chest outcomes, and 29 in the quadriceps. From the PEDro scale, the most common methodological weaknesses among the eligible studies were allocation concealment, blinding of assessors and the lack of intention-to-treat analyses, while the TESTEX also identified these weaknesses as well as identifying that few studies specified the method of randomisation, and often did not monitor physical activity in control groups (Tables 4–7 of the ESM).

### Effect of Resistance Training (RT) on Muscle Strength

**Fig. 2:**
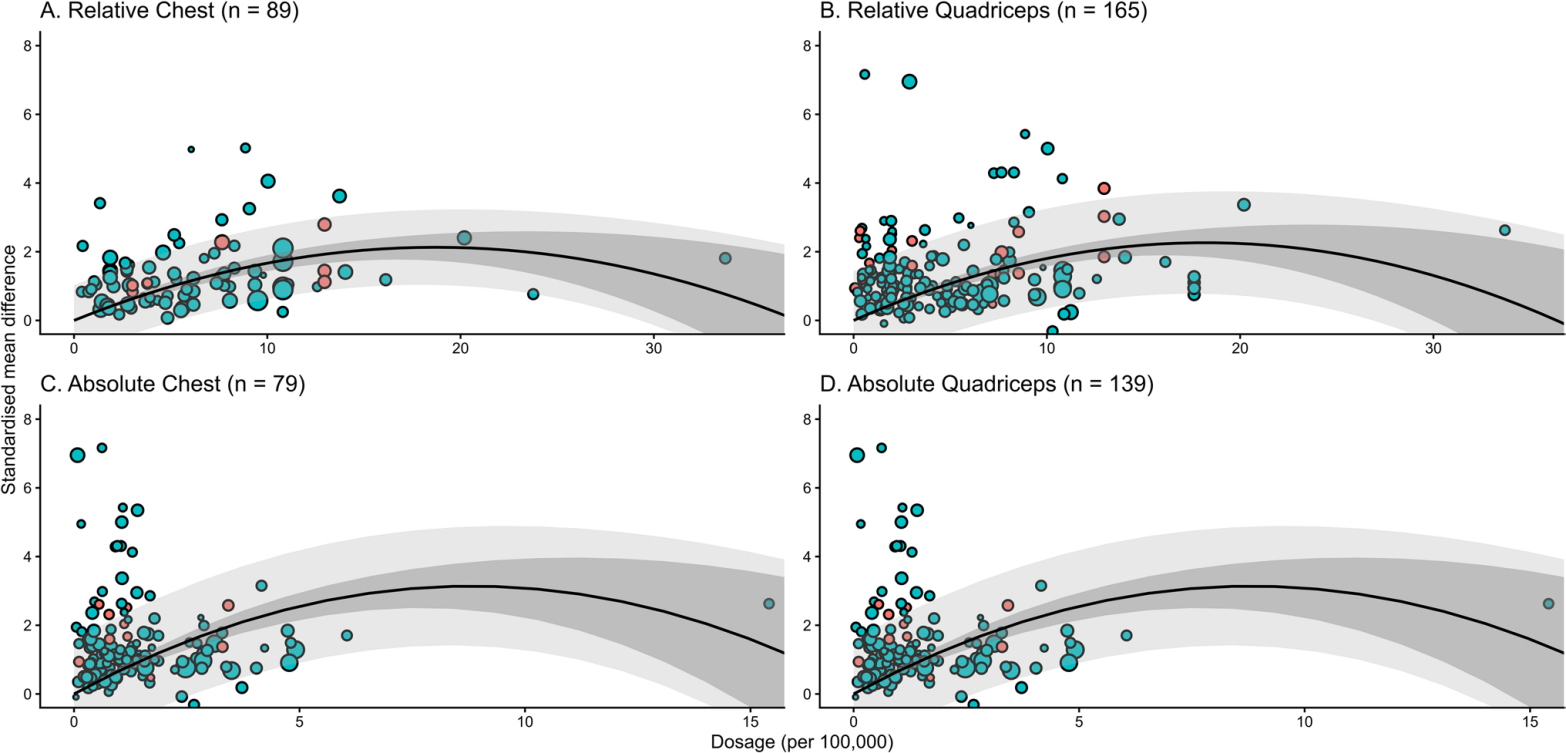
Quadratic meta-regression plots of resistance training dosage (per 100,000): **A** relative chest, **B** relative quadriceps, **C** absolute chest and **D** absolute quadriceps. The black line represents the meta-regression model; the dark grey shaded area represents the 95% confidence interval; the light grey shaded area represents the prediction interval; larger circles represent larger study sizes; standardised mean differences are calculated relative to each study’s control group; red circles represent trained populations and blue circles represent untrained populations

**Fig. 3:**
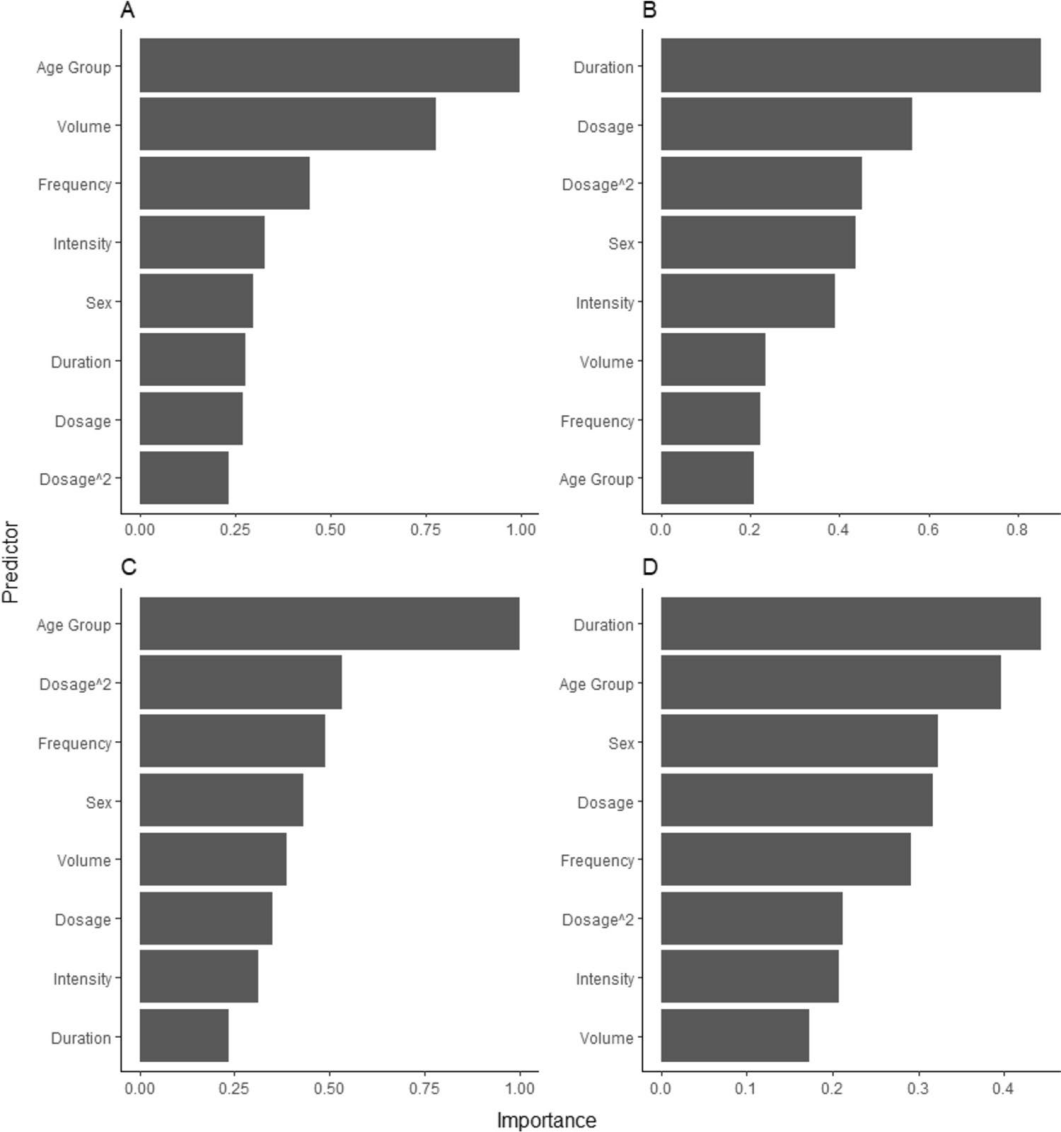
Variable importance plots for: **A** relative chest, **B** relative quadriceps, **C** absolute chest and **D** absolute quadriceps

The random effects meta-analysis comparing the effect of exercise with a non-exercise control group identified that RT increases muscle strength for all four outcomes (Table 2, Figs. 1–4 of the ESM). All data displayed significant heterogeneity and asymmetry (*p* < 0.01). The quality of evidence for these outcomes was high (Table 3 of the ESM).

**Table 2:** Meta-analysis for relative and absolute chest and quadriceps pooled studies

Outcome	*k*	SMD (95% CI)	Tau^2^ (95% CI)	I^2^ (95% CI)	Heterogeneity test	Asymmetry test^a^
Relative chest	89	1.2 (1.1–1.4)	0.4 (0.6–0.8)	0.8 (0.77–0.84)	*Q* = 417.6, *p* < 0.001	*t* = 4.1, *p* < 0.001
Relative quadriceps	165	1.3 (1.1–1.4)	0.4 (0.7–1.0)	0.8 (0.79–0.84)	*Q* = 790.9, *p* < 0.001	*t* = 6.4, *p* < 0.001
Absolute chest	79	1.2 (1.1–1.4)	0.4 (0.6–0.9)	0.8 (0.77–0.85)	*Q* = 390.7, *p* < 0.001	*t* = 3.9, *p* < 0.001
Absolute quadriceps	139	1.3 (1.2–1.5)	0.4 (0.8–1.0)	0.8 (0.79–0.85)	*Q* = 683.8, *p* < 0.001	*t* = 3.9, *p* < 0.001

*CI* confidence interval, *k* number of studies, *SMD* standardised mean difference

^a^Asymmetry assessed via Egger’s test

### Dose–Response Relationships Between Muscle Strength and RT Variables

The generalised additive models identified a small-to-moderate mostly linear significant effect for RT dosage for all four outcomes, where every unit increase in relative dosage was associated with an approximate 0.23–0.31 kg increase in chest and quadriceps strength, respectively, and every unit increase in absolute dosage was associated with a 0.96–1.10 kg increase in chest and quadriceps strength, respectively (Table 3, Models 1 and 2). The effect of dosage was attenuated when factoring frequency, sex and age into the model already containing volume, intensity and duration (Table 3, Model 3). Volume contributed meaningfully in a non-linear manner to chest strength with relative dosage and intensity contributing in a meaningful non-linear manner to quadriceps strength (both relative and absolute dosage) and chest strength with relative dosage. Older age groups experienced greater gains in strength with relative dosage for both chest and quadriceps outcomes. Training frequency (2 or 3 days) or being of male sex led to an increase in strength only for the quadriceps with absolute dosage.

**Table 3:** Comparing generalised additive models for the four outcomes

Model	Predictor	Relative chest		Relative quadriceps		Absolute chest		Absolute quadriceps	
		*b (SE)*	*e.df / r.df*	*b (SE)*	*e.df / r.df*	*b (SE)*	*e.df / r.df*	*b (SE)*	*e.df / r.df*
1	Dosage	0.23*** (0.02)	1.58***/ 1.82	0.31*** (0.02)	1.72***/1.92	0.96*** (0.09)	1.50***/1.75	1.10*** (0.09)	1.59***/1.83
2	Dosage	0.23*** (0.01)	1.23***/1.39	0.31*** (0.02)	1.67**/1.89	0.96*** (0.08)	1.37***/1.59	1.10*** (0.09)	1.39***/1.62
	Volume	–	7.92*/8.62	–	1.00 /1.00	–	1.78/2.22	–	2.31/2.84
	Intensity	–	8.22*/8.79	–	4.21 /5.21	–	1.00/1.00	–	3.71/4.58
	Duration	–	1.00*/1.00	–	1.00*/1.00	–	1.00/1.00	–	1.00/1.00
3	Dosage	0.07 (0.09)	0.31**/0.47	0.14 * (0.06)	0.75/0.97	0.03 (0.19)	0.97/0.63	0.68 (0.38)	0.86*/1.09
	Volume	0.01 (0.01)	6.99*/7.69	< 0.01 (< 0.01)	0.05/0.05	< 0.01 (0.01)	1.67/0.59	0.01 (< 0.01)	1.56/2.09
	Intensity	− 0.06 (0.04)	8.05*/8.62	0.03 * (0.01)	4.03/5.02	< 0.01 (< 0.01)	0.19/9.12	**< **− 0.01* (< 0.01)	2.98/3.85
	Duration	0.12* (0.04)	0.07**/0.07	− 0.04 (0.02)	0.09/0.09	0.03 (0.04)	0.26/6.56	0.22** (0.08)	0.33*/0.35
	Frequency [2]	− 0.26 (0.88)	–	− 0.12 (0.86)	–	− 0.31 (0.94)	–	1.64* (0.81)	–
	Frequency [3]	0.62 (0.85)	–	− 0.17 (0.87)	–	0.32 (0.94)	–	2.45** (0.83)	–
	Frequency [4]	1.14 (1.46)	–	− 0.32 (1.15)	–	–	–	1.98 (1.35)	–
	Sex [F]	0.01 (0.34)	–	− 0.48 (0.31)	–	− 0.6 (0.37)	–	− 0.86* (0.36)	–
	Age Group [O]	0.86* (0.37)	–	0.25 (0.34)	–	1.12* (0.41)	–	0.07 (0.42)	–

*b* model estimates, *e.df* effective degrees of freedom, *F* female, *r.df* reference degrees of freedom, *SE* standard error, ***p < 0.001; **p < 0.01; *p < 0.05

The e.df serves as a critical indicator of the flexibility and complexity of the smooth effects. A higher e.df suggests increased flexibility, allowing the smooth effect to capture intricate non-linear patterns in the data, while a lower e.df indicates a smoother, potentially more linear relationship. Bold text refers to statistically significant results

Reference category for Frequency is Frequency [1]

Reference category for Sex is Male [M]

Reference category for Age Group is [younger, < 65 years of age]

Figure 2 presents quadratic meta-regression plots of RT dosage for each of the four outcomes assessed, representing the proposed increasingly non-linear relationship towards the higher dosage ranges, from studies reporting trained and untrained populations. The regression models are limited by very few data sets reporting on dosage beyond 1,200,000 au (relative) and 500,000 kg (absolute). Model coefficients reflect a change per unit in dosage; however, for interpretability, Fig. 2 presents dosage scaled per 100,000 au. Breakpoints identified through segmented regression models (Fig. 5 of the ESM) represent changes in the slope of the dose–response curve and indicate transitions between phases of strength adaptation. Because of the wide and overlapping confidence intervals around breakpoints for absolute dosage models, a clear effective dosage cannot be estimated. In the relative dosage models, the first breakpoint (approximately 108,000 au for chest and 45,000 au for quadriceps) provides an estimate of where the rate of strength gain begins to slow. The final breakpoint (approximately 887,000 au for chest and 773,000 au for quadriceps) corresponds to the plateau or ‘optimal’ dosage region. Because of limited data at very high and very low dosages, uncertainty around these breakpoint estimates means they should be interpreted cautiously, particularly for the absolute models.

The overall quality of evidence examining the dose–response function between relative and absolute dosage and muscle strength was categorised as high. Concerns with risk of bias led to the downgrading of evidence to moderate. Egger’s regression test indicated funnel plot asymmetry (*p* < 0.001), suggesting a potential for publication bias; however, downgrading was not applied as the outcome measure, muscle strength, would favour a RT intervention group over a non-exercise control group. The remaining GRADE criteria did not result in further downgrading (Table 3 of the ESM). As a clear dose–response gradient was identified, the quality of evidence was upgraded; under rare circumstances, this is one of three criteria in the GRADE approach where the quality of evidence can be upgraded [22].

### RT Variable Importance

Table 4 presents the weighted averages of model estimates for various predictors across the four outcomes. Only age, for the relative and absolute chest, predicted development of muscle strength (*p* < 0.01). The weighted averages for the predictor variables are presented in VI plots (Fig. 3). Age and training volume were identified as important for developing chest strength when prescribing relative dosage, and training duration, dosage and age are important for developing quadriceps strength when prescribing absolute dosage. Despite intensity being identified as a significant predictor (in a non-linear manner) of muscle strength development through regression models, it was identified as being less important for developing muscle strength when prescribing both relative and absolute dosage.

**Table 4:** Unconditional model-averaged multivariate models for the four outcomes measured

Predictors	Relative chest				Relative quadriceps				Absolute chest				Absolute quadriceps			
	*b*	CI.lb	CI.ub	*p*-Value	*b*	CI.lb	CI.ub	*p*-value	*b*	CI.lb	CI.ub	*p*-value	*b*	CI.lb	CI.ub	*p*-Value
Intercept	0.627	− 0.611	1.866	0.321	1.38	0.461	2.299	0.003	1.044	0.42	1.668	0.001	1.688	1.231	2.144	0.001
Dosage (per 100,000)	0.009	− 0.051	0.069	0.774	0.033	− 0.052	0.117	0.449	− 0.001	− 0.26	0.258	0.993	− 0.05	− 0.311	0.21	0.705
Dosage^a^ (per 100,000)	< 0.01	− 0.002	0.002	0.9	0.001	− 0.003	0.005	0.586	− 0.016	− 0.065	0.032	0.51	0.002	− 0.042	0.045	0.936
Volume	0.002	< 0.01	0.003	0.123	< 0.01	− 0.001	0.001	0.948	0.001	− 0.001	0.003	0.48	< 0.01	− 0.001	0.001	0.822
Intensity	0.004	− 0.01	0.018	0.604	0.003	− 0.008	0.014	0.57	< 0.01	< 0.01	< 0.01	0.676	< 0.01	< 0.01	< 0.01	0.978
Duration	− 0.002	− 0.015	0.01	0.704	− 0.02	− 0.047	0.007	0.15	0.001	− 0.01	0.012	0.881	− 0.008	− 0.03	0.015	0.488
Frequency [2]	− 0.043	− 0.553	0.468	0.87	0.001	− 0.068	0.07	0.981	− 0.024	− 0.406	0.358	0.902	0.006	− 0.159	0.172	0.94
Frequency [3]	− 0.024	− 0.436	0.389	0.911	0.001	− 0.068	0.07	0.983	− 0.012	− 0.328	0.304	0.943	0.009	− 0.188	0.205	0.933
Frequency [4]	− 0.024	− 0.49	0.443	0.92	0.001	− 0.088	0.089	0.987	–	–	–	–	0.012	− 0.263	0.286	0.935
Sex [F]	− 0.044	− 0.306	0.218	0.745	− 0.109	− 0.453	0.235	0.535	− 0.109	− 0.477	0.259	0.561	− 0.085	− 0.448	0.278	0.646
Age Group [older]	0.539	0.136	0.943	0.009	0.016	− 0.205	0.238	0.886	0.703	0.254	1.152	0.002	0.12	− 0.311	0.551	0.586

Estimates represent the mean values for moderators across all 128 candidate models

*b* model estimates, *CI.lb* 95% confidence interval lower bound, *CI.ub* 95% confidence interval upper bound *F* female

^a^Dosage represents the quadratic term for dosage

Reference category for Frequency is Frequency [1]

Reference category for Sex is Male [M]

Reference category for Age Group is [younger, < 65 years of age]

## Discussion

The main findings of this study were that (1) a small-to-moderate, mostly linear significant relationship was identified between dosage and muscle strength development, with training volume and intensity contributing substantially in a non-linear manner; (2) we can predict the approximate dosage where muscle strength gains begin to taper (108,000 au [chest] and 45,000 au [quadriceps]), and plateau (887,000 au [chest] and 773,000 [quadriceps]), but cannot predict effective absolute dosage to improve strength; and (3) age, as well as training duration and volume are important prescription variables to consider when attempting to develop muscle strength.

The results of the current study support the notion that muscle strength development is aligned with the dosage prescribed, with clear benefits found up to a point where, thereafter, a plateau occurs. Interestingly, the plateau in strength development appeared similar regardless of the muscle group in the relative chest and quadriceps outcomes, suggesting that muscle strength adaptations occur at similar dosages regardless of the body part exercised. Previous systematic reviews unanimously support the notion that prolonged RT can result in a plateau in muscle strength [37–39]. The reason for a plateau in muscle strength development is still not fully understood, but may be the result of RT programmes not being varied enough over the duration of a training programme [40], overprescription of training variables leading to over-reaching and/or over-training [41], and/or a plateau in muscle hypertrophy [42]. With the identification of a dosage range where a plateau in muscle strength development can be anticipated, rather than waiting to be observed, RT variables can be manipulated in advance to promote additional muscle strength adaptation.

The present study identified duration, intensity and volume as likely predictors of muscle strength, with training frequency (in the absolute quadriceps), sex and age further modulating the response. These findings are supported by previous reviews in which volume [43, 44], frequency [43, 44], duration [12, 45] and intensity [9, 13] were reported to be important variables in developing upper-body and lower-body muscle strength. The data from the current study suggest that both training volume and intensity contribute in a curvilinear manner, highlighting the importance of evaluating these as continuous variables. Previous research, which categorised volume and intensity as either low (< 80%1RM, single set/exercise) or high (> 80%1RM, multiple set/exercise), has found significant changes in muscle strength irrespective of the category [9]. While an approach of analysing data categorically allows for faster and simpler analyses to be performed, it introduces the risk of missing the important thresholds that are likely to determine a response or the degree of response, particularly as it remains uncertain where those thresholds might be located to determine the importance of training volume and intensity. Therefore, it is important to consider the RT prescription as a holistic value, rather than an individual variable. Further consideration of how individual variables contribute to strength development and their appropriate weighting towards the dose, dosing and dosage metrics is required to determine an accurate and meaningful prescription. Similarly, the contributions of frequency and duration to muscle strength adaptation require further investigation to quantify their role. Frequency and duration of RT modulate the response of predictors by accumulating volume and exposure to training intensity throughout the week and training programme [43], thus increasing the overall dosage of RT. It is clear that RT dosage is related to the development of muscle strength in primarily untrained individuals; however, further development of the approach to determine RT dosage to guide RT prescription, including alternative training methods to those included in this review, is necessary.

Muscle strength can be developed utilising different methods from a traditional bilateral controlled approach used in this study, such as eccentric emphasis and neuromuscular stimuli (e.g. plyometrics). These approaches were not included in this review because of methodological differences and limited data availability, plus the fact that they are typically recommended for people experienced with RT. A large proportion of the participants included in studies reported in this review were untrained, and likely had low levels of muscle strength and familiarity with RT. Given these limitations, it can be deduced that duration, volume and intensity are variables that contribute to the development of muscle strength when prescribing traditional bilateral, rhythmic and controlled movements. Duration and volume were identified as important variables to develop muscle, and despite intensity being a significant predictor, it does not appear to be an important variable. This could be because of considering intensity as a linear contributor in the equation and not having it weighted appropriately, or it simply may be that RT volume and duration can overcome any limitations in the use of intensity. There are of course other variables that may influence strength development, such as training density and movement velocity, particularly in trained populations, but these were not considered in the dosage calculation because of limited data and practical constraints.

Sex had a limited influence on developing muscle strength, with only one of the four outcomes identified as having a significant effect on muscle strength. Anabolic hormone levels can influence muscle strength development, in particular absolute changes in strength [46], which could potentially lead to differences between the sexes. This review, however, focused on the relative change in muscle strength in predominantly untrained populations with findings similar to those reported by Roberts et al. [46] and Jones et al. [47], who found no differences between sexes for relative changes in lower-body and upper-body strength. A potential reason for why relative changes in muscle strength may be similar between sexes is that muscle protein synthesis and degradation rates are similar between male and female individuals, and that factors such as participant training status and/or lean muscle mass may impact muscle strength adaptations [48]. Furthermore, to our knowledge, this is the first study to demonstrate no differences in absolute changes in upper-body strength between sexes. Some potential reasons for this finding could be due to upper-body musculature being maintained to a greater extent than the lower body with an increase in age [49], and that male individuals lose muscle strength at twice the rate of female individuals [50]. The findings from this study demonstrate that sex has a limited influence on muscle strength, providing that dosage remains between 50,000 and 900,000 au.

Finally, it is apparent that age does not influence muscle strength development of the quadriceps outcome but does for the chest musculature. Previous literature has investigated the rates at which muscle strength declines in older adult populations, and reported that aging male individuals lose muscle strength at three times the rate that muscle mass is lost, and that female individuals retain muscle strength to a greater extent than their male counterparts as they age [50, 51]. It is not clear how representative the findings from this review are towards this potential sex factor with aging, as the studies included in this review with a participant age greater than 60 years had a male:female ratio of 1:3 for the quadriceps and 1:2 for the chest analysis. Smaller studies have found that age does not limit relative changes in muscle strength, and that changes in muscle strength among older populations may be attributed to improved muscle activation [49, 52]. The findings that indicate that age contributes to muscle strength development may be attributed to increased sedentary habits and reduced time spent at moderate-to-vigorous physical activity levels, leading to deconditioning; with older female individuals accumulating less moderate-to-vigorous intensity physical activity per day compared with older male individuals [53]. Mero et al. [54] reported that maximal strength can decrease up to 40% between the ages of 30 and 80 years, largely attributed to reductions in size and/or the number of fast-twitch muscle fibres. Considering that upper-body musculature is maintained to a greater extent than the lower body among aging individuals, it is possible that age-related differences in the upper body reflect the preservation of strength and neuromuscular function, and may therefore be more responsive to an RT stimulus. This indicates that neuromuscular mechanisms may contribute significantly to muscle strength development in older adults and that modification of volume, intensity and duration of RT might be effective in developing muscle strength [52], particularly in the chest musculature.

## Limitations

The constructed dosage variable represents a composite of core RT variables and serves as a proxy for a total accumulated training stimulus. While this allows for quantitative modelling of dose–response relationships, we recognise that such construction assumes additive and proportional relationships between training variables. This abstraction may not capture complex temporal or interactive effects between volume, intensity and other variables not included in this composite variable. Consequently, the observed relationships should be interpreted as statistical associations rather than mechanistic causal effects. There are several methodological limitations that are important to consider when interpreting the results from this systematic review and meta-analysis. First, this study makes assumptions about exercise programming from the eligible studies. We presumed that all exercises included within an intervention underwent maximal strength testing, and that a relative intensity prescription (i.e. 80% 1RM) was applied, unless the authors specified differently. However, we do not know for certain that these exercises underwent strength assessments because not all exercises had their 1RM data reported; this explains the difference in the number of studies between the relative and absolute outcomes (e.g. 164 studies in the relative quadriceps compared with 139 in the absolute quadriceps). This limitation is not necessarily a weakness of the approach of the study, but rather a limitation surrounding the reporting of data and generalisation of exercise programming within study methodologies. Our reporting has focused on major muscle groups of chest and quadriceps because of the inadequate reporting of strength outcomes for smaller muscle groups. It is not currently clear how much contribution each exercise included in the dosage calculations made to the reported strength outcomes. In addition, a single representative exercise per muscle group was selected to maintain statistical independence between studies, and this approach may not fully reflect the variability in exercise selection across trials. Second, the utilisation of a meta-analysis, although invaluable for synthesising research findings across multiple studies, is associated with several limitations, most notably, publication bias and heterogeneity due to the variability in study design, participant characteristics and potential publishing of positive or significant results over negative findings. While permutation methods enhance robustness by validating against random structurers, they cannot fully account for unexplained variability from unidentified effect modifiers. Third, a hierarchical tree was used to combine muscle strength data, and this could be considered a limitation as other researchers or practitioners may have differing opinions on the order or inclusion of particular exercises. However, two level-two strength and conditioning coaches registered with the Australian Strength and Conditioning Association were consulted for input on the hierarchical tree and we used this to build our model. Fourth, the findings from this study are primarily applicable to short-term interventions in predominantly untrained populations and do not account for a long-term training history, which is a key determinant of muscle strength development. Furthermore, other variables such as training density and movement velocity may influence strength development, but these were not included in the dosage construct because of limited data and practical constraints. Additionally, the rate of change in muscle strength for different muscle groups may vary depending on biomechanical factors and muscle fibre type distribution, which could influence the effects of the RT variables analysed. Last, trained participants were included in this review; however, only five studies from the chest, and 12 studies from the quadriceps analyses included sufficient data to calculate dosage. Comparisons between trained and untrained populations were not possible because of insufficient data and we cannot be certain that the optimal dosage for developing muscle strength is accurate for trained populations.

## Practical Applications and Future Research

Muscle strength can be effectively developed across a broad dosage range. Further, volume, intensity and duration of RT, factors in the composite dosage outcome, contribute significantly to muscle strength development. For individuals engaged in RT and those working in the health and fitness industry, the application of relative dosages is recommended because of similar optimal and effective dosage regions between muscle groups. Additionally, this study provides specific dosage targets where muscle strength can be effectively developed by broadly manipulating training variables to reach the dosage target, thus providing similar muscle strength development for untrained populations. For instance, when provided with two examples where the optimal dosage is prescribed across 16 weeks (the mean programme duration of the relative quadriceps analysis), (a) 5 sets of 5 repetitions across seven exercises at 80% 1RM, 4 sessions per week (RT dosage = 896,000 au) can have a similar dosage, and therefore effect on strength, to (b) 4 sets of 15 repetitions across eight exercises at 55% 1RM, 2 sessions per week (RT dosage = 844,800 au). Alternatively, across the minimum duration eligible in this study (4 weeks), a dosage of 50,400 au could comprise 3 sets of 7 repetitions across four exercises at 75% 1RM, 2 sessions per week. In addition, the novel methodological approach to investigating the dose–response function used in this study has application to other outcome measures, such as developing muscle mass and explosive power, as well as investigating this relationship across different populations (i.e. intermediate vs advanced weight-lifters). The dosage metric considers each prescription variable on its own merits and not how it changes through interactions with other prescription variables (i.e. dosage is typically lower when intensity is higher). Future research should explore appropriate strategies to more accurately consider and quantify the impact of individual prescription variables to quantify the training dosage. Future research should examine these dose–response relationships in athletic populations, where strength and conditioning coaches attempt to optimally prescribe RT to develop key outcome measures without over-training their athletes. Importantly, the limitations expressed in this review should be considered when investigating dose–response relationships because of the significant variability in study design and RT intervention methods that can influence outcomes such as strength development.

## Conclusions

The results from this systematic review and meta-analysis demonstrate a non-linear relationship between RT dosage and muscle strength. This study demonstrates that as dosage increases, the rate at which muscle strength develops increases up to approximately 870,000 au for the chest and 773,000 au for the quadriceps. After which, the rate of muscle strength development decreases, indicating that there is no further benefit to increasing dosage beyond these values in predominantly untrained populations. Resistance training volume, intensity and duration are important variables to increase muscle strength, and these prescription variables should be manipulated to create a prescription that provides an effective dosage that each individual can and will comply with.

## Supplementary Information

Below is the link to the electronic supplementary material.

Supplementary file1 (DOCX 51 KB)

Supplementary file2 (DOCX 42 KB)

Supplementary file3 (DOCX 19 KB)

Supplementary file4 (DOCX 73 KB)

Supplementary file5 (DOCX 74 KB)

Supplementary file6 (DOCX 91 KB)

Supplementary file7 (DOCX 89 KB)

Supplementary file8 (DOCX 139 KB)

Supplementary file9 Supplementary Figure 1. Forest plot comparing muscle strength development between exercise intervention and non-exercise control groups for the relative chest. (PNG 487 KB)

Supplementary file10 Supplementary Figure 2. Forest plot comparing muscle strength development between exercise intervention and non-exercise control groups for the absolute chest. (PNG 439 KB)

Supplementary file11 Supplementary Figure 3. Forest plot comparing muscle strength development between exercise intervention and non-exercise control groups for the relative quadriceps. (PNG 889 KB)

Supplementary file12 Supplementary Figure 4. Forest plot comparing muscle strength development between exercise intervention and non-exercise control groups for the absolute quadriceps. (PNG 770 KB)

Supplementary file13 Supplementary Figure 5. Segmented regression plots for the four outcomes; A = Relative Chest, B = Absolute Chest, C = Relative Quads, D = Absolute Quads. Black dots represent the different studies. The red line represents the segmented regression model. Breakpoints (represented by the dashed blue lines) display the point where the relationship between the outcome and Dosage changes, as per the segmented regression model. Number of breakpoints was determined via a sensitivity analysis of model fit, comparing models of varying break points. In Figure 5, A and B have two breakpoints, whereas C and D have three breakpoints. (PNG 6110 KB)

Supplementary file14 Supplementary File 1: References for All Studies (DOCX 72 KB)

Supplementary file15 Supplementary File 2: Meta-Data Spreadsheet (XLSX 100 KB)

Supplementary file16 Supplementary File 3: PRISMA Checklist (DOCX 35 KB)

## Declarations

### Availability of Data and Material

The datasets analysed during the current study are available in a FigShare repository (https://figshare.com/s/03f37d273d601fa8abcb).

### Code Availability

Meta-Data Spreadsheet, second workbook ‘Data Code & Translation Guide’.
