# Supplementary material for: The Influence of Individual Resistance Training Variables on Muscle Strength: A Systematic Review and Meta-analysis
Source: Sports Med. 2026 Apr 17;56(8):2001–15. doi: 10.1007/s40279-026-02432-5 (PMC13457492; doi:10.1007/s40279-026-02432-5)
Supplement: Supplementary file 1 [file 40279_2026_2432_MOESM14_ESM.docx]

**Reference list for all analyses**

1. Abe T, DeHoyos DV, Pollock ML, Garzarella L. Time course for strength and muscle thickness changes following upper and lower body resistance training in men and women. Eur J Appl Physiol. 2000;81(3):174-80.
2. Abdul-Al O, Zunquin G, El Hage R. Effects of Two Types of Resistance Training Modalities (Hypertrophy vs. Contrast Training) on Bone Parameters in a Group of Healthy Elderly Women. J Clin Densitom. 2024 Oct-Dec;27(4):101521. doi: 10.1016/j.jocd.2024.101521. Epub 2024 Aug 14. PMID: 39181062.
3. Ades PA, Ballor DL, Ashikaga T, Utton JL, Nair KS, Ades PA, et al. Weight training improves walking endurance in healthy elderly persons. Ann Intern Med. 1996;124(6):568-72.
4. Aguiar AF, Buzzachera CF, Pereira RM, Sanches VC, Januario RB, da Silva RA, et al. A single set of exhaustive exercise before resistance training improves muscular performance in young men. Eur J Appl Physiol. 2015;115(7):1589-99.
5. Ahtiainen JP, Hulmi JJ, Kraemer WJ, Lehti M, Nyman K, Selanne H, et al. Heavy resistance exercise training and skeletal muscle androgen receptor expression in younger and older men. Steroids. 2011;76(1-2):183-92.
6. Alcaraz PE, Perez-Gomez J, Chavarrias M, Blazevich AJ. Similarity in adaptations to high-resistance circuit vs. traditional strength training in resistance-trained men. J Strength Cond Res. 2011;25(9):2519-27.
7. Amiri E, Sheikholeslami-Vatani D. The role of resistance training and creatine supplementation on oxidative stress, antioxidant defense, muscle strength, and quality of life in older adults. Front Public Health. 2023 May 2;11:1062832. doi: 10.3389/fpubh.2023.1062832. PMID: 37206869; PMCID: PMC10189876.
8. Apel JM, Lacey RM, Kell RT. A comparison of traditional and weekly undulating periodized strength training programs with total volume and intensity equated. J Strength Cond Res. 2011;25(3):694-703.
9. Arazi H, Asadi A, Gentil P, Ramírez-Campillo R, Jahangiri P, Ghorbani A, Hackney AC, Zouhal H. Effects of different resistance training frequencies on body composition and muscular performance adaptations in men. PeerJ. 2021 Apr 21;9:e10537. doi: 10.7717/peerj.10537. PMID: 33976952; PMCID: PMC8067909.
10. Arsenis S, Gioftsidou A, Smilios I, Malliou P, Chatzinikolaou A, Ispyrlidis I, et al. Flywheel or free weight training for improvement of lower limbs strength? J Back Musculoskelet Rehabil. 2021;34(3):477-83.
11. Bagheri N, Bagheri R, Mesinovic J, Ghobadi H, Scott D, Kargarfard M, Dutheil F. Effects of Resistance Training on Muscular Adaptations and Inflammatory Markers in Overweight and Obese Men. Med Sci Sports Exerc. 2025 Mar 1;57(3):600-612. doi: 10.1249/MSS.0000000000003592. Epub 2024 Nov 15. PMID: 39809255; PMCID: PMC11801427.
12. Banks NF, Berry AC, Rogers EM, Jenkins NDM. Flywheel resistance training promotes unique muscle architectural and performance-related adaptations in young adults. Eur J Sport Sci. 2024 Dec;24(12):1765-1778. doi: 10.1002/ejsc.12215. Epub 2024 Nov 5. Erratum in: Eur J Sport Sci. 2025 Jun;25(6):e12316. doi: 10.1002/ejsc.12316. PMID: 39498804; PMCID: PMC11621374.
13. Bemben DA, Sherk VD, Buchanan SR, Kim S, Sherk K, Bemben MG. Acute and Chronic Bone Marker and Endocrine Responses to Resistance Exercise With and Without Blood Flow Restriction in Young Men. Front Physiol. 2022 Mar 17;13:837631. doi: 10.3389/fphys.2022.837631. PMID: 35370772; PMCID: PMC8969015.
14. Bijeh N, Mohammadnia-Ahmadi M, Hooshamnd-Moghadam B, Eskandari M, Golestani F. Effects of Soy Milk in Conjunction With Resistance Training on Physical Performance and Skeletal Muscle Regulatory Markers in Older Men. Biol Res Nurs. 2022 Jul;24(3):294-307. doi: 10.1177/10998004211073123. Epub 2022 Mar 25. PMID: 35332795.
15. Brandon LJ, Boyette LW, Lloyd A, Gaasch DA. Resistive training and long-term function in older adults. J Aging Phys Activity. 2004;12(1):10-28.
16. Brandon LJ, Sharon BF, Boyette LW, Anderson KA, Stiles R. Effects of resistive training on strength gains in older adults. J Nutr Health Aging. 1997;1(2):114-9.
17. Bell GJ, Syrotuik D, Martin TP, Burnham R, Quinney HA. Effect of concurrent strength and endurance training on skeletal muscle properties and hormone concentrations in humans. Eur J Appl Physiol. 2000;81(5):418-27.
18. Beltran Valls MR, Dimauro I, Brunelli A, Tranchita E, Ciminelli E, Caserotti P, et al. Explosive type of moderate-resistance training induces functional, cardiovascular, and molecular adaptations in the elderly. Age (Dordr). 2014;36(2):759-72.
19. Beneka A, Malliou P, Fatouros I, Jamurtas A, Gioftsidou A, Godolias G, et al. Resistance training effects on muscular strength of elderly are related to intensity and gender. J Sci Med Sport. 2005;8(3):274-83.
20. Bermon S, Philip P, Ferrari P, Candito M, Dolisi C. Effects of a short-term strength training programme on lymphocyte subsets at rest in elderly humans. Eur J Appl Physiol. 1999;79(4):336-40.
21. Bishop D, Jenkins DG. The influence of resistance training on the critical power function & time to fatigue at critical power. Aust J Sci Med Sport. 1996;28(4):101-5.
22. Bocalini DS, Serra AJ, dos Santos L, Murad N, Levy RF. Strength training preserves the bone mineral density of postmenopausal women without hormone replacement therapy. J Aging Health. 2009;21(3):519-27.
23. Botton CE, Radaelli R, Wilhelm EN, Rech A, Brown LE, Pinto RS. Neuromuscular Adaptations to Unilateral vs. Bilateral Strength Training in Women. J Strength Cond Res. 2016;30(7):1924-32.
24. Campos GE, Luecke TJ, Wendeln HK, Toma K, Hagerman FC, Murray TF, et al. Muscular adaptations in response to three different resistance-training regimens: specificity of repetition maximum training zones. Eur J Appl Physiol. 2002;88(1-2):50-60.
25. Calatayud J, Borreani S, Colado JC, Martin F, Tella V, Andersen LL. Bench press and push-up at comparable levels of muscle activity results in similar strength gains. J Strength Cond Res. 2015;29(1):246-53.
26. Carroll TJ, Abernethy PJ, Logan PA, Barber M, McEniery MT. Resistance training frequency: strength and myosin heavy chain responses to two and three bouts per week. Eur J Appl Physiol. 1998;78(3):270-5.
27. Cassemiro BM, Lemes ÍR, de Figueiredo MPF, Vanderlei FM, Pastre CM, Netto Júnior J. Effects of functional resistance training on muscle strength and musculoskeletal discomfort. Fisioterapia em Movimento. 2017;30(2):347-56.
28. Cesar Mde C, Borin JP, Gonelli PR, Simoes RA, de Souza TM, Montebelo MI. The effect of local muscle endurance training on cardiorespiratory capacity in young women. J Strength Cond Res. 2009;23(6):1637-43.
29. Charette SL, McEvoy L, Pyka G, Snow-Harter C, Guido D, Wiswell RA, et al. Muscle hypertrophy response to resistance training in older women. J Appl Physiol. 1991;70(5):1912-6.
30. Chtourou H, Driss T, Souissi S, Gam A, Chaouachi A, Souissi N. The effect of strength training at the same time of the day on the diurnal fluctuations of muscular anaerobic performances. J Strength Cond Res. 2012;26(1):217-25.
31. Coetsee C, Terblanche E. The time course of changes induced by resistance training and detraining on muscular and physical function in older adults. Eur. 2015;12:7.
32. Colakoglu M, Cam FS, Kayitken B, Cetinoz F, Colakoglu S, Turkmen M, et al. ACE genotype may have an effect on single versus multiple set preferences in strength training. Eur J Appl Physiol. 2005;95(1):20-6.
33. Conceicao MS, Bonganha V, Vechin FC, Berton RP, Lixandrao ME, Nogueira FR, et al. Sixteen weeks of resistance training can decrease the risk of metabolic syndrome in healthy postmenopausal women. Clin Interv Aging. 2013;8:1221-8.
34. Cook SB, Scott BR, Hayes KL, Murphy BG. Neuromuscular Adaptations to Low-Load Blood Flow Restricted Resistance Training. J Sports Sci Med. 2018;17(1):66-73.
35. Cormie P, McGuigan MR, Newton RU. Changes in the eccentric phase contribute to improved stretch-shorten cycle performance after training. Med Sci Sports Exerc. 2010;42(9):1731-44.
36. Correa CS, LaRoche DP, Cadore EL, Reischak-Oliveira A, Bottaro M, Kruel LF, et al. 3 Different types of strength training in older women. Int J Sports Med. 2012;33(12):962-9.
37. Correa CS, Teixeira BC, Cobos RC, Macedo RC, Kruger RL, Carteri RB, et al. High-volume resistance training reduces postprandial lipaemia in postmenopausal women. J Sports Sci. 2015;33(18):1890-901.
38. Cunha PM, Nunes JP, Tomeleri CM, Nascimento MA, Schoenfeld BJ, Antunes M, et al. Resistance Training Performed With Single and Multiple Sets Induces Similar Improvements in Muscular Strength, Muscle Mass, Muscle Quality, and IGF-1 in Older Women: A Randomized Controlled Trial. J Strength Cond Res. 2020;34(4):1008-16.
39. de Lima C, Boullosa DA, Frollini AB, Donatto FF, Leite RD, Gonelli PR, et al. Linear and daily undulating resistance training periodizations have differential beneficial effects in young sedentary women. Int J Sports Med. 2012;33(9):723-7.
40. de Oliveira Júnior GN, de Sousa JFR, Carneiro M, Martins FM, Santagnello SB, Orsatti FL. Resistance training-induced improvement in exercise tolerance is not dependent on muscle mass gain in post-menopausal women. Eur J Sport Sci. 2021;21(7):958-66.
41. de Souza EO, Tricoli V, Aoki MS, Roschel H, Brum PC, Bacurau AV, et al. Effects of concurrent strength and endurance training on genes related to myostatin signaling pathway and muscle fiber responses. J Strength Cond Res. 2014;28(11):3215-23.
42. de Souza EO, Tricoli V, Roschel H, Brum PC, Bacurau AV, Ferreira JC, et al. Molecular adaptations to concurrent training. Int J Sports Med. 2013;34(3):207-13.
43. Dobbs TJ, Simonson SR, Conger SA. Improving power output in older adults using plyometrics in a body mass--supported tread-mill. Journal of Strength & Conditioning Research (Lippincott Williams & Wilkins). 2018;32(9):2458-65.
44. Dong-il SEO, Wi-Young SO, Dong Jun S. Effect of a low-intensity resistance exercise programme with blood flow restriction on growth hormone and insulin-like growth factor-1 levels in middle-aged women. South African Journal for Research in Sport, Physical Education & Recreation. 2016;38(2):167-77.
45. Donges CE, Duffield R, Guelfi KJ, Smith GC, Adams DR, Edge JA. Comparative effects of single-mode vs. duration-matched concurrent exercise training on body composition, low-grade inflammation, and glucose regulation in sedentary, overweight, middle-aged men. Applied Physiology, Nutrition & Metabolism. 2013;38(7):779-88.
46. Early KS, Rockhill M, Bryan A, Tyo B, Buuck D, McGinty J. Effect of blood flow restriction training on muscular performance, pain and vascular function. Int J Sports Phys Ther. 2020;15(6):892-900.
47. Eidukaitė, S., Masiulis, N., & Kvedaras, M. Exploring the Preliminary Effects of Resistance Training on Total Brain-Derived Neurotrophic Factor (BDNF) Levels in Elderly Individuals: A Pilot Study. Baltic Journal of Sport and Health Sciences. (2023).
48. Elliott KJ, Sale C, Cable NT. Effects of resistance training and detraining on muscle strength and blood lipid profiles in postmenopausal women. BJSM online. 2002;36(5):340-4.
49. Englund DA, Price LL, Grosicki GJ, Iwai M, Kashiwa M, Liu C, et al. Progressive Resistance Training Improves Torque Capacity and Strength in Mobility-Limited Older Adults. J Gerontol A Biol Sci Med Sci. 2019;74(8):1316-21.
50. Evangelista AL, Teixeira CVS, Barros BM, de Azevedo JB, Paunksnis MRR, Souza CR, et al. Does whole-body electrical muscle stimulation combined with strength training promote morphofunctional alterations? Clinics. 2019;74:e1334.
51. Eyre, J. C., Weyer J, Geisler S, Gavanda S. Comparison of the effects of isokinetic and isotonic strength training in team sport athletes: A randomized controlled trial. J Biomech. 2024 Aug;173:112246. doi: 10.1016/j.jbiomech.2024.112246. Epub 2024 Jul 23. PMID: 39067184.
52. Farinatti PT, Geraldes AA, Bottaro MF, Lima MV, Albuquerque RB, Fleck SJ. Effects of different resistance training frequencies on the muscle strength and functional performance of active women older than 60 years. J Strength Cond Res. 2013;27(8):2225-34.
53. Fatouros IG, Kambas A, Katrabasas I, Leontsini D, Chatzinikolaou A, Jamurtas AZ, et al. Resistance training and detraining effects on flexibility performance in the elderly are intensity-dependent. J Strength Cond Res. 2006;20(3):634-42.
54. Fatouros IG, Kambas A, Katrabasas I, Nikolaidis K, Chatzinikolaou A, Leontsini D, et al. Strength training and detraining effects on muscular strength, anaerobic power, and mobility of inactive older men are intensity dependent. BJSM online. 2005;39(10):776-80.
55. Fatouros IG, Taxildaris K, Tokmakidis SP, Kalapotharakos V, Aggelousis N, Athanasopoulos S, et al. The effects of strength training, cardiovascular training and their combination on flexibility of inactive older adults. Int J Sports Med. 2002;23(2):112-9.
56. Fernandez-Lezaun E, Schumann M, Makinen T, Kyrolainen H, Walker S. Effects of resistance training frequency on cardiorespiratory fitness in older men and women during intervention and follow-up. Exp Gerontol. 2017;95:44-53.
57. Ferraresi C, de Brito Oliveira T, de Oliveira Zafalon L, de Menezes Reiff RB, Baldissera V, de Andrade Perez SE, et al. Effects of low level laser therapy (808 nm) on physical strength training in humans. Lasers Med Sci. 2011;26(3):349-58.
58. Filho MM, Venturini GRDO, Moreira OC, Leitão L, Mira PAC, de Castro JBP, Aidar FJ, Novaes JDS, Vianna JM, Caputo Ferreira ME. Effects of Different Types of Resistance Training and Detraining on Functional Capacity, Muscle Strength, and Power in Older Women: A Randomized Controlled Study. J Strength Cond Res. 2022 Apr 1;36(4):984-990. doi: 10.1519/JSC.0000000000004195. PMID: 35319001.
59. Fimland MS, Helgerud J, Gruber M, Leivseth G, Hoff J. Functional maximal strength training induces neural transfer to single-joint tasks. Eur J Appl Physiol. 2009;107(1):21-9.
60. Fisher J, Bruce-Low S, Smith D. A randomized trial to consider the effect of Romanian deadlift exercise on the development of lumbar extension strength. Phys Ther Sport. 2013;14(3):139-45.
61. Fragala MS, Jajtner AR, Beyer KS, Townsend JR, Emerson NS, Scanlon TC, et al. Biomarkers of muscle quality: N-terminal propeptide of type III procollagen and C-terminal agrin fragment responses to resistance exercise training in
62. Gerage AM, Forjaz CL, Nascimento MA, Januario RS, Polito MD, Cyrino ES. Cardiovascular adaptations to resistance training in elderly postmenopausal women. Int J Sports Med. 2013;34(9):806-13.
63. Gomes de Souza Vale R, Dias Ferrão ML, de Alkmim Moreira Nunes R, Baptista da Silva J, Nodari Júnior RJ, Martin Dantas EH. muscle strength, gh and igf-1 in older women submitted to land and aquatic resistance training. Revista Brasileira de Medicina do Esporte. 2017;23(4):274-8.
64. Goto K, Ishii N, Kizuka T, Takamatsu K. The impact of metabolic stress on hormonal responses and muscular adaptations. Med Sci Sports Exerc. 2005;37(6):955-63.
65. Griffen C, Duncan M, Hattersley J, Weickert MO, Dallaway A, Renshaw D. Effects of resistance exercise and whey protein supplementation on skeletal muscle strength, mass, physical function, and hormonal and inflammatory biomarkers in healthy active older men: a randomised, double-blind, placebo-controlled trial. Exp Gerontol. 2022 Feb;158:111651. doi: 10.1016/j.exger.2021.111651. Epub 2021 Dec 9. PMID: 34896568.
66. Gualano B, Macedo AR, Alves CR, Roschel H, Benatti FB, Takayama L, de Sá Pinto AL, Lima FR, Pereira RM. Creatine supplementation and resistance training in vulnerable older women: a randomized double-blind placebo-controlled clinical trial. Exp Gerontol. 2014 May;53:7-15. doi: 10.1016/j.exger.2014.02.003. Epub 2014 Feb 13. PMID: 24530883.
67. Hagerman FC, Walsh SJ, Hagerman FC, Walsh SJ, Staron RS, Hikida RS, et al. Effects of high-intensity resistance training on untrained older men. I. Strength, cardiovascular, and metabolic responses. Journals of Gerontology Series A: Biological Sciences & Medical Sciences. 2000;55(7):B336-B46.
68. Harber MP, Fry AC, Rubin MR, Smith JC, Weiss LW. Skeletal muscle and hormonal adaptations to circuit weight training in untrained men. Scand J Med Sci Sports. 2004;14(3):176-85.
69. Haykowsky M, Humen D, Teo K, Quinney A, Souster M, Bell G, et al. Effects of 16 weeks of resistance training on left ventricular morphology and systolic function in healthy men >60 years of age. Am J Cardiol. 2000;85(8):1002-6.
70. Hendrickson NR, Sharp MA, Alemany JA, Walker LA, Harman EA, Spiering BA, Hatfield DL, Yamamoto LM, Maresh CM, Kraemer WJ, Nindl BC. Combined resistance and endurance training improves physical capacity and performance on tactical occupational tasks. Eur J Appl Physiol. 2010 Aug;109(6):1197-208. doi: 10.1007/s00421-010-1462-2. Epub 2010 Apr 10. PMID: 20383644.
71. Henwood TR, Riek S, Taaffe DR. Strength versus muscle power-specific resistance training in community-dwelling older adults. J Gerontol A Biol Sci Med Sci. 2008;63(1):83-91.
72. Henwood TR, Taaffe DR. Short-term resistance training and the older adult: the effect of varied programmes for the enhancement of muscle strength and functional performance. Clin Physiol Funct Imaging. 2006;26(5):305-13.
73. Herda AA, Nabavizadeh O. Short-term resistance training in older adults improves muscle quality: A randomized control trial. Exp Gerontol. 2021;145:111195.
74. Holviala J, Hakkinen A, Alen M, Sallinen J, Kraemer W, Hakkinen K. Effects of prolonged and maintenance strength training on force production, walking, and balance in aging women and men. Scand J Med Sci Sports. 2014;24(1):224-33.
75. Holviala J, Kraemer WJ, Sillanpaa E, Karppinen H, Avela J, Kauhanen A, et al. Effects of strength, endurance and combined training on muscle strength, walking speed and dynamic balance in aging men. Eur J Appl Physiol. 2012;112(4):1335-47.
76. Hooshmand-Moghadam B, Eskandari M, Golestani F, Rezae S, Mahmoudi N, Gaeini AA. The effect of 12-week resistance exercise training on serum levels of cellular aging process parameters in elderly men. Exp Gerontol. 2020 Nov;141:111090. doi: 10.1016/j.exger.2020.111090. Epub 2020 Sep 9. PMID: 32919015.
77. Huschtscha Z, Parr A, Porter J, Costa RJS. The Effects of a High-Protein Dairy Milk Beverage With or Without Progressive Resistance Training on Fat-Free Mass, Skeletal Muscle Strength and Power, and Functional Performance in Healthy Active Older Adults: A 12-Week Randomized Controlled Trial. Front Nutr. 2021;8:644865.
78. Jeong J, Choi DH, Song Y, Shin CS. Muscle Strength Training Alters Muscle Activation of the Lower Extremity during Side-Step Cutting in Females. J Mot Behav. 2020;52(6):703-12.
79. Kalapotharakos V, Smilios I, Parlavatzas A, Tokmakidis SP. The effect of moderate resistance strength training and detraining on muscle strength and power in older men. J Geriatr Phys Ther. 2007;30(3):109-13.
80. Kalapotharakos VI, Michalopoulou M, Godolias G, Tokmakidis SP, Malliou PV, Gourgoulis V. The effects of high- and moderate-resistance training on muscle function in the elderly. J Aging Phys Activity. 2004;12(2):131-43.
81. Karabulut M, Abe T, Sato Y, Bemben MG. The effects of low-intensity resistance training with vascular restriction on leg muscle strength in older men. Eur J Appl Physiol. 2010;108(1):147-55.
82. Katsoulis K, Amara CE. The Effects of Power Training Frequency on Muscle Power and Functional Performance in Older Women: A Randomized Controlled Trial. J Strength Cond Res. 2023 Nov 1;37(11):2289-2297. doi: 10.1519/JSC.0000000000004527. Epub 2023 Jun 19. PMID: 37335760.
83. Kell RT. The influence of periodized resistance training on strength changes in men and women. J Strength Cond Res. 2011;25(3):735-44.
84. Kim E, Dear A, Ferguson SL, Seo D, Bemben MG. Effects of 4 weeks of traditional resistance training vs. superslow strength training on early phase adaptations in strength, flexibility, and aerobic capacity in college-aged women. J Strength Cond Res. 2011;25(11):3006-13.
85. Kirk EP, Washburn RA, Bailey BW, LeCheminant JD, Donnelly JE. Six months of supervised high-intensity low-volume resistance training improves strength independent of changes in muscle mass in young overweight men. J Strength Cond Res. 2007;21(1):151-6.
86. Kraemer WJ, Nindl BC, Ratamess NA, Gotshalk LA, Volek JS, Fleck SJ, et al. Changes in muscle hypertrophy in women with periodized resistance training. Med Sci Sports Exerc. 2004;36(4):697-708.
87. Lamas L, Ugrinowitsch C, Rodacki A, Pereira G, Mattos EC, Kohn AF, et al. Effects of strength and power training on neuromuscular adaptations and jumping movement pattern and performance. J Strength Cond Res. 2012;26(12):3335-44.
88. Lanza MB, Prado GC, Lacerda LT, Dinardi RR, Carvalho Junior LH, Diniz RC, Lima FV, Chagas MH, Martins-Costa HC. Muscle hypertrophy response across four muscles involved in the bench press exercise: Randomized 10 weeks training intervention. J Bodyw Mov Ther. 2024 Oct;40:1417-1422. doi: 10.1016/j.jbmt.2024.07.054. Epub 2024 Jul 22. PMID: 39593465.
89. Lee SR, Directo D, Khamoui AV. Fish oil administration combined with resistance exercise training improves strength, resting metabolic rate, and inflammation in older adults. Aging Clin Exp Res. 2022 Dec;34(12):3073-3081. doi: 10.1007/s40520-022-02250-5. Epub 2022 Sep 22. PMID: 36136236.
90. Leite T, de Souza Teixeira A, Saavedra F, Leite RD, Rhea MR, Simao R. Influence of strength and flexibility training, combined or isolated, on strength and flexibility gains. J Strength Cond Res. 2015;29(4):1083-8.
91. Letieri RV, Teixeira AM, Furtado GE, Lamboglia CG, Rees JL, Gomes BB. Effect of 16weeks of resistance exercise and detraining comparing two methods of blood flow restriction in muscle strength of healthy older women: A randomized controlled trial. Exp Gerontol. 2018;114:78-86.
92. Libardi CA, De Souza GV, Cavaglieri CR, Madruga VA, Chacon-Mikahil MP. Effect of resistance, endurance, and concurrent training on TNF-alpha, IL-6, and CRP. Med Sci Sports Exerc. 2012;44(1):50-6.
93. Libardi C, Souza G, GÁspari A, Santos CD, Leite S, Dias R, et al. Effects of concurrent training on interleukin-6, tumour necrosis factor-alpha and C-reactive protein in middle-aged men. J Sports Sci. 2011;29(14):1573-81.
94. Lixandrao ME, Damas F, Chacon-Mikahil MP, Cavaglieri CR, Ugrinowitsch C, Bottaro M, et al. Time Course of Resistance Training-Induced Muscle Hypertrophy in the Elderly. J Strength Cond Res. 2016;30(1):159-63.
95. Lo MS, Lin LL, Yao WJ, Ma MC. Training and detraining effects of the resistance vs. endurance program on body composition, body size, and physical performance in young men. J Strength Cond Res. 2011;25(8):2246-54.
96. Lopes PB, Pereira G, Lodovico A, Bento PCB, Rodacki ALF. Strength and Power Training Effects on Lower Limb Force, Functional Capacity, and Static and Dynamic Balance in Older Female Adults. Rejuvenation Res. 2016;19(5):385-93.
97. Manocchia P, Spierer DK, Lufkin AK, Minichiello J, Castro J. Transference of kettlebell training to strength, power, and endurance. J Strength Cond Res. 2013;27(2):477-84.
98. Martín-Hernández J, Marín PJ, Menéndez H, Ferrero C, Loenneke JP, Herrero AJ. Muscular adaptations after two different volumes of blood flow-restricted training. Scand J Med Sci Sports. 2013;23(2):e114-20.
99. Marx JO, Ratamess NA, Nindl BC, Gotschalk LA, Volek JS, Dohi K, et al. Low-volume circuit versus high-volume periodized resistance training in women. Med Sci Sports Exerc. 2001;33(4):635-43.
100. Maté-Muñoz JL, Monroy Antón AJ, Jodra Jiménez P, Garnacho-Castaño MV. Effects of Instability versus Traditional Resistance Training on Strength, Power and Velocity in Untrained Men. J Sports Sci Med. 2014;13(3):460-8.
101. McBride JM, Blaak JB, Triplett-McBride T. Effect of resistance exercise volume and complexity on EMG, strength, and regional body composition. Eur J Appl Physiol. 2003;90(5-6):626-32.
102. McCartney N, Hicks AL, Martin J, Webber CE. Long-term resistance training in the elderly: effects on dynamic strength, exercise capacity, muscle, and bone. J Gerontol A Biol Sci Med Sci. 1995;50(2):B97-104.
103. McGinley C, Jensen RL, Byrne CA, Shafat A. Early-phase strength gains during traditional resistance training compared with an upper-body air-resistance training device. J Strength Cond Res. 2007;21(2):621-7.
104. Mohammad Ali A, Bahram A, Maghsoud P, Mohammad Javad R, Stannard SR. Public health. Effects of Combined Aerobic and Resistant Training on Lipid Profile and Glycemic Control in Sedentary Men. International Medical Journal. 2014;21(2):132-6.
105. Morgan B, Mirza AM, Gimblet CJ, Ortlip AT, Ancalmo J, Kalita D, Pellinger TK, Walter JM, Werner TJ. Effect of an 11-Week Resistance Training Program on Arterial Stiffness in Young Women. J Strength Cond Res. 2023 Feb 1;37(2):315-321. doi: 10.1519/JSC.0000000000004280. Epub 2022 Jul 1. PMID: 35916876.
106. Mosti MP, Carlsen T, Aas E, Hoff J, Stunes AK, Syversen U. Maximal strength training improves bone mineral density and neuromuscular performance in young adult women. J Strength Cond Res. 2014;28(10):2935-45.
107. Nascimento de Oliveira-Júnior G, de Sousa JFR, Carneiro M, Martins FM, Santagnello SB, Souza MVC, et al. Resistance Training Volume Enhances Muscle Hypertrophy, but Not Strength in Postmenopausal Women: A Randomized Controlled Trial. J Strength Cond Res. 2022;36(5):1216-21.
108. Nichols JF, Hitzelberger LM, Sherman JG, Patterson P. Effects of resistance training on muscular strength and functional abilities of community-dwelling older adults. J Aging Phys Activity. 1995;3(3):238-50.
109. Nichols JF, Omizo DK, Peterson KK, Nelson KP. Efficacy of heavy-resistance training for active women over sixty: muscular strength, body composition, and program adherence. J Am Geriatr Soc. 1993;41(3):205-10.
110. Nunes PRP, Barcelos LC, Oliveira AA, Furlanetto R, Jr., Martins FM, Resende E, et al. Muscular Strength Adaptations and Hormonal Responses After Two Different Multiple-Set Protocols of Resistance Training in Postmenopausal Women. J Strength Cond Res. 2019;33(5):1276-85.
111. Olson TP, Dengel DR, Leon AS, Schmitz KH. Moderate resistance training and vascular health in overweight women. Med Sci Sports Exerc. 2006;38(9):1558-64.
112. Panton LB, Franke WD, Bleil DA, Baier SM, King DS. Effects of resistance training on cardiovascular responses to lower body negative pressure in the elderly. Clin Physiol. 2001;21(5):605-11.
113. Pinto R, Correa C, Radaelli R, Cadore E, Brown L, Bottaro M. Short-term strength training improves muscle quality and functional capacity of elderly women. Age (Dordr). 2014;36(1):365-72.
114. Prestes J, da Cunha Nascimento D, Tibana RA, Teixeira TG, Vieira DC, Tajra V, et al. Understanding the individual responsiveness to resistance training periodization. Age (Dordr). 2015;37(3):9793.
115. Pyka G, Lindenberger E, Charette S, Marcus R. Muscle strength and fiber adaptations to a year-long resistance training program in elderly men and women. J Gerontol. 1994;49(1):M22-7.
116. Rabelo HT, Oliveira RJ, Bottaro M. Effects of resistance training on activities of daily living in older women. Biol Sport. 2004;21(4):325-36.
117. Ramirez-Campillo R, Castillo A, de la Fuente CI, Campos-Jara C, Andrade DC, Alvarez C, et al. High-speed resistance training is more effective than low-speed resistance training to increase functional capacity and muscle performance in older women. Exp Gerontol. 2014;58:51-7.
118. Rana SR, Chleboun GS, Gilders RM, Hagerman FC, Herman JR, Hikida RS, et al. Comparison of early phase adaptations for traditional strength and endurance, and low velocity resistance training programs in college-aged women. J Strength Cond Res. 2008;22(1):119-27.
119. Reis JG, Costa GC, Schmidt A, Ferreira CH, Abreu DC. Do muscle strengthening exercises improve performance in the 6-minute walk test in postmenopausal women? Rev. 2012;16(3):236-40.
120. Rhodes EC, Martin AD, Taunton JE, Donnelly M, Warren J, Elliot J. Effects of one year of resistance training on the relation between muscular strength and bone density in elderly women. BJSM online. 2000;34(1):18-22.
121. Santos E, Rhea MR, Simao R, Dias I, de Salles BF, Novaes J, et al. Influence of moderately intense strength training on flexibility in sedentary young women. J Strength Cond Res. 2010;24(11):3144-9.
122. Santos LC, Cherem EH, Azeredo FP, Neves EB, Oliveira DR, Silva AJ, et al. Effects of different strength training programs in young males maximal strength and anthropometrics. Motricidade. 2018;14(1S):301-9.
123. Schlumberger A, Stec J, Schmidtbleicher D. Single- vs. multiple-set strength training in women. J Strength Cond Res. 2001;15(3):284-9.
124. Schneider SM, Amonette WE, Blazine K, Bentley J, Lee SM, Loehr JA, et al. Training with the International Space Station interim resistive exercise device. Med Sci Sports Exerc. 2003;35(11):1935-45.
125. Seo J, Park HY, Jung WS, Kim SW, Sun Y, Choi JH, Kim J, Lim K. Effects of 12 Weeks of Resistance Training on Body Composition, Muscle Hypertrophy and Function, Blood Lipid Level, and Hemorheological Properties in Middle-Aged Obese Women. Rev Cardiovasc Med. 2023 Jul 12;24(7):196. doi: 10.31083/j.rcm2407196. PMID: 39077025; PMCID: PMC11262441.
126. Shabkhiz F, Khalafi M, Rosenkranz S, Karimi P, Moghadami K. Resistance training attenuates circulating FGF-21 and myostatin and improves insulin resistance in elderly men with and without type 2 diabetes mellitus: A randomized controlled clinical trial. EJSS (Champaign). 2020:1-14.
127. Sharman MJ, Newton RU, Triplett-McBride T, McGuigan MR, McBride JM, Hakkinen A, et al. Changes in myosin heavy chain composition with heavy resistance training in 60- to 75-year-old men and women. Eur J Appl Physiol. 2001;84(1-2):127-32.
128. Shaw BS, Shaw I, Brown GA. Comparison of resistance and concurrent resistance and endurance training regimes in the development of strength. J Strength Cond Res. 2009;23(9):2507-14.
129. Simao R, Lemos A, Salles B, Leite T, Oliveira E, Rhea M, et al. The influence of strength, flexibility, and simultaneous training on flexibility and strength gains. J Strength Cond Res. 2011;25(5):1333-8.
130. Simao R, Spineti J, de Salles BF, Matta T, Fernandes L, Fleck SJ, et al. Comparison between nonlinear and linear periodized resistance training: hypertrophic and strength effects. J Strength Cond Res. 2012;26(5):1389-95.
131. Simons R, Andel R. The effects of resistance training and walking on functional fitness in advanced old age. J Aging Health. 2006;18(1):91-105.
132. Snow-Harter C, Bouxsein ML, Lewis BT, Carter DR, Marcus R. Effects of resistance and endurance exercise on bone mineral status of young women: a randomized exercise intervention trial. J Bone Miner Res. 1992;7(7):761-9.
133. Spineti J, de Salles BF, Rhea MR, Lavigne D, Matta T, Miranda F, et al. Influence of exercise order on maximum strength and muscle volume in nonlinear periodized resistance training. J Strength Cond Res. 2010;24(11):2962-9.
134. Strandberg E, Edholm P, Ponsot E, Wahlin-Larsson B, Hellmen E, Nilsson A, et al. Influence of combined resistance training and healthy diet on muscle mass in healthy elderly women: a randomized controlled trial. J Appl Physiol. 2015;119(8):918-25.
135. Taaffe DR, Duret C, Wheeler S, Marcus R. Once-weekly resistance exercise improves muscle strength and neuromuscular performance in older adults. J Am Geriatr Soc. 1999;47(10):1208-14.
136. Tanimoto M, Sanada K, Yamamoto K, Kawano H, Gando Y, Tabata I, et al. Effects of whole-body low-intensity resistance training with slow movement and tonic force generation on muscular size and strength in young men. J Strength Cond Res. 2008;22(6):1926-38.
137. Teixeira, B. C., Boeno, F.P., Siqueira, C.V., Cadore, E. L., Ribeiro, J. L., Reischak-Oliveira, A., dos Santos Cunha, G. Strength training enhances endothelial and muscular function in postmenopausal women. Science & Sports. 2019; 34(2), p. e147-e154. ISSN 0765-1597, https://doi.org/10.1016/j.scispo.2018.09.006.
138. Terzis G, Georgiadis G, Stratakos G, Vogiatzis I, Kavouras S, Manta P, et al. Resistance exercise-induced increase in muscle mass correlates with p70S6 kinase phosphorylation in human subjects. Eur J Appl Physiol. 2008;102(2):145-52.
139. Terrazas L, Montalvo S, Dorgo S. Manual resistance training and resistance training elicit neuromuscular strength adaptations but not body composition changes in an 8- week exercise intervention. J Bodyw Mov Ther. 2025 Jun;42:810-815. doi: 10.1016/j.jbmt.2025.01.042. Epub 2025 Feb 1. PMID: 40325759.
140. Tieland M, Verdijk LB, de Groot LC, van Loon LJ. Handgrip strength does not represent an appropriate measure to evaluate changes in muscle strength during an exercise intervention program in frail older people. Int J Sport Nutr Exerc Metab. 2015;25(1):27-36.
141. Toien T, Pedersen Haglo H, Unhjem R, Hoff J, Wang E. Maximal strength training: the impact of eccentric overload. J Neurophysiol. 2018;120(6):2868-76.
142. Toien T, Haglo H, Nyberg SK, Rao SV, Stunes AK, Mosti MP, Wang E. Maximal strength training-induced increase in efferent neural drive is not reflected in relative protein expression of SERCA. Eur J Appl Physiol. 2021 Dec;121(12):3421-3430. doi: 10.1007/s00421-021-04807-0. Epub 2021 Sep 8. PMID: 34498135; PMCID: PMC8571128.
143. Tomeleri CM, Ribeiro AS, Nunes JP, Schoenfeld BJ, Souza MF, Schiavoni D, Junior PS, Cavaglieri CR, Cunha PM, Venturini D, Barbosa DS, Cyrino ES. Influence of Resistance Training Exercise Order on Muscle Strength, Hypertrophy, and Anabolic Hormones in Older Women: A Randomized Controlled Trial. J Strength Cond Res. 2020 Nov;34(11):3103-3109. doi: 10.1519/JSC.0000000000003147. PMID: 33105360.
144. Tracy BL, Enoka RM. Steadiness training with light loads in the knee extensors of elderly adults. Med Sci Sports Exerc. 2006;38(4):735-45.
145. Ünlü G, Çevikol C, Melekoglu T. Comparison of the Effects of Eccentric, Concentric, and Eccentric-Concentric Isotonic Resistance Training at Two Velocities on Strength and Muscle Hypertrophy. Journal of Strength & Conditioning Research (Lippincott Williams & Wilkins). 2020;34(2):337-44.
146. Van Roie E, Walker S, Van Driessche S, Delabastita T, Vanwanseele B, Delecluse C. An age-adapted plyometric exercise program improves dynamic strength, jump performance and functional capacity in older men either similarly or more than traditional resistance training. PLoS ONE. 2020;15(8):e0237921.
147. Vechin FC, Libardi CA, Conceicao MS, Damas FR, Lixandrao ME, Berton RP, et al. Comparisons between low-intensity resistance training with blood flow restriction and high-intensity resistance training on quadriceps muscle mass and strength in elderly. J Strength Cond Res. 2015;29(4):1071-6.
148. Vincent KR, Braith RW, Feldman RA, Magyari PM, Cutler RB, Persin SA, et al. Resistance exercise and physical performance in adults aged 60 to 83. J Am Geriatr Soc. 2002;50(6):1100-7.
149. Walker S, Hulmi JJ, Wernbom M, Nyman K, Kraemer WJ, Ahtiainen JP, et al. Variable resistance training promotes greater fatigue resistance but not hypertrophy versus constant resistance training. Eur J Appl Physiol. 2013;113(9):2233-44.
150. Wallerstein LF, Tricoli V, Barroso R, Rodacki AL, Russo L, Aihara AY, et al. Effects of strength and power training on neuromuscular variables in older adults. J Aging Phys Activity. 2012;20(2):171-85.
151. Whitehead MT, Scheett TP, McGuigan MR, Martin AV. A Comparison of the Effects of Short-Term Plyometric and Resistance Training on Lower-Body Muscular Performance. J Strength Cond Res. 2018;32(10):2743-9.
152. Willoughby DS. Effects of heavy resistance training on myostatin mRNA and protein expression. Med Sci Sports Exerc. 2004;36(4):574-82.
153. Willoughby DS, Pelsue SC. Muscle strength and qualitative myosin heavy chain isoform mRNA expression in the elderly after moderate- and high-intensity weight training. J Aging Phys Activity. 1998;6(4):327-39.
154. Willoughby DS, Rosene J. Effects of oral creatine and resistance training on myosin heavy chain expression. Med Sci Sports Exerc. 2001;33(10):1674-81.
155. Wilson GJ, Murphy AJ, Glorgi A. Weight and plyometric training: effects on eccentric and concentric force production. Can J Appl Physiol. 1996;21(4):301-15.
156. Wood RH, Reyes R, Welsch MA, Favaloro-Sabatier J, Sabatier M, Lee CM, et al. Concurrent cardiovascular and resistance training in healthy older adults. Med Sci Sports Exerc. 2001;33(10):1751-8.
157. Yasuda T, Ogasawara R, Sakamaki M, Bemben MG, Abe T. Relationship between limb and trunk muscle hypertrophy following high-intensity resistance training and blood flow-restricted low-intensity resistance training. Clin Physiol Funct Imaging. 2011;31(5):347-51.
