# Supplementary material for: The Influence of Individual Resistance Training Variables on Muscle Strength: A Systematic Review and Meta-analysis
Source: Sports Med. 2026 Apr 17;56(8):2001–15. doi: 10.1007/s40279-026-02432-5 (PMC13457492; doi:10.1007/s40279-026-02432-5)
Supplement: Supplementary file 3 [file 40279_2026_2432_MOESM1_ESM.docx]

**Supplementary Table 1.** Search terms used to identify articles in CINAHL, MEDLINE, and SPORTDiscus

| **Population** | **Intervention** | **Comparison** | **Outcomes** |
| --- | --- | --- | --- |
| *Elderly*  *Young Adult*  *Older Adult*  *Middle-Age*  *Adult* | *Resistance Training*  *Resistance Exercise*  *Weight Training*  *Weight Lifting*  *Strength Training*  *Free Weight*  *High Intensity Training*  *Progressive Resistance*  *Physical Resistance*  *Weighted Exercise*  *Resistance Program*  *Resistive Load*  *Body Building*  *Power Lifting*  *Training Frequency*  *Training Volume*  *Training Load*  *Training Intensity* | *Non-Exercise Control Group*  *Placebo and Non-Exercise Control Group*  *Stretching and Mobility Control Group*  *Non-Exercising Off-Season Athletes* | *Muscle Strength*  *Muscular Strength*  *Muscle Morphology*  *1 RM*  *Repetition Max**  *Rep* Max**  *Physical Strength*  *Force Development*  *Max* Strength* |
|  |  |  |  |

**Note.** RM = repetition maximum
