# Supplementary material for: The Influence of Individual Resistance Training Variables on Muscle Strength: A Systematic Review and Meta-analysis
Source: Sports Med. 2026 Apr 17;56(8):2001–15. doi: 10.1007/s40279-026-02432-5 (PMC13457492; doi:10.1007/s40279-026-02432-5)
Supplement: Supplementary file 4 [file 40279_2026_2432_MOESM2_ESM.docx]

Scopus:

| Term | Citations |
| --- | --- |
| TITLE-ABS-KEY  (Elderly) | 1,029,256 |
| TITLE-ABS-KEY  (“young adult”) | 1,342,339 |
| TITLE-ABS-KEY  (“older adults”) | 193,829 |
| TITLE-ABS-KEY  (Middle-age) | 1,006,961 |
| TITLE-ABS-KEY  (Adult) | 10,576,756 |
| ( TITLE-ABS-KEY ( elderly ) ) OR ( TITLE-ABS-KEY ( "young adult" ) ) OR ( TITLE-ABS-KEY ( "older adult" ) ) OR ( TITLE-ABS-KEY ( middle-age ) ) AND ( TITLE-ABS-KEY ( adult ) ) | 11,142,780 |
| TITLE-ABS-KEY (“resistance training”) | 36,563 |
| TITLE-ABS-KEY (“resistance exercise”) | 11,849 |
| TITLE-ABS-KEY (“weight training”) | 3,252 |
| TITLE-ABS-KEY (“weight lifting”) | 8,622 |
| TITLE-ABS-KEY (“strength training”) | 12,329 |
| TITLE-ABS-KEY (“free weight”) | 1,315 |
| TITLE-ABS-KEY (“high intensity training”) | 1,579 |
| TITLE-ABS-KEY (“progressive resistance”) | 1,550 |
| TITLE-ABS-KEY (“physical resistance”) | 1,992 |
| TITLE-ABS-KEY (“weighted exercise”) | 15 |
| TITLE-ABS-KEY (“resistive load”) | 5,291 |
| TITLE-ABS-KEY (“body building”) | 1,620 |
| TITLE-ABS-KEY (“power lifting”) | 115 |
| TITLE-ABS-KEY  (“training frequency”) | 1,085 |
| TITLE-ABS-KEY (“training volume”) | 2,491 |
| TITLE-ABS-KEY (“training load”) | 5,322 |
| TITLE-ABS-KEY (“training intensity”) | 3,012 |
| ( TITLE-ABS-KEY ( "resistance training" ) ) OR ( TITLE-ABS-KEY ( "resistance exercise" ) ) OR ( TITLE-ABS-KEY ( "weight training" ) ) OR ( TITLE-ABS-KEY ( "weight lifting" ) ) OR ( TITLE-ABS-KEY ( "strength training" ) ) OR ( TITLE-ABS-KEY ( "free weight" ) ) OR ( TITLE-ABS-KEY ( "high intensity training" ) ) OR ( TITLE-ABS-KEY ( "progressive resistance" ) ) OR ( TITLE-ABS-KEY ( "physical resistance" ) ) OR ( TITLE-ABS-KEY ( "weighted exercise" ) ) OR ( TITLE-ABS-KEY ( "resistive load" ) ) OR ( TITLE-ABS-KEY ( "body building" ) ) OR ( TITLE-ABS-KEY ( "power lifting" ) ) OR ( TITLE-ABS-KEY ( "training frequency" ) ) OR ( TITLE-ABS-KEY ( "training volume" ) ) OR ( TITLE-ABS-KEY ( "training load" ) ) OR ( TITLE-ABS-KEY ( "training intensity" ) ) | 73,393 |
| TITLE-ABS-KEY (“muscle strength”) | 105,331 |
| TITLE-ABS-KEY (“muscular strength”) | 7,184 |
| TITLE-ABS-KEY (“muscle morphology”) | 1,826 |
| TITLE-ABS-KEY (“1 RM”) | 2,147 |
| TITLE-ABS-KEY (“repetition max*”) | 10,504 |
| TITLE-ABS-KEY (“physical strength”) | 3,821 |
| TITLE-ABS-KEY (“force development”) | 4,765 |
| TITLE-ABS-KEY (“max* strength”) | 9,442 |
| ( TITLE-ABS-KEY ( "muscle strength" ) ) OR ( TITLE-ABS-KEY ( "muscular strength" ) ) OR ( TITLE-ABS-KEY ( "muscle morphology" ) ) OR ( TITLE-ABS-KEY ( "1 RM" ) ) OR ( TITLE-ABS-KEY ( "repetition max*" ) ) OR ( TITLE-ABS-KEY ( "rep* max*" ) ) OR ( TITLE-ABS-KEY ( "physical strength" ) ) OR ( TITLE-ABS-KEY ( "force development" ) ) OR ( TITLE-ABS-KEY ( "max* strength" ) ) | 130,684 |
| ( ( TITLE-ABS-KEY ( elderly ) ) OR ( TITLE-ABS-KEY ( "young adult" ) ) OR ( TITLE-ABS-KEY ( "older adult" ) ) OR ( TITLE-ABS-KEY ( middle-age ) ) OR ( TITLE-ABS-KEY ( adult ) ) ) AND ( ( TITLE-ABS-KEY ( "resistance training" ) ) OR ( TITLE-ABS-KEY ( "resistance exercise" ) ) OR ( TITLE-ABS-KEY ( "weight training" ) ) OR ( TITLE-ABS-KEY ( "weight lifting" ) ) OR ( TITLE-ABS-KEY ( "strength training" ) ) OR ( TITLE-ABS-KEY ( "free weight" ) ) OR ( TITLE-ABS-KEY ( "high intensity training" ) ) OR ( TITLE-ABS-KEY ( "progressive resistance" ) ) OR ( TITLE-ABS-KEY ( "physical resistance" ) ) OR ( TITLE-ABS-KEY ( "weighted exercise" ) ) OR ( TITLE-ABS-KEY ( "resistive load" ) ) OR ( TITLE-ABS-KEY ( "body building" ) ) OR ( TITLE-ABS-KEY ( "power lifting" ) ) OR ( TITLE-ABS-KEY ( "training frequency" ) ) OR ( TITLE-ABS-KEY ( "training volume" ) ) OR ( TITLE-ABS-KEY ( "training load" ) ) OR ( TITLE-ABS-KEY ( "training intensity" ) ) ) AND ( ( TITLE-ABS-KEY ( "muscle strength" ) ) OR ( TITLE-ABS-KEY ( "muscular strength" ) ) OR ( TITLE-ABS-KEY ( "muscle morphology" ) ) OR ( TITLE-ABS-KEY ( "1 RM" ) ) OR ( TITLE-ABS-KEY ( "repetition max*" ) ) OR ( TITLE-ABS-KEY ( "rep* max*" ) ) OR ( TITLE-ABS-KEY ( "physical strength" ) ) OR ( TITLE-ABS-KEY ( "force development" ) ) OR ( TITLE-ABS-KEY ( "max* strength" ) ) ) | 13,680 |
| ( ( TITLE-ABS-KEY ( elderly ) ) OR ( TITLE-ABS-KEY ( "young adult" ) ) OR ( TITLE-ABS-KEY ( "older adult" ) ) OR ( TITLE-ABS-KEY ( middle-age ) ) OR ( TITLE-ABS-KEY ( adult ) ) ) AND ( ( TITLE-ABS-KEY ( "resistance training" ) ) OR ( TITLE-ABS-KEY ( "resistance exercise" ) ) OR ( TITLE-ABS-KEY ( "weight training" ) ) OR ( TITLE-ABS-KEY ( "weight lifting" ) ) OR ( TITLE-ABS-KEY ( "strength training" ) ) OR ( TITLE-ABS-KEY ( "free weight" ) ) OR ( TITLE-ABS-KEY ( "high intensity training" ) ) OR ( TITLE-ABS-KEY ( "progressive resistance" ) ) OR ( TITLE-ABS-KEY ( "physical resistance" ) ) OR ( TITLE-ABS-KEY ( "weighted exercise" ) ) OR ( TITLE-ABS-KEY ( "resistive load" ) ) OR ( TITLE-ABS-KEY ( "body building" ) ) OR ( TITLE-ABS-KEY ( "power lifting" ) ) OR ( TITLE-ABS-KEY ( "training frequency" ) ) OR ( TITLE-ABS-KEY ( "training volume" ) ) OR ( TITLE-ABS-KEY ( "training load" ) ) OR ( TITLE-ABS-KEY ( "training intensity" ) ) ) AND ( ( TITLE-ABS-KEY ( "muscle strength" ) ) OR ( TITLE-ABS-KEY ( "muscular strength" ) ) OR ( TITLE-ABS-KEY ( "muscle morphology" ) ) OR ( TITLE-ABS-KEY ( "1 RM" ) ) OR ( TITLE-ABS-KEY ( "repetition max*" ) ) OR ( TITLE-ABS-KEY ( "rep* max*" ) ) OR ( TITLE-ABS-KEY ( "physical strength" ) ) OR ( TITLE-ABS-KEY ( "force development" ) ) OR ( TITLE-ABS-KEY ( "max* strength" ) ) ) AND ( LIMIT-TO ( DOCTYPE , "ar" ) ) | 12,252 |
| ( ( TITLE-ABS-KEY ( elderly ) ) OR ( TITLE-ABS-KEY ( "young adult" ) ) OR ( TITLE-ABS-KEY ( "older adult" ) ) OR ( TITLE-ABS-KEY ( middle-age ) ) OR ( TITLE-ABS-KEY ( adult ) ) ) AND ( ( TITLE-ABS-KEY ( "resistance training" ) ) OR ( TITLE-ABS-KEY ( "resistance exercise" ) ) OR ( TITLE-ABS-KEY ( "weight training" ) ) OR ( TITLE-ABS-KEY ( "weight lifting" ) ) OR ( TITLE-ABS-KEY ( "strength training" ) ) OR ( TITLE-ABS-KEY ( "free weight" ) ) OR ( TITLE-ABS-KEY ( "high intensity training" ) ) OR ( TITLE-ABS-KEY ( "progressive resistance" ) ) OR ( TITLE-ABS-KEY ( "physical resistance" ) ) OR ( TITLE-ABS-KEY ( "weighted exercise" ) ) OR ( TITLE-ABS-KEY ( "resistive load" ) ) OR ( TITLE-ABS-KEY ( "body building" ) ) OR ( TITLE-ABS-KEY ( "power lifting" ) ) OR ( TITLE-ABS-KEY ( "training frequency" ) ) OR ( TITLE-ABS-KEY ( "training volume" ) ) OR ( TITLE-ABS-KEY ( "training load" ) ) OR ( TITLE-ABS-KEY ( "training intensity" ) ) ) AND ( ( TITLE-ABS-KEY ( "muscle strength" ) ) OR ( TITLE-ABS-KEY ( "muscular strength" ) ) OR ( TITLE-ABS-KEY ( "muscle morphology" ) ) OR ( TITLE-ABS-KEY ( "1 RM" ) ) OR ( TITLE-ABS-KEY ( "repetition max*" ) ) OR ( TITLE-ABS-KEY ( "rep* max*" ) ) OR ( TITLE-ABS-KEY ( "physical strength" ) ) OR ( TITLE-ABS-KEY ( "force development" ) ) OR ( TITLE-ABS-KEY ( "max* strength" ) ) ) AND ( LIMIT-TO ( DOCTYPE , "ar" ) ) AND ( LIMIT-TO ( LANGUAGE , "English" ) ) | 11,972 |
| ( ( TITLE-ABS-KEY ( elderly ) ) OR ( TITLE-ABS-KEY ( "young adult" ) ) OR ( TITLE-ABS-KEY ( "older adult" ) ) OR ( TITLE-ABS-KEY ( middle-age ) ) OR ( TITLE-ABS-KEY ( adult ) ) ) AND ( ( TITLE-ABS-KEY ( "resistance training" ) ) OR ( TITLE-ABS-KEY ( "resistance exercise" ) ) OR ( TITLE-ABS-KEY ( "weight training" ) ) OR ( TITLE-ABS-KEY ( "weight lifting" ) ) OR ( TITLE-ABS-KEY ( "strength training" ) ) OR ( TITLE-ABS-KEY ( "free weight" ) ) OR ( TITLE-ABS-KEY ( "high intensity training" ) ) OR ( TITLE-ABS-KEY ( "progressive resistance" ) ) OR ( TITLE-ABS-KEY ( "physical resistance" ) ) OR ( TITLE-ABS-KEY ( "weighted exercise" ) ) OR ( TITLE-ABS-KEY ( "resistive load" ) ) OR ( TITLE-ABS-KEY ( "body building" ) ) OR ( TITLE-ABS-KEY ( "power lifting" ) ) OR ( TITLE-ABS-KEY ( "training frequency" ) ) OR ( TITLE-ABS-KEY ( "training volume" ) ) OR ( TITLE-ABS-KEY ( "training load" ) ) OR ( TITLE-ABS-KEY ( "training intensity" ) ) ) AND ( ( TITLE-ABS-KEY ( "muscle strength" ) ) OR ( TITLE-ABS-KEY ( "muscular strength" ) ) OR ( TITLE-ABS-KEY ( "muscle morphology" ) ) OR ( TITLE-ABS-KEY ( "1 RM" ) ) OR ( TITLE-ABS-KEY ( "repetition max*" ) ) OR ( TITLE-ABS-KEY ( "rep* max*" ) ) OR ( TITLE-ABS-KEY ( "physical strength" ) ) OR ( TITLE-ABS-KEY ( "force development" ) ) OR ( TITLE-ABS-KEY ( "max* strength" ) ) ) AND ( LIMIT-TO ( EXACTKEYWORD , "Randomized Controlled Trial" ) ) AND ( LIMIT-TO ( LANGUAGE , "English" ) ) | 4,207 |

SportsDiscus:

| Term | Citations |
| --- | --- |
| TITLE-ABS-KEY  (Elderly) | 14,942 |
| TITLE-ABS-KEY  (“young adult”) | 23,682 |
| TITLE-ABS-KEY  (“older adults”) | 15,984 |
| TITLE-ABS-KEY  (Middle-age) | 6,589 |
| TITLE-ABS-KEY  (Adult) | 95,770 |
| (elderly OR young adult OR older adult OR middle-age AND adult) | 43,292 |
| TITLE-ABS-KEY (“resistance training”) | 15,403 |
| TITLE-ABS-KEY (“resistance exercise”) | 7,823 |
| TITLE-ABS-KEY (“weight training”) | 14,634 |
| TITLE-ABS-KEY (“weight lifting”) | 10,499 |
| TITLE-ABS-KEY (“strength training”) | 18,593 |
| TITLE-ABS-KEY (“free weight”) | 1,488 |
| TITLE-ABS-KEY (“high intensity training”) | 5,910 |
| TITLE-ABS-KEY (“progressive resistance”) | 692 |
| TITLE-ABS-KEY (“physical resistance”) | 698 |
| TITLE-ABS-KEY (“weighted exercise”) | 131 |
| TITLE-ABS-KEY (“resistive load”) | 102 |
| TITLE-ABS-KEY (“body building”) | 930 |
| TITLE-ABS-KEY (“power lifting”) | 1,846 |
| TITLE-ABS-KEY  (“training frequency”) | 1,534 |
| TITLE-ABS-KEY (“training volume”) | 3,463 |
| TITLE-ABS-KEY (“training load”) | 5,982 |
| TITLE-ABS-KEY (“training intensity”) | 10,179 |
| (elderly OR young adult OR older adult OR middle-age OR adult) AND (resistance training OR resistance exercise OR weight training OR weight lifting OR strength training OR free weight OR high intensity training OR progressive resistance OR physical resistance OR weighted exercise OR resistive load OR body building OR power lifting OR training frequency OR training volume OR training load OR training intensity) | 5,257 |
| TITLE-ABS-KEY (“muscle strength”) | 32,198 |
| TITLE-ABS-KEY (“muscular strength”) | 4,074 |
| TITLE-ABS-KEY (“muscle morphology”) | 605 |
| TITLE-ABS-KEY (“1 RM”) | 733 |
| TITLE-ABS-KEY (“repetition max*”) | 4,930 |
| TITLE-ABS-KEY (“rep* max*”) | 7,118 |
| TITLE-ABS-KEY (“physical strength”) | 3,517 |
| TITLE-ABS-KEY (“force development”) | 2,040 |
| TITLE-ABS-KEY (“max* strength”) | 5,985 |
| (muscle strength OR muscular strength OR muscle morphology OR 1 RM OR repetition max* OR rep* max* OR physical strength OR force development OR max* strength) | 98,573 |
| (elderly OR young adult OR older adult OR middle-age AND adult) AND (resistance training OR resistance exercise OR weight training OR weight lifting OR strength training OR free weight OR high intensity training OR progressive resistance OR physical resistance OR weighted exercise OR resistive load OR body building OR power lifting OR training frequency OR training volume OR training load OR training intensity) AND (muscle strength OR muscular strength OR muscle morphology OR 1 RM OR repetition max* OR rep* max* OR physical strength OR force development OR max* strength) | 2,371 |
| (elderly OR young adult OR older adult OR middle-age AND adult) AND (resistance training OR resistance exercise OR weight training OR weight lifting OR strength training OR free weight OR high intensity training OR progressive resistance OR physical resistance OR weighted exercise OR resistive load OR body building OR power lifting OR training frequency OR training volume OR training load OR training intensity) AND (muscle strength OR muscular strength OR muscle morphology OR 1 RM OR repetition max* OR rep* max* OR physical strength OR force development OR max* strength)  Limiters: Peer Reviewed | 2,112 |
| (elderly OR young adult OR older adult OR middle-age AND adult) AND (resistance training OR resistance exercise OR weight training OR weight lifting OR strength training OR free weight OR high intensity training OR progressive resistance OR physical resistance OR weighted exercise OR resistive load OR body building OR power lifting OR training frequency OR training volume OR training load OR training intensity) AND (muscle strength OR muscular strength OR muscle morphology OR 1 RM OR repetition max* OR rep* max* OR physical strength OR force development OR max* strength)  Limiters: Peer Reviewed  English | 1,978 |
| (elderly OR young adult OR older adult OR middle-age AND adult) AND (resistance training OR resistance exercise OR weight training OR weight lifting OR strength training OR free weight OR high intensity training OR progressive resistance OR physical resistance OR weighted exercise OR resistive load OR body building OR power lifting OR training frequency OR training volume OR training load OR training intensity) AND (muscle strength OR muscular strength OR muscle morphology OR 1 RM OR repetition max* OR rep* max* OR physical strength OR force development OR max* strength)  Limiters: Peer Reviewed  English  Academic Journal | 1973 |

CINAHL:

| Term | Citations |
| --- | --- |
| TITLE-ABS-KEY  (Elderly) | 102,177 |
| TITLE-ABS-KEY  (“young adult”) | 292,516 |
| TITLE-ABS-KEY  (“older adults”) | 72,846 |
| TITLE-ABS-KEY  (Middle-age) | 1,073,415 |
| TITLE-ABS-KEY  (Adult) | 1,462,730 |
| (elderly OR young adult OR older adult OR middle-age AND adult) | 994,319 |
| TITLE-ABS-KEY (“resistance training”) | 8,826 |
| TITLE-ABS-KEY (“resistance exercise”) | 4,646 |
| TITLE-ABS-KEY (“weight training”) | 1,960 |
| TITLE-ABS-KEY (“weight lifting”) | 3,181 |
| TITLE-ABS-KEY (“strength training”) | 5,490 |
| TITLE-ABS-KEY (“free weight”) | 862 |
| TITLE-ABS-KEY (“high intensity training”) | 2,869 |
| TITLE-ABS-KEY (“progressive resistance”) | 841 |
| TITLE-ABS-KEY (“physical resistance”) | 629 |
| TITLE-ABS-KEY (“weighted exercise”) | 74 |
| TITLE-ABS-KEY (“resistive load”) | 91 |
| TITLE-ABS-KEY (“body building”) | 773 |
| TITLE-ABS-KEY (“power lifting”) | 64 |
| TITLE-ABS-KEY  (“training frequency”) | 1,052 |
| TITLE-ABS-KEY (“training volume”) | 1,438 |
| TITLE-ABS-KEY (“training load”) | 1,759 |
| TITLE-ABS-KEY (“training intensity”) | 4,744 |
| (elderly OR young adult OR older adult OR middle-age OR adult) AND (resistance training OR resistance exercise OR weight training OR weight lifting OR strength training OR free weight OR high intensity training OR progressive resistance OR physical resistance OR weighted exercise OR resistive load OR body building OR power lifting OR training frequency OR training volume OR training load OR training intensity) | 29,429 |
| TITLE-ABS-KEY (“muscle strength”) | 26,389 |
| TITLE-ABS-KEY (“muscular strength”) | 2,045 |
| TITLE-ABS-KEY (“muscle morphology”) | 546 |
| TITLE-ABS-KEY (“1 RM”) | 290 |
| TITLE-ABS-KEY (“repetition max*”) | 1,829 |
| TITLE-ABS-KEY (“rep* max*”) | 5,589 |
| TITLE-ABS-KEY (“physical strength”) | 3,321 |
| TITLE-ABS-KEY (“force development”) | 1,035 |
| TITLE-ABS-KEY (“max* strength”) | 3,475 |
| (muscle strength OR muscular strength OR muscle morphology OR 1 RM OR repetition max* OR rep* max* OR physical strength OR force development OR max* strength) | 165,574 |
| (elderly OR young adult OR older adult OR middle-age AND adult) AND (resistance training OR resistance exercise OR weight training OR weight lifting OR strength training OR free weight OR high intensity training OR progressive resistance OR physical resistance OR weighted exercise OR resistive load OR body building OR power lifting OR training frequency OR training volume OR training load OR training intensity) AND (muscle strength OR muscular strength OR muscle morphology OR 1 RM OR repetition max* OR rep* max* OR physical strength OR force development OR max* strength) | 5,684 |
| (elderly OR young adult OR older adult OR middle-age AND adult) AND (resistance training OR resistance exercise OR weight training OR weight lifting OR strength training OR free weight OR high intensity training OR progressive resistance OR physical resistance OR weighted exercise OR resistive load OR body building OR power lifting OR training frequency OR training volume OR training load OR training intensity) AND (muscle strength OR muscular strength OR muscle morphology OR 1 RM OR repetition max* OR rep* max* OR physical strength OR force development OR max* strength)  Limiters: Peer Reviewed | 5,595 |
| (elderly OR young adult OR older adult OR middle-age AND adult) AND (resistance training OR resistance exercise OR weight training OR weight lifting OR strength training OR free weight OR high intensity training OR progressive resistance OR physical resistance OR weighted exercise OR resistive load OR body building OR power lifting OR training frequency OR training volume OR training load OR training intensity) AND (muscle strength OR muscular strength OR muscle morphology OR 1 RM OR repetition max* OR rep* max* OR physical strength OR force development OR max* strength)  Limiters: Peer Reviewed  English | 5,478 |
| (elderly OR young adult OR older adult OR middle-age AND adult) AND (resistance training OR resistance exercise OR weight training OR weight lifting OR strength training OR free weight OR high intensity training OR progressive resistance OR physical resistance OR weighted exercise OR resistive load OR body building OR power lifting OR training frequency OR training volume OR training load OR training intensity) AND (muscle strength OR muscular strength OR muscle morphology OR 1 RM OR repetition max* OR rep* max* OR physical strength OR force development OR max* strength)  Limiters: Peer Reviewed  English  Academic Journal | 5,478 |

Medline:

| Term | Citations |
| --- | --- |
| TITLE-ABS-KEY  (Elderly) | 5,770,782 |
| TITLE-ABS-KEY  (“young adult”) | 1,168,582 |
| TITLE-ABS-KEY  (“older adults”) | 5,771,797 |
| TITLE-ABS-KEY  (Middle-age) | 4,718,907 |
| TITLE-ABS-KEY  (Adult) | 8,285,223 |
| **(((elderly) OR (young adult)) OR (older adult) AND (medline[Filter])) OR ((middle-age) AND (adult) AND (medline[Filter]))** Filters: **MEDLINE, from 1916 - 2021**  ((("aged"[MeSH Terms] OR "aged"[All Fields] OR "elderly"[All Fields] OR "elderlies"[All Fields] OR "elderly s"[All Fields] OR "elderlys"[All Fields] OR ("young adult"[MeSH Terms] OR ("young"[All Fields] AND "adult"[All Fields]) OR "young adult"[All Fields]) OR ("aged"[MeSH Terms] OR "aged"[All Fields] OR ("older"[All Fields] AND "adult"[All Fields]) OR "older adult"[All Fields])) AND "medlinestatus medline"[All Fields]) OR (("middle aged"[MeSH Terms] OR ("middle"[All Fields] AND "aged"[All Fields]) OR "middle aged"[All Fields] OR ("middle"[All Fields] AND "age"[All Fields]) OR "middle age"[All Fields]) AND ("adult"[MeSH Terms] OR "adult"[All Fields] OR "adults"[All Fields] OR "adult s"[All Fields]) AND "medlinestatus medline"[All Fields])) AND ((medline[Filter]) AND (1916:2021[pdat]))  **Translations**  **elderly:** "aged"[MeSH Terms] OR "aged"[All Fields] OR "elderly"[All Fields] OR "elderlies"[All Fields] OR "elderly's"[All Fields] OR "elderlys"[All Fields]  **young adult:** "young adult"[MeSH Terms] OR ("young"[All Fields] AND "adult"[All Fields]) OR "young adult"[All Fields]  **older adult:** "aged"[MeSH Terms] OR "aged"[All Fields] OR ("older"[All Fields] AND "adult"[All Fields]) OR "older adult"[All Fields]  **medline[Filter]:** "medlinestatus_medline"[all]  **middle-age:** "middle aged"[MeSH Terms] OR ("middle"[All Fields] AND "aged"[All Fields]) OR "middle aged"[All Fields] OR ("middle"[All Fields] AND "age"[All Fields]) OR "middle age"[All Fields]  **adult:** "adult"[MeSH Terms] OR "adult"[All Fields] OR "adults"[All Fields] OR "adult's"[All Fields]  **medline[Filter]:** "medlinestatus_medline"[all] | 6,283,957 |
| TITLE-ABS-KEY (“resistance training”) | 24,910 |
| TITLE-ABS-KEY (“resistance exercise”) | 37,215 |
| TITLE-ABS-KEY (“weight training”) | 26,873 |
| TITLE-ABS-KEY (“weight lifting”) | 7,078 |
| TITLE-ABS-KEY (“strength training”) | 41,238 |
| TITLE-ABS-KEY (“free weight”) | 89,736 |
| TITLE-ABS-KEY (“high intensity training”) | 17,835 |
| TITLE-ABS-KEY (“progressive resistance”) | 78,970 |
| TITLE-ABS-KEY (“physical resistance”) | 114,689 |
| TITLE-ABS-KEY (“weighted exercise”) | 79,947 |
| TITLE-ABS-KEY (“resistive load”) | 21,766 |
| TITLE-ABS-KEY (“body building”) | 13,316 |
| TITLE-ABS-KEY (“power lifting”) | 1,392 |
| TITLE-ABS-KEY  (“training frequency”) | 338,921 |
| TITLE-ABS-KEY (“training volume”) | 38,821 |
| TITLE-ABS-KEY (“training load”) | 16,145 |
| TITLE-ABS-KEY (“training intensity”) | 47,553 |
| **(((((((((((((((((resistance training AND ((medline[Filter]) AND (1950:2021[pdat]))) OR (resistance exercise AND ((medline[Filter]) AND (1946:2021[pdat])))) OR (weight training AND ((medline[Filter]) AND (1950:2021[pdat])))) OR (weight lifting AND ((medline[Filter]) AND (1950:2021[pdat])))) OR (strength training AND ((medline[Filter]) AND (1946:2021[pdat])))) OR (free weight AND ((medline[Filter]) AND (1947:2021[pdat])))) OR (high intensity training AND ((medline[Filter]) AND (1955:2021[pdat])))) OR (progressive resistance AND ((medline[Filter]) AND (1946:2021[pdat])))) OR (weighted exercise AND ((medline[Filter]) AND (1950:2021[pdat])))) OR (physical resistance AND ((medline[Filter]) AND (1945:2021[pdat])))) OR (resistive load AND ((medline[Filter]) AND (1947:2021[pdat])))) OR (body building AND ((medline[Filter]) AND (1945:2021[pdat])))) OR (power lifting AND ((medline[Filter]) AND (1947:2021[pdat])))) OR (training frequency AND ((medline[Filter]) AND (1928:2021[pdat])))) OR (training frequency AND ((medline[Filter]) AND (1928:2021[pdat])))) OR (training volume AND ((medline[Filter]) AND (1947:2021[pdat])))) OR (training load AND ((medline[Filter]) AND (1948:2021[pdat])))) OR (training intensity AND ((medline[Filter]) AND (1952:2021[pdat])))** Filters: **MEDLINE, from 1928 - 2021**  ((("resistance training"[MeSH Terms] OR ("resistance"[All Fields] AND "training"[All Fields]) OR "resistance training"[All Fields]) AND ("medlinestatus medline"[All Fields] AND 1950/01/01:2021/12/31[Date - Publication])) OR (("resist"[All Fields] OR "resistance"[All Fields] OR "resistances"[All Fields] OR "resistant"[All Fields] OR "resistants"[All Fields] OR "resisted"[All Fields] OR "resistence"[All Fields] OR "resistences"[All Fields] OR "resistent"[All Fields] OR "resistibility"[All Fields] OR "resisting"[All Fields] OR "resistive"[All Fields] OR "resistively"[All Fields] OR "resistivities"[All Fields] OR "resistivity"[All Fields] OR "resists"[All Fields]) AND ("exercise"[MeSH Terms] OR "exercise"[All Fields] OR "exercises"[All Fields] OR "exercise therapy"[MeSH Terms] OR ("exercise"[All Fields] AND "therapy"[All Fields]) OR "exercise therapy"[All Fields] OR "exercising"[All Fields] OR "exercise s"[All Fields] OR "exercised"[All Fields] OR "exerciser"[All Fields] OR "exercisers"[All Fields]) AND ("medlinestatus medline"[All Fields] AND 1946/01/01:2021/12/31[Date - Publication])) OR (("weight lifting"[MeSH Terms] OR ("weight"[All Fields] AND "lifting"[All Fields]) OR "weight lifting"[All Fields] OR ("weight"[All Fields] AND "training"[All Fields]) OR "weight training"[All Fields]) AND ("medlinestatus medline"[All Fields] AND 1950/01/01:2021/12/31[Date - Publication])) OR (("weight lifting"[MeSH Terms] OR ("weight"[All Fields] AND "lifting"[All Fields]) OR "weight lifting"[All Fields]) AND ("medlinestatus medline"[All Fields] AND 1950/01/01:2021/12/31[Date - Publication])) OR (("resistance training"[MeSH Terms] OR ("resistance"[All Fields] AND "training"[All Fields]) OR "resistance training"[All Fields] OR ("strength"[All Fields] AND "training"[All Fields]) OR "strength training"[All Fields]) AND ("medlinestatus medline"[All Fields] AND 1946/01/01:2021/12/31[Date - Publication])) OR ("free"[All Fields] AND ("weight s"[All Fields] OR "weighted"[All Fields] OR "weighting"[All Fields] OR "weightings"[All Fields] OR "weights and measures"[MeSH Terms] OR ("weights"[All Fields] AND "measures"[All Fields]) OR "weights and measures"[All Fields] OR "weight"[All Fields] OR "body weight"[MeSH Terms] OR ("body"[All Fields] AND "weight"[All Fields]) OR "body weight"[All Fields] OR "weights"[All Fields]) AND ("medlinestatus medline"[All Fields] AND 1947/01/01:2021/12/31[Date - Publication])) OR ("high"[All Fields] AND ("intense"[All Fields] OR "intensely"[All Fields] OR "intensities"[All Fields] OR "intensity"[All Fields] OR "intensively"[All Fields]) AND ("education"[MeSH Subheading] OR "education"[All Fields] OR "training"[All Fields] OR "education"[MeSH Terms] OR "train"[All Fields] OR "train s"[All Fields] OR "trained"[All Fields] OR "training s"[All Fields] OR "trainings"[All Fields] OR "trains"[All Fields]) AND ("medlinestatus medline"[All Fields] AND 1955/01/01:2021/12/31[Date - Publication])) OR (("disease progression"[MeSH Terms] OR ("disease"[All Fields] AND "progression"[All Fields]) OR "disease progression"[All Fields] OR "progression"[All Fields] OR "progress"[All Fields] OR "progressed"[All Fields] OR "progresses"[All Fields] OR "progressing"[All Fields] OR "progressions"[All Fields] OR "progressive"[All Fields] OR "progressively"[All Fields] OR "progressives"[All Fields]) AND ("resist"[All Fields] OR "resistance"[All Fields] OR "resistances"[All Fields] OR "resistant"[All Fields] OR "resistants"[All Fields] OR "resisted"[All Fields] OR "resistence"[All Fields] OR "resistences"[All Fields] OR "resistent"[All Fields] OR "resistibility"[All Fields] OR "resisting"[All Fields] OR "resistive"[All Fields] OR "resistively"[All Fields] OR "resistivities"[All Fields] OR "resistivity"[All Fields] OR "resists"[All Fields]) AND ("medlinestatus medline"[All Fields] AND 1946/01/01:2021/12/31[Date - Publication])) OR (("weight s"[All Fields] OR "weighted"[All Fields] OR "weighting"[All Fields] OR "weightings"[All Fields] OR "weights and measures"[MeSH Terms] OR ("weights"[All Fields] AND "measures"[All Fields]) OR "weights and measures"[All Fields] OR "weight"[All Fields] OR "body weight"[MeSH Terms] OR ("body"[All Fields] AND "weight"[All Fields]) OR "body weight"[All Fields] OR "weights"[All Fields]) AND ("exercise"[MeSH Terms] OR "exercise"[All Fields] OR "exercises"[All Fields] OR "exercise therapy"[MeSH Terms] OR ("exercise"[All Fields] AND "therapy"[All Fields]) OR "exercise therapy"[All Fields] OR "exercising"[All Fields] OR "exercise s"[All Fields] OR "exercised"[All Fields] OR "exerciser"[All Fields] OR "exercisers"[All Fields]) AND ("medlinestatus medline"[All Fields] AND 1950/01/01:2021/12/31[Date - Publication])) OR (("physical examination"[MeSH Terms] OR ("physical"[All Fields] AND "examination"[All Fields]) OR "physical examination"[All Fields] OR "physical"[All Fields] OR "physically"[All Fields] OR "physicals"[All Fields]) AND ("resist"[All Fields] OR "resistance"[All Fields] OR "resistances"[All Fields] OR "resistant"[All Fields] OR "resistants"[All Fields] OR "resisted"[All Fields] OR "resistence"[All Fields] OR "resistences"[All Fields] OR "resistent"[All Fields] OR "resistibility"[All Fields] OR "resisting"[All Fields] OR "resistive"[All Fields] OR "resistively"[All Fields] OR "resistivities"[All Fields] OR "resistivity"[All Fields] OR "resists"[All Fields]) AND ("medlinestatus medline"[All Fields] AND 1945/01/01:2021/12/31[Date - Publication])) OR (("resist"[All Fields] OR "resistance"[All Fields] OR "resistances"[All Fields] OR "resistant"[All Fields] OR "resistants"[All Fields] OR "resisted"[All Fields] OR "resistence"[All Fields] OR "resistences"[All Fields] OR "resistent"[All Fields] OR "resistibility"[All Fields] OR "resisting"[All Fields] OR "resistive"[All Fields] OR "resistively"[All Fields] OR "resistivities"[All Fields] OR "resistivity"[All Fields] OR "resists"[All Fields]) AND "load"[All Fields] AND ("medlinestatus medline"[All Fields] AND 1947/01/01:2021/12/31[Date - Publication])) OR (("somatotypes"[MeSH Terms] OR "somatotypes"[All Fields] OR ("body"[All Fields] AND "building"[All Fields]) OR "body building"[All Fields]) AND ("medlinestatus medline"[All Fields] AND 1945/01/01:2021/12/31[Date - Publication])) OR (("power, psychological"[MeSH Terms] OR ("power"[All Fields] AND "psychological"[All Fields]) OR "psychological power"[All Fields] OR "power"[All Fields] OR "powered"[All Fields] OR "powers"[All Fields] OR "powering"[All Fields]) AND ("lifting"[MeSH Terms] OR "lifting"[All Fields] OR "lifted"[All Fields] OR "liftings"[All Fields] OR "lifts"[All Fields]) AND ("medlinestatus medline"[All Fields] AND 1947/01/01:2021/12/31[Date - Publication])) OR (("education"[MeSH Subheading] OR "education"[All Fields] OR "training"[All Fields] OR "education"[MeSH Terms] OR "train"[All Fields] OR "train s"[All Fields] OR "trained"[All Fields] OR "training s"[All Fields] OR "trainings"[All Fields] OR "trains"[All Fields]) AND ("epidemiology"[MeSH Subheading] OR "epidemiology"[All Fields] OR "frequency"[All Fields] OR "epidemiology"[MeSH Terms] OR "frequence"[All Fields] OR "frequences"[All Fields] OR "frequencies"[All Fields]) AND ("medlinestatus medline"[All Fields] AND 1928/01/01:2021/12/31[Date - Publication])) OR (("education"[MeSH Subheading] OR "education"[All Fields] OR "training"[All Fields] OR "education"[MeSH Terms] OR "train"[All Fields] OR "train s"[All Fields] OR "trained"[All Fields] OR "training s"[All Fields] OR "trainings"[All Fields] OR "trains"[All Fields]) AND ("epidemiology"[MeSH Subheading] OR "epidemiology"[All Fields] OR "frequency"[All Fields] OR "epidemiology"[MeSH Terms] OR "frequence"[All Fields] OR "frequences"[All Fields] OR "frequencies"[All Fields]) AND ("medlinestatus medline"[All Fields] AND 1928/01/01:2021/12/31[Date - Publication])) OR (("education"[MeSH Subheading] OR "education"[All Fields] OR "training"[All Fields] OR "education"[MeSH Terms] OR "train"[All Fields] OR "train s"[All Fields] OR "trained"[All Fields] OR "training s"[All Fields] OR "trainings"[All Fields] OR "trains"[All Fields]) AND ("volum"[All Fields] OR "volume"[All Fields] OR "volumes"[All Fields] OR "voluming"[All Fields]) AND ("medlinestatus medline"[All Fields] AND 1947/01/01:2021/12/31[Date - Publication])) OR (("education"[MeSH Subheading] OR "education"[All Fields] OR "training"[All Fields] OR "education"[MeSH Terms] OR "train"[All Fields] OR "train s"[All Fields] OR "trained"[All Fields] OR "training s"[All Fields] OR "trainings"[All Fields] OR "trains"[All Fields]) AND "load"[All Fields] AND ("medlinestatus medline"[All Fields] AND 1948/01/01:2021/12/31[Date - Publication])) OR (("education"[MeSH Subheading] OR "education"[All Fields] OR "training"[All Fields] OR "education"[MeSH Terms] OR "train"[All Fields] OR "train s"[All Fields] OR "trained"[All Fields] OR "training s"[All Fields] OR "trainings"[All Fields] OR "trains"[All Fields]) AND ("intense"[All Fields] OR "intensely"[All Fields] OR "intensities"[All Fields] OR "intensity"[All Fields] OR "intensively"[All Fields]) AND ("medlinestatus medline"[All Fields] AND 1952/01/01:2021/12/31[Date - Publication]))) AND ((medline[Filter]) AND (1928:2021[pdat]))  **Translations**  **resistance training:** "resistance training"[MeSH Terms] OR ("resistance"[All Fields] AND "training"[All Fields]) OR "resistance training"[All Fields]  **medline[Filter]:** "medlinestatus_medline"[all]  **resistance:** "resist"[All Fields] OR "resistance"[All Fields] OR "resistances"[All Fields] OR "resistant"[All Fields] OR "resistants"[All Fields] OR "resisted"[All Fields] OR "resistence"[All Fields] OR "resistences"[All Fields] OR "resistent"[All Fields] OR "resistibility"[All Fields] OR "resisting"[All Fields] OR "resistive"[All Fields] OR "resistively"[All Fields] OR "resistivities"[All Fields] OR "resistivity"[All Fields] OR "resists"[All Fields]  **exercise:** "exercise"[MeSH Terms] OR "exercise"[All Fields] OR "exercises"[All Fields] OR "exercise therapy"[MeSH Terms] OR ("exercise"[All Fields] AND "therapy"[All Fields]) OR "exercise therapy"[All Fields] OR "exercising"[All Fields] OR "exercise's"[All Fields] OR "exercised"[All Fields] OR "exerciser"[All Fields] OR "exercisers"[All Fields]  **medline[Filter]:** "medlinestatus_medline"[all]  **weight training:** "weight lifting"[MeSH Terms] OR ("weight"[All Fields] AND "lifting"[All Fields]) OR "weight lifting"[All Fields] OR ("weight"[All Fields] AND "training"[All Fields]) OR "weight training"[All Fields]  **medline[Filter]:** "medlinestatus_medline"[all]  **weight lifting:** "weight lifting"[MeSH Terms] OR ("weight"[All Fields] AND "lifting"[All Fields]) OR "weight lifting"[All Fields]  **medline[Filter]:** "medlinestatus_medline"[all]  **strength training:** "resistance training"[MeSH Terms] OR ("resistance"[All Fields] AND "training"[All Fields]) OR "resistance training"[All Fields] OR ("strength"[All Fields] AND "training"[All Fields]) OR "strength training"[All Fields]  **medline[Filter]:** "medlinestatus_medline"[all]  **weight:** "weight's"[All Fields] OR "weighted"[All Fields] OR "weighting"[All Fields] OR "weightings"[All Fields] OR "weights and measures"[MeSH Terms] OR ("weights"[All Fields] AND "measures"[All Fields]) OR "weights and measures"[All Fields] OR "weight"[All Fields] OR "body weight"[MeSH Terms] OR ("body"[All Fields] AND "weight"[All Fields]) OR "body weight"[All Fields] OR "weights"[All Fields]  **medline[Filter]:** "medlinestatus_medline"[all]  **intensity:** "intense"[All Fields] OR "intensely"[All Fields] OR "intensities"[All Fields] OR "intensity"[All Fields] OR "intensively"[All Fields]  **training:** "education"[Subheading] OR "education"[All Fields] OR "training"[All Fields] OR "education"[MeSH Terms] OR "train"[All Fields] OR "train's"[All Fields] OR "trained"[All Fields] OR "training's"[All Fields] OR "trainings"[All Fields] OR "trains"[All Fields]  **medline[Filter]:** "medlinestatus_medline"[all]  **progressive:** "disease progression"[MeSH Terms] OR ("disease"[All Fields] AND "progression"[All Fields]) OR "disease progression"[All Fields] OR "progression"[All Fields] OR "progress"[All Fields] OR "progressed"[All Fields] OR "progresses"[All Fields] OR "progressing"[All Fields] OR "progressions"[All Fields] OR "progressive"[All Fields] OR "progressively"[All Fields] OR "progressives"[All Fields]  **resistance:** "resist"[All Fields] OR "resistance"[All Fields] OR "resistances"[All Fields] OR "resistant"[All Fields] OR "resistants"[All Fields] OR "resisted"[All Fields] OR "resistence"[All Fields] OR "resistences"[All Fields] OR "resistent"[All Fields] OR "resistibility"[All Fields] OR "resisting"[All Fields] OR "resistive"[All Fields] OR "resistively"[All Fields] OR "resistivities"[All Fields] OR "resistivity"[All Fields] OR "resists"[All Fields]  **medline[Filter]:** "medlinestatus_medline"[all]  **weighted:** "weight's"[All Fields] OR "weighted"[All Fields] OR "weighting"[All Fields] OR "weightings"[All Fields] OR "weights and measures"[MeSH Terms] OR ("weights"[All Fields] AND "measures"[All Fields]) OR "weights and measures"[All Fields] OR "weight"[All Fields] OR "body weight"[MeSH Terms] OR ("body"[All Fields] AND "weight"[All Fields]) OR "body weight"[All Fields] OR "weights"[All Fields]  **exercise:** "exercise"[MeSH Terms] OR "exercise"[All Fields] OR "exercises"[All Fields] OR "exercise therapy"[MeSH Terms] OR ("exercise"[All Fields] AND "therapy"[All Fields]) OR "exercise therapy"[All Fields] OR "exercising"[All Fields] OR "exercise's"[All Fields] OR "exercised"[All Fields] OR "exerciser"[All Fields] OR "exercisers"[All Fields]  **medline[Filter]:** "medlinestatus_medline"[all]  **physical:** "physical examination"[MeSH Terms] OR ("physical"[All Fields] AND "examination"[All Fields]) OR "physical examination"[All Fields] OR "physical"[All Fields] OR "physically"[All Fields] OR "physicals"[All Fields]  **resistance:** "resist"[All Fields] OR "resistance"[All Fields] OR "resistances"[All Fields] OR "resistant"[All Fields] OR "resistants"[All Fields] OR "resisted"[All Fields] OR "resistence"[All Fields] OR "resistences"[All Fields] OR "resistent"[All Fields] OR "resistibility"[All Fields] OR "resisting"[All Fields] OR "resistive"[All Fields] OR "resistively"[All Fields] OR "resistivities"[All Fields] OR "resistivity"[All Fields] OR "resists"[All Fields]  **medline[Filter]:** "medlinestatus_medline"[all]  **resistive:** "resist"[All Fields] OR "resistance"[All Fields] OR "resistances"[All Fields] OR "resistant"[All Fields] OR "resistants"[All Fields] OR "resisted"[All Fields] OR "resistence"[All Fields] OR "resistences"[All Fields] OR "resistent"[All Fields] OR "resistibility"[All Fields] OR "resisting"[All Fields] OR "resistive"[All Fields] OR "resistively"[All Fields] OR "resistivities"[All Fields] OR "resistivity"[All Fields] OR "resists"[All Fields]  **medline[Filter]:** "medlinestatus_medline"[all]  **body building:** "somatotypes"[MeSH Terms] OR "somatotypes"[All Fields] OR ("body"[All Fields] AND "building"[All Fields]) OR "body building"[All Fields]  **medline[Filter]:** "medlinestatus_medline"[all]  **power:** "power, psychological"[MeSH Terms] OR ("power"[All Fields] AND "psychological"[All Fields]) OR "psychological power"[All Fields] OR "power"[All Fields] OR "powered"[All Fields] OR "powers"[All Fields] OR "powering"[All Fields]  **lifting:** "lifting"[MeSH Terms] OR "lifting"[All Fields] OR "lifted"[All Fields] OR "liftings"[All Fields] OR "lifts"[All Fields]  **medline[Filter]:** "medlinestatus_medline"[all]  **training:** "education"[Subheading] OR "education"[All Fields] OR "training"[All Fields] OR "education"[MeSH Terms] OR "train"[All Fields] OR "train's"[All Fields] OR "trained"[All Fields] OR "training's"[All Fields] OR "trainings"[All Fields] OR "trains"[All Fields]  **frequency:** "epidemiology"[Subheading] OR "epidemiology"[All Fields] OR "frequency"[All Fields] OR "epidemiology"[MeSH Terms] OR "frequence"[All Fields] OR "frequences"[All Fields] OR "frequencies"[All Fields]  **medline[Filter]:** "medlinestatus_medline"[all]  **training:** "education"[Subheading] OR "education"[All Fields] OR "training"[All Fields] OR "education"[MeSH Terms] OR "train"[All Fields] OR "train's"[All Fields] OR "trained"[All Fields] OR "training's"[All Fields] OR "trainings"[All Fields] OR "trains"[All Fields]  **frequency:** "epidemiology"[Subheading] OR "epidemiology"[All Fields] OR "frequency"[All Fields] OR "epidemiology"[MeSH Terms] OR "frequence"[All Fields] OR "frequences"[All Fields] OR "frequencies"[All Fields]  **medline[Filter]:** "medlinestatus_medline"[all]  **training:** "education"[Subheading] OR "education"[All Fields] OR "training"[All Fields] OR "education"[MeSH Terms] OR "train"[All Fields] OR "train's"[All Fields] OR "trained"[All Fields] OR "training's"[All Fields] OR "trainings"[All Fields] OR "trains"[All Fields]  **volume:** "volum"[All Fields] OR "volume"[All Fields] OR "volumes"[All Fields] OR "voluming"[All Fields]  **medline[Filter]:** "medlinestatus_medline"[all]  **training:** "education"[Subheading] OR "education"[All Fields] OR "training"[All Fields] OR "education"[MeSH Terms] OR "train"[All Fields] OR "train's"[All Fields] OR "trained"[All Fields] OR "training's"[All Fields] OR "trainings"[All Fields] OR "trains"[All Fields]  **medline[Filter]:** "medlinestatus_medline"[all]  **training:** "education"[Subheading] OR "education"[All Fields] OR "training"[All Fields] OR "education"[MeSH Terms] OR "train"[All Fields] OR "train's"[All Fields] OR "trained"[All Fields] OR "training's"[All Fields] OR "trainings"[All Fields] OR "trains"[All Fields]  **intensity:** "intense"[All Fields] OR "intensely"[All Fields] OR "intensities"[All Fields] OR "intensity"[All Fields] OR "intensively"[All Fields]  **medline[Filter]:** "medlinestatus_medline"[all] | 792,174 |
| TITLE-ABS-KEY (“muscle strength”) | 73,556 |
| TITLE-ABS-KEY (“muscular strength”) | 10,459 |
| TITLE-ABS-KEY (“muscle morphology”) | 432,690 |
| TITLE-ABS-KEY (“1 RM”) | 23,591 |
| TITLE-ABS-KEY (“repetition maxi*”) | 7,614 |
| TITLE-ABS-KEY (“repe* maxi*”) | 49,719 |
| TITLE-ABS-KEY (“physical strength”) | 95,005 |
| TITLE-ABS-KEY (“force development”) | 82,614 |
| TITLE-ABS-KEY (“max* strength”) | 40,053 |
| **((((((((muscle strength AND ((medline[Filter]) AND (1946:2021[pdat]))) OR (muscular strength AND ((medline[Filter]) AND (1945:2021[pdat])))) OR (muscle morphology AND ((medline[Filter]) AND (1945:2021[pdat])))) OR (1 RM AND ((medline[Filter]) AND (1952:2021[pdat])))) OR (repe* maxi* AND ((medline[Filter]) AND (1948:2021[pdat])))) OR (physical strength AND ((medline[Filter]) AND (1945:2021[pdat])))) OR (force development AND ((medline[Filter]) AND (1946:2021[pdat])))) OR (maxi* strength AND ((medline[Filter]) AND (1948:2021[pdat])))) OR (repetition maxi* AND ((medline[Filter]) AND (1955:2021[pdat])))** Filters: **MEDLINE, from 1945 - 2021**  ((("muscle strength"[MeSH Terms] OR ("muscle"[All Fields] AND "strength"[All Fields]) OR "muscle strength"[All Fields]) AND ("medlinestatus medline"[All Fields] AND 1946/01/01:2021/12/31[Date - Publication])) OR ("muscular"[All Fields] AND ("strength"[All Fields] OR "strengths"[All Fields]) AND ("medlinestatus medline"[All Fields] AND 1945/01/01:2021/12/31[Date - Publication])) OR (("muscle s"[All Fields] OR "muscles"[MeSH Terms] OR "muscles"[All Fields] OR "muscle"[All Fields]) AND ("anatomy and histology"[MeSH Subheading] OR ("anatomy"[All Fields] AND "histology"[All Fields]) OR "anatomy and histology"[All Fields] OR "morphology"[All Fields] OR "morphologies"[All Fields]) AND ("medlinestatus medline"[All Fields] AND 1945/01/01:2021/12/31[Date - Publication])) OR ("1"[All Fields] AND ("results math"[Journal] OR "rm"[All Fields]) AND ("medlinestatus medline"[All Fields] AND 1952/01/01:2021/12/31[Date - Publication])) OR ("repe*"[All Fields] AND "maxi*"[All Fields] AND ("medlinestatus medline"[All Fields] AND 1948/01/01:2021/12/31[Date - Publication])) OR (("physical examination"[MeSH Terms] OR ("physical"[All Fields] AND "examination"[All Fields]) OR "physical examination"[All Fields] OR "physical"[All Fields] OR "physically"[All Fields] OR "physicals"[All Fields]) AND ("strength"[All Fields] OR "strengths"[All Fields]) AND ("medlinestatus medline"[All Fields] AND 1945/01/01:2021/12/31[Date - Publication])) OR (("force"[All Fields] OR "forceful"[All Fields] OR "forcefulness"[All Fields] OR "forces"[All Fields] OR "forcing"[All Fields] OR "forcings"[All Fields]) AND ("develop"[All Fields] OR "develope"[All Fields] OR "developed"[All Fields] OR "developer"[All Fields] OR "developer s"[All Fields] OR "developers"[All Fields] OR "developing"[All Fields] OR "developments"[All Fields] OR "develops"[All Fields] OR "growth and development"[MeSH Subheading] OR ("growth"[All Fields] AND "development"[All Fields]) OR "growth and development"[All Fields] OR "development"[All Fields]) AND ("medlinestatus medline"[All Fields] AND 1946/01/01:2021/12/31[Date - Publication])) OR ("maxi*"[All Fields] AND ("strength"[All Fields] OR "strengths"[All Fields]) AND ("medlinestatus medline"[All Fields] AND 1948/01/01:2021/12/31[Date - Publication])) OR (("repetition"[All Fields] OR "repetitions"[All Fields]) AND "maxi*"[All Fields] AND ("medlinestatus medline"[All Fields] AND 1955/01/01:2021/12/31[Date - Publication]))) AND ((medline[Filter]) AND (1945:2021[pdat]))  **Translations**  **muscle strength:** "muscle strength"[MeSH Terms] OR ("muscle"[All Fields] AND "strength"[All Fields]) OR "muscle strength"[All Fields]  **medline[Filter]:** "medlinestatus_medline"[all]  **strength:** "strength"[All Fields] OR "strengths"[All Fields]  **medline[Filter]:** "medlinestatus_medline"[all]  **muscle:** "muscle's"[All Fields] OR "muscles"[MeSH Terms] OR "muscles"[All Fields] OR "muscle"[All Fields]  **morphology:** "anatomy and histology"[Subheading] OR ("anatomy"[All Fields] AND "histology"[All Fields]) OR "anatomy and histology"[All Fields] OR "morphology"[All Fields] OR "morphologies"[All Fields]  **medline[Filter]:** "medlinestatus_medline"[all]  **RM:** "Results Math"[Journal:__jid101763976] OR "rm"[All Fields]  **medline[Filter]:** "medlinestatus_medline"[all]  **medline[Filter]:** "medlinestatus_medline"[all]  **physical:** "physical examination"[MeSH Terms] OR ("physical"[All Fields] AND "examination"[All Fields]) OR "physical examination"[All Fields] OR "physical"[All Fields] OR "physically"[All Fields] OR "physicals"[All Fields]  **strength:** "strength"[All Fields] OR "strengths"[All Fields]  **medline[Filter]:** "medlinestatus_medline"[all]  **force:** "force"[All Fields] OR "forceful"[All Fields] OR "forcefulness"[All Fields] OR "forces"[All Fields] OR "forcing"[All Fields] OR "forcings"[All Fields]  **development:** "develop"[All Fields] OR "develope"[All Fields] OR "developed"[All Fields] OR "developer"[All Fields] OR "developer's"[All Fields] OR "developers"[All Fields] OR "developing"[All Fields] OR "developments"[All Fields] OR "develops"[All Fields] OR "growth and development"[Subheading] OR ("growth"[All Fields] AND "development"[All Fields]) OR "growth and development"[All Fields] OR "development"[All Fields]  **medline[Filter]:** "medlinestatus_medline"[all]  **strength:** "strength"[All Fields] OR "strengths"[All Fields]  **medline[Filter]:** "medlinestatus_medline"[all]  **repetition:** "repetition"[All Fields] OR "repetitions"[All Fields]  **medline[Filter]:** "medlinestatus_medline"[all] | 687,446 |
| **((((elderly) OR (young adult)) OR (older adult) AND (medline[Filter])) OR ((middle-age) AND (adult) AND (medline[Filter])) AND ((medline[Filter]) AND (1916:2021[pdat]))) AND ((((((((((((((((((resistance training AND ((medline[Filter]) AND (1950:2021[pdat]))) OR (resistance exercise AND ((medline[Filter]) AND (1946:2021[pdat])))) OR (weight training AND ((medline[Filter]) AND (1950:2021[pdat])))) OR (weight lifting AND ((medline[Filter]) AND (1950:2021[pdat])))) OR (strength training AND ((medline[Filter]) AND (1946:2021[pdat])))) OR (free weight AND ((medline[Filter]) AND (1947:2021[pdat])))) OR (high intensity training AND ((medline[Filter]) AND (1955:2021[pdat])))) OR (progressive resistance AND ((medline[Filter]) AND (1946:2021[pdat])))) OR (weighted exercise AND ((medline[Filter]) AND (1950:2021[pdat])))) OR (physical resistance AND ((medline[Filter]) AND (1945:2021[pdat])))) OR (resistive load AND ((medline[Filter]) AND (1947:2021[pdat])))) OR (body building AND ((medline[Filter]) AND (1945:2021[pdat])))) OR (power lifting AND ((medline[Filter]) AND (1947:2021[pdat])))) OR (training frequency AND ((medline[Filter]) AND (1928:2021[pdat])))) OR (training frequency AND ((medline[Filter]) AND (1928:2021[pdat])))) OR (training volume AND ((medline[Filter]) AND (1947:2021[pdat])))) OR (training load AND ((medline[Filter]) AND (1948:2021[pdat])))) OR (training intensity AND ((medline[Filter]) AND (1952:2021[pdat]))) AND ((medline[Filter]) AND (1928:2021[pdat])))** Filters: **MEDLINE, from 1942 - 2021**  (((("aged"[MeSH Terms] OR "aged"[All Fields] OR "elderly"[All Fields] OR "elderlies"[All Fields] OR "elderly s"[All Fields] OR "elderlys"[All Fields] OR ("young adult"[MeSH Terms] OR ("young"[All Fields] AND "adult"[All Fields]) OR "young adult"[All Fields]) OR ("aged"[MeSH Terms] OR "aged"[All Fields] OR ("older"[All Fields] AND "adult"[All Fields]) OR "older adult"[All Fields])) AND "medlinestatus medline"[All Fields]) OR (("middle aged"[MeSH Terms] OR ("middle"[All Fields] AND "aged"[All Fields]) OR "middle aged"[All Fields] OR ("middle"[All Fields] AND "age"[All Fields]) OR "middle age"[All Fields]) AND ("adult"[MeSH Terms] OR "adult"[All Fields] OR "adults"[All Fields] OR "adult s"[All Fields]) AND "medlinestatus medline"[All Fields])) AND ("medlinestatus medline"[All Fields] AND 1916/01/01:2021/12/31[Date - Publication]) AND (((("resistance training"[MeSH Terms] OR ("resistance"[All Fields] AND "training"[All Fields]) OR "resistance training"[All Fields]) AND ("medlinestatus medline"[All Fields] AND 1950/01/01:2021/12/31[Date - Publication])) OR (("resist"[All Fields] OR "resistance"[All Fields] OR "resistances"[All Fields] OR "resistant"[All Fields] OR "resistants"[All Fields] OR "resisted"[All Fields] OR "resistence"[All Fields] OR "resistences"[All Fields] OR "resistent"[All Fields] OR "resistibility"[All Fields] OR "resisting"[All Fields] OR "resistive"[All Fields] OR "resistively"[All Fields] OR "resistivities"[All Fields] OR "resistivity"[All Fields] OR "resists"[All Fields]) AND ("exercise"[MeSH Terms] OR "exercise"[All Fields] OR "exercises"[All Fields] OR "exercise therapy"[MeSH Terms] OR ("exercise"[All Fields] AND "therapy"[All Fields]) OR "exercise therapy"[All Fields] OR "exercising"[All Fields] OR "exercise s"[All Fields] OR "exercised"[All Fields] OR "exerciser"[All Fields] OR "exercisers"[All Fields]) AND ("medlinestatus medline"[All Fields] AND 1946/01/01:2021/12/31[Date - Publication])) OR (("weight lifting"[MeSH Terms] OR ("weight"[All Fields] AND "lifting"[All Fields]) OR "weight lifting"[All Fields] OR ("weight"[All Fields] AND "training"[All Fields]) OR "weight training"[All Fields]) AND ("medlinestatus medline"[All Fields] AND 1950/01/01:2021/12/31[Date - Publication])) OR (("weight lifting"[MeSH Terms] OR ("weight"[All Fields] AND "lifting"[All Fields]) OR "weight lifting"[All Fields]) AND ("medlinestatus medline"[All Fields] AND 1950/01/01:2021/12/31[Date - Publication])) OR (("resistance training"[MeSH Terms] OR ("resistance"[All Fields] AND "training"[All Fields]) OR "resistance training"[All Fields] OR ("strength"[All Fields] AND "training"[All Fields]) OR "strength training"[All Fields]) AND ("medlinestatus medline"[All Fields] AND 1946/01/01:2021/12/31[Date - Publication])) OR ("free"[All Fields] AND ("weight s"[All Fields] OR "weighted"[All Fields] OR "weighting"[All Fields] OR "weightings"[All Fields] OR "weights and measures"[MeSH Terms] OR ("weights"[All Fields] AND "measures"[All Fields]) OR "weights and measures"[All Fields] OR "weight"[All Fields] OR "body weight"[MeSH Terms] OR ("body"[All Fields] AND "weight"[All Fields]) OR "body weight"[All Fields] OR "weights"[All Fields]) AND ("medlinestatus medline"[All Fields] AND 1947/01/01:2021/12/31[Date - Publication])) OR ("high"[All Fields] AND ("intense"[All Fields] OR "intensely"[All Fields] OR "intensities"[All Fields] OR "intensity"[All Fields] OR "intensively"[All Fields]) AND ("education"[MeSH Subheading] OR "education"[All Fields] OR "training"[All Fields] OR "education"[MeSH Terms] OR "train"[All Fields] OR "train s"[All Fields] OR "trained"[All Fields] OR "training s"[All Fields] OR "trainings"[All Fields] OR "trains"[All Fields]) AND ("medlinestatus medline"[All Fields] AND 1955/01/01:2021/12/31[Date - Publication])) OR (("disease progression"[MeSH Terms] OR ("disease"[All Fields] AND "progression"[All Fields]) OR "disease progression"[All Fields] OR "progression"[All Fields] OR "progress"[All Fields] OR "progressed"[All Fields] OR "progresses"[All Fields] OR "progressing"[All Fields] OR "progressions"[All Fields] OR "progressive"[All Fields] OR "progressively"[All Fields] OR "progressives"[All Fields]) AND ("resist"[All Fields] OR "resistance"[All Fields] OR "resistances"[All Fields] OR "resistant"[All Fields] OR "resistants"[All Fields] OR "resisted"[All Fields] OR "resistence"[All Fields] OR "resistences"[All Fields] OR "resistent"[All Fields] OR "resistibility"[All Fields] OR "resisting"[All Fields] OR "resistive"[All Fields] OR "resistively"[All Fields] OR "resistivities"[All Fields] OR "resistivity"[All Fields] OR "resists"[All Fields]) AND ("medlinestatus medline"[All Fields] AND 1946/01/01:2021/12/31[Date - Publication])) OR (("weight s"[All Fields] OR "weighted"[All Fields] OR "weighting"[All Fields] OR "weightings"[All Fields] OR "weights and measures"[MeSH Terms] OR ("weights"[All Fields] AND "measures"[All Fields]) OR "weights and measures"[All Fields] OR "weight"[All Fields] OR "body weight"[MeSH Terms] OR ("body"[All Fields] AND "weight"[All Fields]) OR "body weight"[All Fields] OR "weights"[All Fields]) AND ("exercise"[MeSH Terms] OR "exercise"[All Fields] OR "exercises"[All Fields] OR "exercise therapy"[MeSH Terms] OR ("exercise"[All Fields] AND "therapy"[All Fields]) OR "exercise therapy"[All Fields] OR "exercising"[All Fields] OR "exercise s"[All Fields] OR "exercised"[All Fields] OR "exerciser"[All Fields] OR "exercisers"[All Fields]) AND ("medlinestatus medline"[All Fields] AND 1950/01/01:2021/12/31[Date - Publication])) OR (("physical examination"[MeSH Terms] OR ("physical"[All Fields] AND "examination"[All Fields]) OR "physical examination"[All Fields] OR "physical"[All Fields] OR "physically"[All Fields] OR "physicals"[All Fields]) AND ("resist"[All Fields] OR "resistance"[All Fields] OR "resistances"[All Fields] OR "resistant"[All Fields] OR "resistants"[All Fields] OR "resisted"[All Fields] OR "resistence"[All Fields] OR "resistences"[All Fields] OR "resistent"[All Fields] OR "resistibility"[All Fields] OR "resisting"[All Fields] OR "resistive"[All Fields] OR "resistively"[All Fields] OR "resistivities"[All Fields] OR "resistivity"[All Fields] OR "resists"[All Fields]) AND ("medlinestatus medline"[All Fields] AND 1945/01/01:2021/12/31[Date - Publication])) OR (("resist"[All Fields] OR "resistance"[All Fields] OR "resistances"[All Fields] OR "resistant"[All Fields] OR "resistants"[All Fields] OR "resisted"[All Fields] OR "resistence"[All Fields] OR "resistences"[All Fields] OR "resistent"[All Fields] OR "resistibility"[All Fields] OR "resisting"[All Fields] OR "resistive"[All Fields] OR "resistively"[All Fields] OR "resistivities"[All Fields] OR "resistivity"[All Fields] OR "resists"[All Fields]) AND "load"[All Fields] AND ("medlinestatus medline"[All Fields] AND 1947/01/01:2021/12/31[Date - Publication])) OR (("somatotypes"[MeSH Terms] OR "somatotypes"[All Fields] OR ("body"[All Fields] AND "building"[All Fields]) OR "body building"[All Fields]) AND ("medlinestatus medline"[All Fields] AND 1945/01/01:2021/12/31[Date - Publication])) OR (("power, psychological"[MeSH Terms] OR ("power"[All Fields] AND "psychological"[All Fields]) OR "psychological power"[All Fields] OR "power"[All Fields] OR "powered"[All Fields] OR "powers"[All Fields] OR "powering"[All Fields]) AND ("lifting"[MeSH Terms] OR "lifting"[All Fields] OR "lifted"[All Fields] OR "liftings"[All Fields] OR "lifts"[All Fields]) AND ("medlinestatus medline"[All Fields] AND 1947/01/01:2021/12/31[Date - Publication])) OR (("education"[MeSH Subheading] OR "education"[All Fields] OR "training"[All Fields] OR "education"[MeSH Terms] OR "train"[All Fields] OR "train s"[All Fields] OR "trained"[All Fields] OR "training s"[All Fields] OR "trainings"[All Fields] OR "trains"[All Fields]) AND ("epidemiology"[MeSH Subheading] OR "epidemiology"[All Fields] OR "frequency"[All Fields] OR "epidemiology"[MeSH Terms] OR "frequence"[All Fields] OR "frequences"[All Fields] OR "frequencies"[All Fields]) AND ("medlinestatus medline"[All Fields] AND 1928/01/01:2021/12/31[Date - Publication])) OR (("education"[MeSH Subheading] OR "education"[All Fields] OR "training"[All Fields] OR "education"[MeSH Terms] OR "train"[All Fields] OR "train s"[All Fields] OR "trained"[All Fields] OR "training s"[All Fields] OR "trainings"[All Fields] OR "trains"[All Fields]) AND ("epidemiology"[MeSH Subheading] OR "epidemiology"[All Fields] OR "frequency"[All Fields] OR "epidemiology"[MeSH Terms] OR "frequence"[All Fields] OR "frequences"[All Fields] OR "frequencies"[All Fields]) AND ("medlinestatus medline"[All Fields] AND 1928/01/01:2021/12/31[Date - Publication])) OR (("education"[MeSH Subheading] OR "education"[All Fields] OR "training"[All Fields] OR "education"[MeSH Terms] OR "train"[All Fields] OR "train s"[All Fields] OR "trained"[All Fields] OR "training s"[All Fields] OR "trainings"[All Fields] OR "trains"[All Fields]) AND ("volum"[All Fields] OR "volume"[All Fields] OR "volumes"[All Fields] OR "voluming"[All Fields]) AND ("medlinestatus medline"[All Fields] AND 1947/01/01:2021/12/31[Date - Publication])) OR (("education"[MeSH Subheading] OR "education"[All Fields] OR "training"[All Fields] OR "education"[MeSH Terms] OR "train"[All Fields] OR "train s"[All Fields] OR "trained"[All Fields] OR "training s"[All Fields] OR "trainings"[All Fields] OR "trains"[All Fields]) AND "load"[All Fields] AND ("medlinestatus medline"[All Fields] AND 1948/01/01:2021/12/31[Date - Publication])) OR (("education"[MeSH Subheading] OR "education"[All Fields] OR "training"[All Fields] OR "education"[MeSH Terms] OR "train"[All Fields] OR "train s"[All Fields] OR "trained"[All Fields] OR "training s"[All Fields] OR "trainings"[All Fields] OR "trains"[All Fields]) AND ("intense"[All Fields] OR "intensely"[All Fields] OR "intensities"[All Fields] OR "intensity"[All Fields] OR "intensively"[All Fields]) AND ("medlinestatus medline"[All Fields] AND 1952/01/01:2021/12/31[Date - Publication]))) AND ("medlinestatus medline"[All Fields] AND 1928/01/01:2021/12/31[Date - Publication]))) AND ((medline[Filter]) AND (1942:2021[pdat]))  **Translations**  **elderly:** "aged"[MeSH Terms] OR "aged"[All Fields] OR "elderly"[All Fields] OR "elderlies"[All Fields] OR "elderly's"[All Fields] OR "elderlys"[All Fields]  **young adult:** "young adult"[MeSH Terms] OR ("young"[All Fields] AND "adult"[All Fields]) OR "young adult"[All Fields]  **older adult:** "aged"[MeSH Terms] OR "aged"[All Fields] OR ("older"[All Fields] AND "adult"[All Fields]) OR "older adult"[All Fields]  **medline[Filter]:** "medlinestatus_medline"[all]  **middle-age:** "middle aged"[MeSH Terms] OR ("middle"[All Fields] AND "aged"[All Fields]) OR "middle aged"[All Fields] OR ("middle"[All Fields] AND "age"[All Fields]) OR "middle age"[All Fields]  **adult:** "adult"[MeSH Terms] OR "adult"[All Fields] OR "adults"[All Fields] OR "adult's"[All Fields]  **medline[Filter]:** "medlinestatus_medline"[all]  **medline[Filter]:** "medlinestatus_medline"[all]  **resistance training:** "resistance training"[MeSH Terms] OR ("resistance"[All Fields] AND "training"[All Fields]) OR "resistance training"[All Fields]  **medline[Filter]:** "medlinestatus_medline"[all]  **resistance:** "resist"[All Fields] OR "resistance"[All Fields] OR "resistances"[All Fields] OR "resistant"[All Fields] OR "resistants"[All Fields] OR "resisted"[All Fields] OR "resistence"[All Fields] OR "resistences"[All Fields] OR "resistent"[All Fields] OR "resistibility"[All Fields] OR "resisting"[All Fields] OR "resistive"[All Fields] OR "resistively"[All Fields] OR "resistivities"[All Fields] OR "resistivity"[All Fields] OR "resists"[All Fields]  **exercise:** "exercise"[MeSH Terms] OR "exercise"[All Fields] OR "exercises"[All Fields] OR "exercise therapy"[MeSH Terms] OR ("exercise"[All Fields] AND "therapy"[All Fields]) OR "exercise therapy"[All Fields] OR "exercising"[All Fields] OR "exercise's"[All Fields] OR "exercised"[All Fields] OR "exerciser"[All Fields] OR "exercisers"[All Fields]  **medline[Filter]:** "medlinestatus_medline"[all]  **weight training:** "weight lifting"[MeSH Terms] OR ("weight"[All Fields] AND "lifting"[All Fields]) OR "weight lifting"[All Fields] OR ("weight"[All Fields] AND "training"[All Fields]) OR "weight training"[All Fields]  **medline[Filter]:** "medlinestatus_medline"[all]  **weight lifting:** "weight lifting"[MeSH Terms] OR ("weight"[All Fields] AND "lifting"[All Fields]) OR "weight lifting"[All Fields]  **medline[Filter]:** "medlinestatus_medline"[all]  **strength training:** "resistance training"[MeSH Terms] OR ("resistance"[All Fields] AND "training"[All Fields]) OR "resistance training"[All Fields] OR ("strength"[All Fields] AND "training"[All Fields]) OR "strength training"[All Fields]  **medline[Filter]:** "medlinestatus_medline"[all]  **weight:** "weight's"[All Fields] OR "weighted"[All Fields] OR "weighting"[All Fields] OR "weightings"[All Fields] OR "weights and measures"[MeSH Terms] OR ("weights"[All Fields] AND "measures"[All Fields]) OR "weights and measures"[All Fields] OR "weight"[All Fields] OR "body weight"[MeSH Terms] OR ("body"[All Fields] AND "weight"[All Fields]) OR "body weight"[All Fields] OR "weights"[All Fields]  **medline[Filter]:** "medlinestatus_medline"[all]  **intensity:** "intense"[All Fields] OR "intensely"[All Fields] OR "intensities"[All Fields] OR "intensity"[All Fields] OR "intensively"[All Fields]  **training:** "education"[Subheading] OR "education"[All Fields] OR "training"[All Fields] OR "education"[MeSH Terms] OR "train"[All Fields] OR "train's"[All Fields] OR "trained"[All Fields] OR "training's"[All Fields] OR "trainings"[All Fields] OR "trains"[All Fields]  **medline[Filter]:** "medlinestatus_medline"[all]  **progressive:** "disease progression"[MeSH Terms] OR ("disease"[All Fields] AND "progression"[All Fields]) OR "disease progression"[All Fields] OR "progression"[All Fields] OR "progress"[All Fields] OR "progressed"[All Fields] OR "progresses"[All Fields] OR "progressing"[All Fields] OR "progressions"[All Fields] OR "progressive"[All Fields] OR "progressively"[All Fields] OR "progressives"[All Fields]  **resistance:** "resist"[All Fields] OR "resistance"[All Fields] OR "resistances"[All Fields] OR "resistant"[All Fields] OR "resistants"[All Fields] OR "resisted"[All Fields] OR "resistence"[All Fields] OR "resistences"[All Fields] OR "resistent"[All Fields] OR "resistibility"[All Fields] OR "resisting"[All Fields] OR "resistive"[All Fields] OR "resistively"[All Fields] OR "resistivities"[All Fields] OR "resistivity"[All Fields] OR "resists"[All Fields]  **medline[Filter]:** "medlinestatus_medline"[all]  **weighted:** "weight's"[All Fields] OR "weighted"[All Fields] OR "weighting"[All Fields] OR "weightings"[All Fields] OR "weights and measures"[MeSH Terms] OR ("weights"[All Fields] AND "measures"[All Fields]) OR "weights and measures"[All Fields] OR "weight"[All Fields] OR "body weight"[MeSH Terms] OR ("body"[All Fields] AND "weight"[All Fields]) OR "body weight"[All Fields] OR "weights"[All Fields]  **exercise:** "exercise"[MeSH Terms] OR "exercise"[All Fields] OR "exercises"[All Fields] OR "exercise therapy"[MeSH Terms] OR ("exercise"[All Fields] AND "therapy"[All Fields]) OR "exercise therapy"[All Fields] OR "exercising"[All Fields] OR "exercise's"[All Fields] OR "exercised"[All Fields] OR "exerciser"[All Fields] OR "exercisers"[All Fields]  **medline[Filter]:** "medlinestatus_medline"[all]  **physical:** "physical examination"[MeSH Terms] OR ("physical"[All Fields] AND "examination"[All Fields]) OR "physical examination"[All Fields] OR "physical"[All Fields] OR "physically"[All Fields] OR "physicals"[All Fields]  **resistance:** "resist"[All Fields] OR "resistance"[All Fields] OR "resistances"[All Fields] OR "resistant"[All Fields] OR "resistants"[All Fields] OR "resisted"[All Fields] OR "resistence"[All Fields] OR "resistences"[All Fields] OR "resistent"[All Fields] OR "resistibility"[All Fields] OR "resisting"[All Fields] OR "resistive"[All Fields] OR "resistively"[All Fields] OR "resistivities"[All Fields] OR "resistivity"[All Fields] OR "resists"[All Fields]  **medline[Filter]:** "medlinestatus_medline"[all]  **resistive:** "resist"[All Fields] OR "resistance"[All Fields] OR "resistances"[All Fields] OR "resistant"[All Fields] OR "resistants"[All Fields] OR "resisted"[All Fields] OR "resistence"[All Fields] OR "resistences"[All Fields] OR "resistent"[All Fields] OR "resistibility"[All Fields] OR "resisting"[All Fields] OR "resistive"[All Fields] OR "resistively"[All Fields] OR "resistivities"[All Fields] OR "resistivity"[All Fields] OR "resists"[All Fields]  **medline[Filter]:** "medlinestatus_medline"[all]  **body building:** "somatotypes"[MeSH Terms] OR "somatotypes"[All Fields] OR ("body"[All Fields] AND "building"[All Fields]) OR "body building"[All Fields]  **medline[Filter]:** "medlinestatus_medline"[all]  **power:** "power, psychological"[MeSH Terms] OR ("power"[All Fields] AND "psychological"[All Fields]) OR "psychological power"[All Fields] OR "power"[All Fields] OR "powered"[All Fields] OR "powers"[All Fields] OR "powering"[All Fields]  **lifting:** "lifting"[MeSH Terms] OR "lifting"[All Fields] OR "lifted"[All Fields] OR "liftings"[All Fields] OR "lifts"[All Fields]  **medline[Filter]:** "medlinestatus_medline"[all]  **training:** "education"[Subheading] OR "education"[All Fields] OR "training"[All Fields] OR "education"[MeSH Terms] OR "train"[All Fields] OR "train's"[All Fields] OR "trained"[All Fields] OR "training's"[All Fields] OR "trainings"[All Fields] OR "trains"[All Fields]  **frequency:** "epidemiology"[Subheading] OR "epidemiology"[All Fields] OR "frequency"[All Fields] OR "epidemiology"[MeSH Terms] OR "frequence"[All Fields] OR "frequences"[All Fields] OR "frequencies"[All Fields]  **medline[Filter]:** "medlinestatus_medline"[all]  **training:** "education"[Subheading] OR "education"[All Fields] OR "training"[All Fields] OR "education"[MeSH Terms] OR "train"[All Fields] OR "train's"[All Fields] OR "trained"[All Fields] OR "training's"[All Fields] OR "trainings"[All Fields] OR "trains"[All Fields]  **frequency:** "epidemiology"[Subheading] OR "epidemiology"[All Fields] OR "frequency"[All Fields] OR "epidemiology"[MeSH Terms] OR "frequence"[All Fields] OR "frequences"[All Fields] OR "frequencies"[All Fields]  **medline[Filter]:** "medlinestatus_medline"[all]  **training:** "education"[Subheading] OR "education"[All Fields] OR "training"[All Fields] OR "education"[MeSH Terms] OR "train"[All Fields] OR "train's"[All Fields] OR "trained"[All Fields] OR "training's"[All Fields] OR "trainings"[All Fields] OR "trains"[All Fields]  **volume:** "volum"[All Fields] OR "volume"[All Fields] OR "volumes"[All Fields] OR "voluming"[All Fields]  **medline[Filter]:** "medlinestatus_medline"[all]  **training:** "education"[Subheading] OR "education"[All Fields] OR "training"[All Fields] OR "education"[MeSH Terms] OR "train"[All Fields] OR "train's"[All Fields] OR "trained"[All Fields] OR "training's"[All Fields] OR "trainings"[All Fields] OR "trains"[All Fields]  **medline[Filter]:** "medlinestatus_medline"[all]  **training:** "education"[Subheading] OR "education"[All Fields] OR "training"[All Fields] OR "education"[MeSH Terms] OR "train"[All Fields] OR "train's"[All Fields] OR "trained"[All Fields] OR "training's"[All Fields] OR "trainings"[All Fields] OR "trains"[All Fields]  **intensity:** "intense"[All Fields] OR "intensely"[All Fields] OR "intensities"[All Fields] OR "intensity"[All Fields] OR "intensively"[All Fields]  **medline[Filter]:** "medlinestatus_medline"[all]  **medline[Filter]:** "medlinestatus_medline"[all] | 315,427 |
| **((((((((((muscle strength AND ((medline[Filter]) AND (1946:2021[pdat]))) OR (muscular strength AND ((medline[Filter]) AND (1945:2021[pdat])))) OR (muscle morphology AND ((medline[Filter]) AND (1945:2021[pdat])))) OR (1 RM AND ((medline[Filter]) AND (1952:2021[pdat])))) OR (repe* maxi* AND ((medline[Filter]) AND (1948:2021[pdat])))) OR (physical strength AND ((medline[Filter]) AND (1945:2021[pdat])))) OR (force development AND ((medline[Filter]) AND (1946:2021[pdat])))) OR (maxi* strength AND ((medline[Filter]) AND (1948:2021[pdat])))) OR (repetition maxi* AND ((medline[Filter]) AND (1955:2021[pdat]))) AND ((medline[Filter]) AND (1945:2021[pdat]))) AND ((((((((((((((((((resistance training AND ((medline[Filter]) AND (1950:2021[pdat]))) OR (resistance exercise AND ((medline[Filter]) AND (1946:2021[pdat])))) OR (weight training AND ((medline[Filter]) AND (1950:2021[pdat])))) OR (weight lifting AND ((medline[Filter]) AND (1950:2021[pdat])))) OR (strength training AND ((medline[Filter]) AND (1946:2021[pdat])))) OR (free weight AND ((medline[Filter]) AND (1947:2021[pdat])))) OR (high intensity training AND ((medline[Filter]) AND (1955:2021[pdat])))) OR (progressive resistance AND ((medline[Filter]) AND (1946:2021[pdat])))) OR (weighted exercise AND ((medline[Filter]) AND (1950:2021[pdat])))) OR (physical resistance AND ((medline[Filter]) AND (1945:2021[pdat])))) OR (resistive load AND ((medline[Filter]) AND (1947:2021[pdat])))) OR (body building AND ((medline[Filter]) AND (1945:2021[pdat])))) OR (power lifting AND ((medline[Filter]) AND (1947:2021[pdat])))) OR (training frequency AND ((medline[Filter]) AND (1928:2021[pdat])))) OR (training frequency AND ((medline[Filter]) AND (1928:2021[pdat])))) OR (training volume AND ((medline[Filter]) AND (1947:2021[pdat])))) OR (training load AND ((medline[Filter]) AND (1948:2021[pdat])))) OR (training intensity AND ((medline[Filter]) AND (1952:2021[pdat]))) AND ((medline[Filter]) AND (1928:2021[pdat])))) AND ((((elderly) OR (young adult)) OR (older adult) AND (medline[Filter])) OR ((middle-age) AND (adult) AND (medline[Filter])) AND ((medline[Filter]) AND (1916:2021[pdat])))** Filters: **MEDLINE, from 1965 - 2021**  (((("muscle strength"[MeSH Terms] OR ("muscle"[All Fields] AND "strength"[All Fields]) OR "muscle strength"[All Fields]) AND ("medlinestatus medline"[All Fields] AND 1946/01/01:2021/12/31[Date - Publication])) OR ("muscular"[All Fields] AND ("strength"[All Fields] OR "strengths"[All Fields]) AND ("medlinestatus medline"[All Fields] AND 1945/01/01:2021/12/31[Date - Publication])) OR (("muscle s"[All Fields] OR "muscles"[MeSH Terms] OR "muscles"[All Fields] OR "muscle"[All Fields]) AND ("anatomy and histology"[MeSH Subheading] OR ("anatomy"[All Fields] AND "histology"[All Fields]) OR "anatomy and histology"[All Fields] OR "morphology"[All Fields] OR "morphologies"[All Fields]) AND ("medlinestatus medline"[All Fields] AND 1945/01/01:2021/12/31[Date - Publication])) OR ("1"[All Fields] AND ("results math"[Journal] OR "rm"[All Fields]) AND ("medlinestatus medline"[All Fields] AND 1952/01/01:2021/12/31[Date - Publication])) OR ("repe*"[All Fields] AND "maxi*"[All Fields] AND ("medlinestatus medline"[All Fields] AND 1948/01/01:2021/12/31[Date - Publication])) OR (("physical examination"[MeSH Terms] OR ("physical"[All Fields] AND "examination"[All Fields]) OR "physical examination"[All Fields] OR "physical"[All Fields] OR "physically"[All Fields] OR "physicals"[All Fields]) AND ("strength"[All Fields] OR "strengths"[All Fields]) AND ("medlinestatus medline"[All Fields] AND 1945/01/01:2021/12/31[Date - Publication])) OR (("force"[All Fields] OR "forceful"[All Fields] OR "forcefulness"[All Fields] OR "forces"[All Fields] OR "forcing"[All Fields] OR "forcings"[All Fields]) AND ("develop"[All Fields] OR "develope"[All Fields] OR "developed"[All Fields] OR "developer"[All Fields] OR "developer s"[All Fields] OR "developers"[All Fields] OR "developing"[All Fields] OR "developments"[All Fields] OR "develops"[All Fields] OR "growth and development"[MeSH Subheading] OR ("growth"[All Fields] AND "development"[All Fields]) OR "growth and development"[All Fields] OR "development"[All Fields]) AND ("medlinestatus medline"[All Fields] AND 1946/01/01:2021/12/31[Date - Publication])) OR ("maxi*"[All Fields] AND ("strength"[All Fields] OR "strengths"[All Fields]) AND ("medlinestatus medline"[All Fields] AND 1948/01/01:2021/12/31[Date - Publication])) OR (("repetition"[All Fields] OR "repetitions"[All Fields]) AND "maxi*"[All Fields] AND ("medlinestatus medline"[All Fields] AND 1955/01/01:2021/12/31[Date - Publication]))) AND ("medlinestatus medline"[All Fields] AND 1945/01/01:2021/12/31[Date - Publication]) AND (((("resistance training"[MeSH Terms] OR ("resistance"[All Fields] AND "training"[All Fields]) OR "resistance training"[All Fields]) AND ("medlinestatus medline"[All Fields] AND 1950/01/01:2021/12/31[Date - Publication])) OR (("resist"[All Fields] OR "resistance"[All Fields] OR "resistances"[All Fields] OR "resistant"[All Fields] OR "resistants"[All Fields] OR "resisted"[All Fields] OR "resistence"[All Fields] OR "resistences"[All Fields] OR "resistent"[All Fields] OR "resistibility"[All Fields] OR "resisting"[All Fields] OR "resistive"[All Fields] OR "resistively"[All Fields] OR "resistivities"[All Fields] OR "resistivity"[All Fields] OR "resists"[All Fields]) AND ("exercise"[MeSH Terms] OR "exercise"[All Fields] OR "exercises"[All Fields] OR "exercise therapy"[MeSH Terms] OR ("exercise"[All Fields] AND "therapy"[All Fields]) OR "exercise therapy"[All Fields] OR "exercising"[All Fields] OR "exercise s"[All Fields] OR "exercised"[All Fields] OR "exerciser"[All Fields] OR "exercisers"[All Fields]) AND ("medlinestatus medline"[All Fields] AND 1946/01/01:2021/12/31[Date - Publication])) OR (("weight lifting"[MeSH Terms] OR ("weight"[All Fields] AND "lifting"[All Fields]) OR "weight lifting"[All Fields] OR ("weight"[All Fields] AND "training"[All Fields]) OR "weight training"[All Fields]) AND ("medlinestatus medline"[All Fields] AND 1950/01/01:2021/12/31[Date - Publication])) OR (("weight lifting"[MeSH Terms] OR ("weight"[All Fields] AND "lifting"[All Fields]) OR "weight lifting"[All Fields]) AND ("medlinestatus medline"[All Fields] AND 1950/01/01:2021/12/31[Date - Publication])) OR (("resistance training"[MeSH Terms] OR ("resistance"[All Fields] AND "training"[All Fields]) OR "resistance training"[All Fields] OR ("strength"[All Fields] AND "training"[All Fields]) OR "strength training"[All Fields]) AND ("medlinestatus medline"[All Fields] AND 1946/01/01:2021/12/31[Date - Publication])) OR ("free"[All Fields] AND ("weight s"[All Fields] OR "weighted"[All Fields] OR "weighting"[All Fields] OR "weightings"[All Fields] OR "weights and measures"[MeSH Terms] OR ("weights"[All Fields] AND "measures"[All Fields]) OR "weights and measures"[All Fields] OR "weight"[All Fields] OR "body weight"[MeSH Terms] OR ("body"[All Fields] AND "weight"[All Fields]) OR "body weight"[All Fields] OR "weights"[All Fields]) AND ("medlinestatus medline"[All Fields] AND 1947/01/01:2021/12/31[Date - Publication])) OR ("high"[All Fields] AND ("intense"[All Fields] OR "intensely"[All Fields] OR "intensities"[All Fields] OR "intensity"[All Fields] OR "intensively"[All Fields]) AND ("education"[MeSH Subheading] OR "education"[All Fields] OR "training"[All Fields] OR "education"[MeSH Terms] OR "train"[All Fields] OR "train s"[All Fields] OR "trained"[All Fields] OR "training s"[All Fields] OR "trainings"[All Fields] OR "trains"[All Fields]) AND ("medlinestatus medline"[All Fields] AND 1955/01/01:2021/12/31[Date - Publication])) OR (("disease progression"[MeSH Terms] OR ("disease"[All Fields] AND "progression"[All Fields]) OR "disease progression"[All Fields] OR "progression"[All Fields] OR "progress"[All Fields] OR "progressed"[All Fields] OR "progresses"[All Fields] OR "progressing"[All Fields] OR "progressions"[All Fields] OR "progressive"[All Fields] OR "progressively"[All Fields] OR "progressives"[All Fields]) AND ("resist"[All Fields] OR "resistance"[All Fields] OR "resistances"[All Fields] OR "resistant"[All Fields] OR "resistants"[All Fields] OR "resisted"[All Fields] OR "resistence"[All Fields] OR "resistences"[All Fields] OR "resistent"[All Fields] OR "resistibility"[All Fields] OR "resisting"[All Fields] OR "resistive"[All Fields] OR "resistively"[All Fields] OR "resistivities"[All Fields] OR "resistivity"[All Fields] OR "resists"[All Fields]) AND ("medlinestatus medline"[All Fields] AND 1946/01/01:2021/12/31[Date - Publication])) OR (("weight s"[All Fields] OR "weighted"[All Fields] OR "weighting"[All Fields] OR "weightings"[All Fields] OR "weights and measures"[MeSH Terms] OR ("weights"[All Fields] AND "measures"[All Fields]) OR "weights and measures"[All Fields] OR "weight"[All Fields] OR "body weight"[MeSH Terms] OR ("body"[All Fields] AND "weight"[All Fields]) OR "body weight"[All Fields] OR "weights"[All Fields]) AND ("exercise"[MeSH Terms] OR "exercise"[All Fields] OR "exercises"[All Fields] OR "exercise therapy"[MeSH Terms] OR ("exercise"[All Fields] AND "therapy"[All Fields]) OR "exercise therapy"[All Fields] OR "exercising"[All Fields] OR "exercise s"[All Fields] OR "exercised"[All Fields] OR "exerciser"[All Fields] OR "exercisers"[All Fields]) AND ("medlinestatus medline"[All Fields] AND 1950/01/01:2021/12/31[Date - Publication])) OR (("physical examination"[MeSH Terms] OR ("physical"[All Fields] AND "examination"[All Fields]) OR "physical examination"[All Fields] OR "physical"[All Fields] OR "physically"[All Fields] OR "physicals"[All Fields]) AND ("resist"[All Fields] OR "resistance"[All Fields] OR "resistances"[All Fields] OR "resistant"[All Fields] OR "resistants"[All Fields] OR "resisted"[All Fields] OR "resistence"[All Fields] OR "resistences"[All Fields] OR "resistent"[All Fields] OR "resistibility"[All Fields] OR "resisting"[All Fields] OR "resistive"[All Fields] OR "resistively"[All Fields] OR "resistivities"[All Fields] OR "resistivity"[All Fields] OR "resists"[All Fields]) AND ("medlinestatus medline"[All Fields] AND 1945/01/01:2021/12/31[Date - Publication])) OR (("resist"[All Fields] OR "resistance"[All Fields] OR "resistances"[All Fields] OR "resistant"[All Fields] OR "resistants"[All Fields] OR "resisted"[All Fields] OR "resistence"[All Fields] OR "resistences"[All Fields] OR "resistent"[All Fields] OR "resistibility"[All Fields] OR "resisting"[All Fields] OR "resistive"[All Fields] OR "resistively"[All Fields] OR "resistivities"[All Fields] OR "resistivity"[All Fields] OR "resists"[All Fields]) AND "load"[All Fields] AND ("medlinestatus medline"[All Fields] AND 1947/01/01:2021/12/31[Date - Publication])) OR (("somatotypes"[MeSH Terms] OR "somatotypes"[All Fields] OR ("body"[All Fields] AND "building"[All Fields]) OR "body building"[All Fields]) AND ("medlinestatus medline"[All Fields] AND 1945/01/01:2021/12/31[Date - Publication])) OR (("power, psychological"[MeSH Terms] OR ("power"[All Fields] AND "psychological"[All Fields]) OR "psychological power"[All Fields] OR "power"[All Fields] OR "powered"[All Fields] OR "powers"[All Fields] OR "powering"[All Fields]) AND ("lifting"[MeSH Terms] OR "lifting"[All Fields] OR "lifted"[All Fields] OR "liftings"[All Fields] OR "lifts"[All Fields]) AND ("medlinestatus medline"[All Fields] AND 1947/01/01:2021/12/31[Date - Publication])) OR (("education"[MeSH Subheading] OR "education"[All Fields] OR "training"[All Fields] OR "education"[MeSH Terms] OR "train"[All Fields] OR "train s"[All Fields] OR "trained"[All Fields] OR "training s"[All Fields] OR "trainings"[All Fields] OR "trains"[All Fields]) AND ("epidemiology"[MeSH Subheading] OR "epidemiology"[All Fields] OR "frequency"[All Fields] OR "epidemiology"[MeSH Terms] OR "frequence"[All Fields] OR "frequences"[All Fields] OR "frequencies"[All Fields]) AND ("medlinestatus medline"[All Fields] AND 1928/01/01:2021/12/31[Date - Publication])) OR (("education"[MeSH Subheading] OR "education"[All Fields] OR "training"[All Fields] OR "education"[MeSH Terms] OR "train"[All Fields] OR "train s"[All Fields] OR "trained"[All Fields] OR "training s"[All Fields] OR "trainings"[All Fields] OR "trains"[All Fields]) AND ("epidemiology"[MeSH Subheading] OR "epidemiology"[All Fields] OR "frequency"[All Fields] OR "epidemiology"[MeSH Terms] OR "frequence"[All Fields] OR "frequences"[All Fields] OR "frequencies"[All Fields]) AND ("medlinestatus medline"[All Fields] AND 1928/01/01:2021/12/31[Date - Publication])) OR (("education"[MeSH Subheading] OR "education"[All Fields] OR "training"[All Fields] OR "education"[MeSH Terms] OR "train"[All Fields] OR "train s"[All Fields] OR "trained"[All Fields] OR "training s"[All Fields] OR "trainings"[All Fields] OR "trains"[All Fields]) AND ("volum"[All Fields] OR "volume"[All Fields] OR "volumes"[All Fields] OR "voluming"[All Fields]) AND ("medlinestatus medline"[All Fields] AND 1947/01/01:2021/12/31[Date - Publication])) OR (("education"[MeSH Subheading] OR "education"[All Fields] OR "training"[All Fields] OR "education"[MeSH Terms] OR "train"[All Fields] OR "train s"[All Fields] OR "trained"[All Fields] OR "training s"[All Fields] OR "trainings"[All Fields] OR "trains"[All Fields]) AND "load"[All Fields] AND ("medlinestatus medline"[All Fields] AND 1948/01/01:2021/12/31[Date - Publication])) OR (("education"[MeSH Subheading] OR "education"[All Fields] OR "training"[All Fields] OR "education"[MeSH Terms] OR "train"[All Fields] OR "train s"[All Fields] OR "trained"[All Fields] OR "training s"[All Fields] OR "trainings"[All Fields] OR "trains"[All Fields]) AND ("intense"[All Fields] OR "intensely"[All Fields] OR "intensities"[All Fields] OR "intensity"[All Fields] OR "intensively"[All Fields]) AND ("medlinestatus medline"[All Fields] AND 1952/01/01:2021/12/31[Date - Publication]))) AND ("medlinestatus medline"[All Fields] AND 1928/01/01:2021/12/31[Date - Publication])) AND (((("aged"[MeSH Terms] OR "aged"[All Fields] OR "elderly"[All Fields] OR "elderlies"[All Fields] OR "elderly s"[All Fields] OR "elderlys"[All Fields] OR ("young adult"[MeSH Terms] OR ("young"[All Fields] AND "adult"[All Fields]) OR "young adult"[All Fields]) OR ("aged"[MeSH Terms] OR "aged"[All Fields] OR ("older"[All Fields] AND "adult"[All Fields]) OR "older adult"[All Fields])) AND "medlinestatus medline"[All Fields]) OR (("middle aged"[MeSH Terms] OR ("middle"[All Fields] AND "aged"[All Fields]) OR "middle aged"[All Fields] OR ("middle"[All Fields] AND "age"[All Fields]) OR "middle age"[All Fields]) AND ("adult"[MeSH Terms] OR "adult"[All Fields] OR "adults"[All Fields] OR "adult s"[All Fields]) AND "medlinestatus medline"[All Fields])) AND ("medlinestatus medline"[All Fields] AND 1916/01/01:2021/12/31[Date - Publication]))) AND ((medline[Filter]) AND (1965:2021[pdat]))  **Translations**  **muscle strength:** "muscle strength"[MeSH Terms] OR ("muscle"[All Fields] AND "strength"[All Fields]) OR "muscle strength"[All Fields]  **medline[Filter]:** "medlinestatus_medline"[all]  **strength:** "strength"[All Fields] OR "strengths"[All Fields]  **medline[Filter]:** "medlinestatus_medline"[all]  **muscle:** "muscle's"[All Fields] OR "muscles"[MeSH Terms] OR "muscles"[All Fields] OR "muscle"[All Fields]  **morphology:** "anatomy and histology"[Subheading] OR ("anatomy"[All Fields] AND "histology"[All Fields]) OR "anatomy and histology"[All Fields] OR "morphology"[All Fields] OR "morphologies"[All Fields]  **medline[Filter]:** "medlinestatus_medline"[all]  **RM:** "Results Math"[Journal:__jid101763976] OR "rm"[All Fields]  **medline[Filter]:** "medlinestatus_medline"[all]  **medline[Filter]:** "medlinestatus_medline"[all]  **physical:** "physical examination"[MeSH Terms] OR ("physical"[All Fields] AND "examination"[All Fields]) OR "physical examination"[All Fields] OR "physical"[All Fields] OR "physically"[All Fields] OR "physicals"[All Fields]  **strength:** "strength"[All Fields] OR "strengths"[All Fields]  **medline[Filter]:** "medlinestatus_medline"[all]  **force:** "force"[All Fields] OR "forceful"[All Fields] OR "forcefulness"[All Fields] OR "forces"[All Fields] OR "forcing"[All Fields] OR "forcings"[All Fields]  **development:** "develop"[All Fields] OR "develope"[All Fields] OR "developed"[All Fields] OR "developer"[All Fields] OR "developer's"[All Fields] OR "developers"[All Fields] OR "developing"[All Fields] OR "developments"[All Fields] OR "develops"[All Fields] OR "growth and development"[Subheading] OR ("growth"[All Fields] AND "development"[All Fields]) OR "growth and development"[All Fields] OR "development"[All Fields]  **medline[Filter]:** "medlinestatus_medline"[all]  **strength:** "strength"[All Fields] OR "strengths"[All Fields]  **medline[Filter]:** "medlinestatus_medline"[all]  **repetition:** "repetition"[All Fields] OR "repetitions"[All Fields]  **medline[Filter]:** "medlinestatus_medline"[all]  **medline[Filter]:** "medlinestatus_medline"[all]  **resistance training:** "resistance training"[MeSH Terms] OR ("resistance"[All Fields] AND "training"[All Fields]) OR "resistance training"[All Fields]  **medline[Filter]:** "medlinestatus_medline"[all]  **resistance:** "resist"[All Fields] OR "resistance"[All Fields] OR "resistances"[All Fields] OR "resistant"[All Fields] OR "resistants"[All Fields] OR "resisted"[All Fields] OR "resistence"[All Fields] OR "resistences"[All Fields] OR "resistent"[All Fields] OR "resistibility"[All Fields] OR "resisting"[All Fields] OR "resistive"[All Fields] OR "resistively"[All Fields] OR "resistivities"[All Fields] OR "resistivity"[All Fields] OR "resists"[All Fields]  **exercise:** "exercise"[MeSH Terms] OR "exercise"[All Fields] OR "exercises"[All Fields] OR "exercise therapy"[MeSH Terms] OR ("exercise"[All Fields] AND "therapy"[All Fields]) OR "exercise therapy"[All Fields] OR "exercising"[All Fields] OR "exercise's"[All Fields] OR "exercised"[All Fields] OR "exerciser"[All Fields] OR "exercisers"[All Fields]  **medline[Filter]:** "medlinestatus_medline"[all]  **weight training:** "weight lifting"[MeSH Terms] OR ("weight"[All Fields] AND "lifting"[All Fields]) OR "weight lifting"[All Fields] OR ("weight"[All Fields] AND "training"[All Fields]) OR "weight training"[All Fields]  **medline[Filter]:** "medlinestatus_medline"[all]  **weight lifting:** "weight lifting"[MeSH Terms] OR ("weight"[All Fields] AND "lifting"[All Fields]) OR "weight lifting"[All Fields]  **medline[Filter]:** "medlinestatus_medline"[all]  **strength training:** "resistance training"[MeSH Terms] OR ("resistance"[All Fields] AND "training"[All Fields]) OR "resistance training"[All Fields] OR ("strength"[All Fields] AND "training"[All Fields]) OR "strength training"[All Fields]  **medline[Filter]:** "medlinestatus_medline"[all]  **weight:** "weight's"[All Fields] OR "weighted"[All Fields] OR "weighting"[All Fields] OR "weightings"[All Fields] OR "weights and measures"[MeSH Terms] OR ("weights"[All Fields] AND "measures"[All Fields]) OR "weights and measures"[All Fields] OR "weight"[All Fields] OR "body weight"[MeSH Terms] OR ("body"[All Fields] AND "weight"[All Fields]) OR "body weight"[All Fields] OR "weights"[All Fields]  **medline[Filter]:** "medlinestatus_medline"[all]  **intensity:** "intense"[All Fields] OR "intensely"[All Fields] OR "intensities"[All Fields] OR "intensity"[All Fields] OR "intensively"[All Fields]  **training:** "education"[Subheading] OR "education"[All Fields] OR "training"[All Fields] OR "education"[MeSH Terms] OR "train"[All Fields] OR "train's"[All Fields] OR "trained"[All Fields] OR "training's"[All Fields] OR "trainings"[All Fields] OR "trains"[All Fields]  **medline[Filter]:** "medlinestatus_medline"[all]  **progressive:** "disease progression"[MeSH Terms] OR ("disease"[All Fields] AND "progression"[All Fields]) OR "disease progression"[All Fields] OR "progression"[All Fields] OR "progress"[All Fields] OR "progressed"[All Fields] OR "progresses"[All Fields] OR "progressing"[All Fields] OR "progressions"[All Fields] OR "progressive"[All Fields] OR "progressively"[All Fields] OR "progressives"[All Fields]  **resistance:** "resist"[All Fields] OR "resistance"[All Fields] OR "resistances"[All Fields] OR "resistant"[All Fields] OR "resistants"[All Fields] OR "resisted"[All Fields] OR "resistence"[All Fields] OR "resistences"[All Fields] OR "resistent"[All Fields] OR "resistibility"[All Fields] OR "resisting"[All Fields] OR "resistive"[All Fields] OR "resistively"[All Fields] OR "resistivities"[All Fields] OR "resistivity"[All Fields] OR "resists"[All Fields]  **medline[Filter]:** "medlinestatus_medline"[all]  **weighted:** "weight's"[All Fields] OR "weighted"[All Fields] OR "weighting"[All Fields] OR "weightings"[All Fields] OR "weights and measures"[MeSH Terms] OR ("weights"[All Fields] AND "measures"[All Fields]) OR "weights and measures"[All Fields] OR "weight"[All Fields] OR "body weight"[MeSH Terms] OR ("body"[All Fields] AND "weight"[All Fields]) OR "body weight"[All Fields] OR "weights"[All Fields]  **exercise:** "exercise"[MeSH Terms] OR "exercise"[All Fields] OR "exercises"[All Fields] OR "exercise therapy"[MeSH Terms] OR ("exercise"[All Fields] AND "therapy"[All Fields]) OR "exercise therapy"[All Fields] OR "exercising"[All Fields] OR "exercise's"[All Fields] OR "exercised"[All Fields] OR "exerciser"[All Fields] OR "exercisers"[All Fields]  **medline[Filter]:** "medlinestatus_medline"[all]  **physical:** "physical examination"[MeSH Terms] OR ("physical"[All Fields] AND "examination"[All Fields]) OR "physical examination"[All Fields] OR "physical"[All Fields] OR "physically"[All Fields] OR "physicals"[All Fields]  **resistance:** "resist"[All Fields] OR "resistance"[All Fields] OR "resistances"[All Fields] OR "resistant"[All Fields] OR "resistants"[All Fields] OR "resisted"[All Fields] OR "resistence"[All Fields] OR "resistences"[All Fields] OR "resistent"[All Fields] OR "resistibility"[All Fields] OR "resisting"[All Fields] OR "resistive"[All Fields] OR "resistively"[All Fields] OR "resistivities"[All Fields] OR "resistivity"[All Fields] OR "resists"[All Fields]  **medline[Filter]:** "medlinestatus_medline"[all]  **resistive:** "resist"[All Fields] OR "resistance"[All Fields] OR "resistances"[All Fields] OR "resistant"[All Fields] OR "resistants"[All Fields] OR "resisted"[All Fields] OR "resistence"[All Fields] OR "resistences"[All Fields] OR "resistent"[All Fields] OR "resistibility"[All Fields] OR "resisting"[All Fields] OR "resistive"[All Fields] OR "resistively"[All Fields] OR "resistivities"[All Fields] OR "resistivity"[All Fields] OR "resists"[All Fields]  **medline[Filter]:** "medlinestatus_medline"[all]  **body building:** "somatotypes"[MeSH Terms] OR "somatotypes"[All Fields] OR ("body"[All Fields] AND "building"[All Fields]) OR "body building"[All Fields]  **medline[Filter]:** "medlinestatus_medline"[all]  **power:** "power, psychological"[MeSH Terms] OR ("power"[All Fields] AND "psychological"[All Fields]) OR "psychological power"[All Fields] OR "power"[All Fields] OR "powered"[All Fields] OR "powers"[All Fields] OR "powering"[All Fields]  **lifting:** "lifting"[MeSH Terms] OR "lifting"[All Fields] OR "lifted"[All Fields] OR "liftings"[All Fields] OR "lifts"[All Fields]  **medline[Filter]:** "medlinestatus_medline"[all]  **training:** "education"[Subheading] OR "education"[All Fields] OR "training"[All Fields] OR "education"[MeSH Terms] OR "train"[All Fields] OR "train's"[All Fields] OR "trained"[All Fields] OR "training's"[All Fields] OR "trainings"[All Fields] OR "trains"[All Fields]  **frequency:** "epidemiology"[Subheading] OR "epidemiology"[All Fields] OR "frequency"[All Fields] OR "epidemiology"[MeSH Terms] OR "frequence"[All Fields] OR "frequences"[All Fields] OR "frequencies"[All Fields]  **medline[Filter]:** "medlinestatus_medline"[all]  **training:** "education"[Subheading] OR "education"[All Fields] OR "training"[All Fields] OR "education"[MeSH Terms] OR "train"[All Fields] OR "train's"[All Fields] OR "trained"[All Fields] OR "training's"[All Fields] OR "trainings"[All Fields] OR "trains"[All Fields]  **frequency:** "epidemiology"[Subheading] OR "epidemiology"[All Fields] OR "frequency"[All Fields] OR "epidemiology"[MeSH Terms] OR "frequence"[All Fields] OR "frequences"[All Fields] OR "frequencies"[All Fields]  **medline[Filter]:** "medlinestatus_medline"[all]  **training:** "education"[Subheading] OR "education"[All Fields] OR "training"[All Fields] OR "education"[MeSH Terms] OR "train"[All Fields] OR "train's"[All Fields] OR "trained"[All Fields] OR "training's"[All Fields] OR "trainings"[All Fields] OR "trains"[All Fields]  **volume:** "volum"[All Fields] OR "volume"[All Fields] OR "volumes"[All Fields] OR "voluming"[All Fields]  **medline[Filter]:** "medlinestatus_medline"[all]  **training:** "education"[Subheading] OR "education"[All Fields] OR "training"[All Fields] OR "education"[MeSH Terms] OR "train"[All Fields] OR "train's"[All Fields] OR "trained"[All Fields] OR "training's"[All Fields] OR "trainings"[All Fields] OR "trains"[All Fields]  **medline[Filter]:** "medlinestatus_medline"[all]  **training:** "education"[Subheading] OR "education"[All Fields] OR "training"[All Fields] OR "education"[MeSH Terms] OR "train"[All Fields] OR "train's"[All Fields] OR "trained"[All Fields] OR "training's"[All Fields] OR "trainings"[All Fields] OR "trains"[All Fields]  **intensity:** "intense"[All Fields] OR "intensely"[All Fields] OR "intensities"[All Fields] OR "intensity"[All Fields] OR "intensively"[All Fields]  **medline[Filter]:** "medlinestatus_medline"[all]  **medline[Filter]:** "medlinestatus_medline"[all]  **elderly:** "aged"[MeSH Terms] OR "aged"[All Fields] OR "elderly"[All Fields] OR "elderlies"[All Fields] OR "elderly's"[All Fields] OR "elderlys"[All Fields]  **young adult:** "young adult"[MeSH Terms] OR ("young"[All Fields] AND "adult"[All Fields]) OR "young adult"[All Fields]  **older adult:** "aged"[MeSH Terms] OR "aged"[All Fields] OR ("older"[All Fields] AND "adult"[All Fields]) OR "older adult"[All Fields]  **medline[Filter]:** "medlinestatus_medline"[all]  **middle-age:** "middle aged"[MeSH Terms] OR ("middle"[All Fields] AND "aged"[All Fields]) OR "middle aged"[All Fields] OR ("middle"[All Fields] AND "age"[All Fields]) OR "middle age"[All Fields]  **adult:** "adult"[MeSH Terms] OR "adult"[All Fields] OR "adults"[All Fields] OR "adult's"[All Fields]  **medline[Filter]:** "medlinestatus_medline"[all]  **medline[Filter]:** "medlinestatus_medline"[all] | 26,419 |
| **((((elderly) OR (young adult)) OR (older adult)) OR ((middle-age) AND (adult))) AND ((((((((((((((((resistance training) OR (resistance exercise)) OR (weight training)) OR (weight lifting)) OR (strength training)) OR (free weight)) OR (high intensity training)) OR (progressive resistance)) OR (physical resistance)) OR (weighted exercise)) OR (resistive load)) OR (body building)) OR (power lifting)) OR (training frequency)) OR (training volume)) OR (training load)) OR (training intensity) AND (((((((((muscle strength) OR (muscular strength)) OR (muscle morphology)) OR (1 RM)) OR (repe* maxi*)) OR (physical strength)) OR (force development)) OR (maxi* strength)) OR (repetition maxi*) AND ((medline[Filter])))** Filters: **Randomized Controlled Trial, English, MEDLINE, from 1959 - 2021**  (((("aged"[MeSH Terms] OR "aged"[All Fields] OR "elderly"[All Fields] OR "elderlies"[All Fields] OR "elderly s"[All Fields] OR "elderlys"[All Fields] OR ("young adult"[MeSH Terms] OR ("young"[All Fields] AND "adult"[All Fields]) OR "young adult"[All Fields]) OR ("aged"[MeSH Terms] OR "aged"[All Fields] OR ("older"[All Fields] AND "adult"[All Fields]) OR "older adult"[All Fields]) OR (("middle aged"[MeSH Terms] OR ("middle"[All Fields] AND "aged"[All Fields]) OR "middle aged"[All Fields] OR ("middle"[All Fields] AND "age"[All Fields]) OR "middle age"[All Fields]) AND ("adult"[MeSH Terms] OR "adult"[All Fields] OR "adults"[All Fields] OR "adult s"[All Fields]))) AND ("resistance training"[MeSH Terms] OR ("resistance"[All Fields] AND "training"[All Fields]) OR "resistance training"[All Fields] OR (("resist"[All Fields] OR "resistance"[All Fields] OR "resistances"[All Fields] OR "resistant"[All Fields] OR "resistants"[All Fields] OR "resisted"[All Fields] OR "resistence"[All Fields] OR "resistences"[All Fields] OR "resistent"[All Fields] OR "resistibility"[All Fields] OR "resisting"[All Fields] OR "resistive"[All Fields] OR "resistively"[All Fields] OR "resistivities"[All Fields] OR "resistivity"[All Fields] OR "resists"[All Fields]) AND ("exercise"[MeSH Terms] OR "exercise"[All Fields] OR "exercises"[All Fields] OR "exercise therapy"[MeSH Terms] OR ("exercise"[All Fields] AND "therapy"[All Fields]) OR "exercise therapy"[All Fields] OR "exercising"[All Fields] OR "exercise s"[All Fields] OR "exercised"[All Fields] OR "exerciser"[All Fields] OR "exercisers"[All Fields])) OR ("weight lifting"[MeSH Terms] OR ("weight"[All Fields] AND "lifting"[All Fields]) OR "weight lifting"[All Fields] OR ("weight"[All Fields] AND "training"[All Fields]) OR "weight training"[All Fields]) OR ("weight lifting"[MeSH Terms] OR ("weight"[All Fields] AND "lifting"[All Fields]) OR "weight lifting"[All Fields]) OR ("resistance training"[MeSH Terms] OR ("resistance"[All Fields] AND "training"[All Fields]) OR "resistance training"[All Fields] OR ("strength"[All Fields] AND "training"[All Fields]) OR "strength training"[All Fields]) OR ("free"[All Fields] AND ("weight s"[All Fields] OR "weighted"[All Fields] OR "weighting"[All Fields] OR "weightings"[All Fields] OR "weights and measures"[MeSH Terms] OR ("weights"[All Fields] AND "measures"[All Fields]) OR "weights and measures"[All Fields] OR "weight"[All Fields] OR "body weight"[MeSH Terms] OR ("body"[All Fields] AND "weight"[All Fields]) OR "body weight"[All Fields] OR "weights"[All Fields])) OR ("high"[All Fields] AND ("intense"[All Fields] OR "intensely"[All Fields] OR "intensities"[All Fields] OR "intensity"[All Fields] OR "intensively"[All Fields]) AND ("education"[MeSH Subheading] OR "education"[All Fields] OR "training"[All Fields] OR "education"[MeSH Terms] OR "train"[All Fields] OR "train s"[All Fields] OR "trained"[All Fields] OR "training s"[All Fields] OR "trainings"[All Fields] OR "trains"[All Fields])) OR (("disease progression"[MeSH Terms] OR ("disease"[All Fields] AND "progression"[All Fields]) OR "disease progression"[All Fields] OR "progression"[All Fields] OR "progress"[All Fields] OR "progressed"[All Fields] OR "progresses"[All Fields] OR "progressing"[All Fields] OR "progressions"[All Fields] OR "progressive"[All Fields] OR "progressively"[All Fields] OR "progressives"[All Fields]) AND ("resist"[All Fields] OR "resistance"[All Fields] OR "resistances"[All Fields] OR "resistant"[All Fields] OR "resistants"[All Fields] OR "resisted"[All Fields] OR "resistence"[All Fields] OR "resistences"[All Fields] OR "resistent"[All Fields] OR "resistibility"[All Fields] OR "resisting"[All Fields] OR "resistive"[All Fields] OR "resistively"[All Fields] OR "resistivities"[All Fields] OR "resistivity"[All Fields] OR "resists"[All Fields])) OR (("physical examination"[MeSH Terms] OR ("physical"[All Fields] AND "examination"[All Fields]) OR "physical examination"[All Fields] OR "physical"[All Fields] OR "physically"[All Fields] OR "physicals"[All Fields]) AND ("resist"[All Fields] OR "resistance"[All Fields] OR "resistances"[All Fields] OR "resistant"[All Fields] OR "resistants"[All Fields] OR "resisted"[All Fields] OR "resistence"[All Fields] OR "resistences"[All Fields] OR "resistent"[All Fields] OR "resistibility"[All Fields] OR "resisting"[All Fields] OR "resistive"[All Fields] OR "resistively"[All Fields] OR "resistivities"[All Fields] OR "resistivity"[All Fields] OR "resists"[All Fields])) OR (("weight s"[All Fields] OR "weighted"[All Fields] OR "weighting"[All Fields] OR "weightings"[All Fields] OR "weights and measures"[MeSH Terms] OR ("weights"[All Fields] AND "measures"[All Fields]) OR "weights and measures"[All Fields] OR "weight"[All Fields] OR "body weight"[MeSH Terms] OR ("body"[All Fields] AND "weight"[All Fields]) OR "body weight"[All Fields] OR "weights"[All Fields]) AND ("exercise"[MeSH Terms] OR "exercise"[All Fields] OR "exercises"[All Fields] OR "exercise therapy"[MeSH Terms] OR ("exercise"[All Fields] AND "therapy"[All Fields]) OR "exercise therapy"[All Fields] OR "exercising"[All Fields] OR "exercise s"[All Fields] OR "exercised"[All Fields] OR "exerciser"[All Fields] OR "exercisers"[All Fields])) OR (("resist"[All Fields] OR "resistance"[All Fields] OR "resistances"[All Fields] OR "resistant"[All Fields] OR "resistants"[All Fields] OR "resisted"[All Fields] OR "resistence"[All Fields] OR "resistences"[All Fields] OR "resistent"[All Fields] OR "resistibility"[All Fields] OR "resisting"[All Fields] OR "resistive"[All Fields] OR "resistively"[All Fields] OR "resistivities"[All Fields] OR "resistivity"[All Fields] OR "resists"[All Fields]) AND "load"[All Fields]) OR ("somatotypes"[MeSH Terms] OR "somatotypes"[All Fields] OR ("body"[All Fields] AND "building"[All Fields]) OR "body building"[All Fields]) OR (("power, psychological"[MeSH Terms] OR ("power"[All Fields] AND "psychological"[All Fields]) OR "psychological power"[All Fields] OR "power"[All Fields] OR "powered"[All Fields] OR "powers"[All Fields] OR "powering"[All Fields]) AND ("lifting"[MeSH Terms] OR "lifting"[All Fields] OR "lifted"[All Fields] OR "liftings"[All Fields] OR "lifts"[All Fields])) OR (("education"[MeSH Subheading] OR "education"[All Fields] OR "training"[All Fields] OR "education"[MeSH Terms] OR "train"[All Fields] OR "train s"[All Fields] OR "trained"[All Fields] OR "training s"[All Fields] OR "trainings"[All Fields] OR "trains"[All Fields]) AND ("epidemiology"[MeSH Subheading] OR "epidemiology"[All Fields] OR "frequency"[All Fields] OR "epidemiology"[MeSH Terms] OR "frequence"[All Fields] OR "frequences"[All Fields] OR "frequencies"[All Fields])) OR (("education"[MeSH Subheading] OR "education"[All Fields] OR "training"[All Fields] OR "education"[MeSH Terms] OR "train"[All Fields] OR "train s"[All Fields] OR "trained"[All Fields] OR "training s"[All Fields] OR "trainings"[All Fields] OR "trains"[All Fields]) AND ("volum"[All Fields] OR "volume"[All Fields] OR "volumes"[All Fields] OR "voluming"[All Fields])) OR (("education"[MeSH Subheading] OR "education"[All Fields] OR "training"[All Fields] OR "education"[MeSH Terms] OR "train"[All Fields] OR "train s"[All Fields] OR "trained"[All Fields] OR "training s"[All Fields] OR "trainings"[All Fields] OR "trains"[All Fields]) AND "load"[All Fields]))) OR (("education"[MeSH Subheading] OR "education"[All Fields] OR "training"[All Fields] OR "education"[MeSH Terms] OR "train"[All Fields] OR "train s"[All Fields] OR "trained"[All Fields] OR "training s"[All Fields] OR "trainings"[All Fields] OR "trains"[All Fields]) AND ("intense"[All Fields] OR "intensely"[All Fields] OR "intensities"[All Fields] OR "intensity"[All Fields] OR "intensively"[All Fields]))) AND (("muscle strength"[MeSH Terms] OR ("muscle"[All Fields] AND "strength"[All Fields]) OR "muscle strength"[All Fields] OR ("muscular"[All Fields] AND ("strength"[All Fields] OR "strengths"[All Fields])) OR (("muscle s"[All Fields] OR "muscles"[MeSH Terms] OR "muscles"[All Fields] OR "muscle"[All Fields]) AND ("anatomy and histology"[MeSH Subheading] OR ("anatomy"[All Fields] AND "histology"[All Fields]) OR "anatomy and histology"[All Fields] OR "morphology"[All Fields] OR "morphologies"[All Fields])) OR ("1"[All Fields] AND ("results math"[Journal] OR "rm"[All Fields])) OR ("repe*"[All Fields] AND "maxi*"[All Fields]) OR (("physical examination"[MeSH Terms] OR ("physical"[All Fields] AND "examination"[All Fields]) OR "physical examination"[All Fields] OR "physical"[All Fields] OR "physically"[All Fields] OR "physicals"[All Fields]) AND ("strength"[All Fields] OR "strengths"[All Fields])) OR (("force"[All Fields] OR "forceful"[All Fields] OR "forcefulness"[All Fields] OR "forces"[All Fields] OR "forcing"[All Fields] OR "forcings"[All Fields]) AND ("develop"[All Fields] OR "develope"[All Fields] OR "developed"[All Fields] OR "developer"[All Fields] OR "developer s"[All Fields] OR "developers"[All Fields] OR "developing"[All Fields] OR "developments"[All Fields] OR "develops"[All Fields] OR "growth and development"[MeSH Subheading] OR ("growth"[All Fields] AND "development"[All Fields]) OR "growth and development"[All Fields] OR "development"[All Fields])) OR ("maxi*"[All Fields] AND ("strength"[All Fields] OR "strengths"[All Fields])) OR (("repetition"[All Fields] OR "repetitions"[All Fields]) AND "maxi*"[All Fields])) AND "medlinestatus medline"[All Fields])) AND ((medline[Filter]) AND (randomizedcontrolledtrial[Filter]) AND (english[Filter]) AND (1959:2021[pdat]))  **Translations**  **elderly:** "aged"[MeSH Terms] OR "aged"[All Fields] OR "elderly"[All Fields] OR "elderlies"[All Fields] OR "elderly's"[All Fields] OR "elderlys"[All Fields]  **young adult:** "young adult"[MeSH Terms] OR ("young"[All Fields] AND "adult"[All Fields]) OR "young adult"[All Fields]  **older adult:** "aged"[MeSH Terms] OR "aged"[All Fields] OR ("older"[All Fields] AND "adult"[All Fields]) OR "older adult"[All Fields]  **middle-age:** "middle aged"[MeSH Terms] OR ("middle"[All Fields] AND "aged"[All Fields]) OR "middle aged"[All Fields] OR ("middle"[All Fields] AND "age"[All Fields]) OR "middle age"[All Fields]  **adult:** "adult"[MeSH Terms] OR "adult"[All Fields] OR "adults"[All Fields] OR "adult's"[All Fields]  **resistance training:** "resistance training"[MeSH Terms] OR ("resistance"[All Fields] AND "training"[All Fields]) OR "resistance training"[All Fields]  **resistance:** "resist"[All Fields] OR "resistance"[All Fields] OR "resistances"[All Fields] OR "resistant"[All Fields] OR "resistants"[All Fields] OR "resisted"[All Fields] OR "resistence"[All Fields] OR "resistences"[All Fields] OR "resistent"[All Fields] OR "resistibility"[All Fields] OR "resisting"[All Fields] OR "resistive"[All Fields] OR "resistively"[All Fields] OR "resistivities"[All Fields] OR "resistivity"[All Fields] OR "resists"[All Fields]  **exercise:** "exercise"[MeSH Terms] OR "exercise"[All Fields] OR "exercises"[All Fields] OR "exercise therapy"[MeSH Terms] OR ("exercise"[All Fields] AND "therapy"[All Fields]) OR "exercise therapy"[All Fields] OR "exercising"[All Fields] OR "exercise's"[All Fields] OR "exercised"[All Fields] OR "exerciser"[All Fields] OR "exercisers"[All Fields]  **weight training:** "weight lifting"[MeSH Terms] OR ("weight"[All Fields] AND "lifting"[All Fields]) OR "weight lifting"[All Fields] OR ("weight"[All Fields] AND "training"[All Fields]) OR "weight training"[All Fields]  **weight lifting:** "weight lifting"[MeSH Terms] OR ("weight"[All Fields] AND "lifting"[All Fields]) OR "weight lifting"[All Fields]  **strength training:** "resistance training"[MeSH Terms] OR ("resistance"[All Fields] AND "training"[All Fields]) OR "resistance training"[All Fields] OR ("strength"[All Fields] AND "training"[All Fields]) OR "strength training"[All Fields]  **weight:** "weight's"[All Fields] OR "weighted"[All Fields] OR "weighting"[All Fields] OR "weightings"[All Fields] OR "weights and measures"[MeSH Terms] OR ("weights"[All Fields] AND "measures"[All Fields]) OR "weights and measures"[All Fields] OR "weight"[All Fields] OR "body weight"[MeSH Terms] OR ("body"[All Fields] AND "weight"[All Fields]) OR "body weight"[All Fields] OR "weights"[All Fields]  **intensity:** "intense"[All Fields] OR "intensely"[All Fields] OR "intensities"[All Fields] OR "intensity"[All Fields] OR "intensively"[All Fields]  **training:** "education"[Subheading] OR "education"[All Fields] OR "training"[All Fields] OR "education"[MeSH Terms] OR "train"[All Fields] OR "train's"[All Fields] OR "trained"[All Fields] OR "training's"[All Fields] OR "trainings"[All Fields] OR "trains"[All Fields]  **progressive:** "disease progression"[MeSH Terms] OR ("disease"[All Fields] AND "progression"[All Fields]) OR "disease progression"[All Fields] OR "progression"[All Fields] OR "progress"[All Fields] OR "progressed"[All Fields] OR "progresses"[All Fields] OR "progressing"[All Fields] OR "progressions"[All Fields] OR "progressive"[All Fields] OR "progressively"[All Fields] OR "progressives"[All Fields]  **resistance:** "resist"[All Fields] OR "resistance"[All Fields] OR "resistances"[All Fields] OR "resistant"[All Fields] OR "resistants"[All Fields] OR "resisted"[All Fields] OR "resistence"[All Fields] OR "resistences"[All Fields] OR "resistent"[All Fields] OR "resistibility"[All Fields] OR "resisting"[All Fields] OR "resistive"[All Fields] OR "resistively"[All Fields] OR "resistivities"[All Fields] OR "resistivity"[All Fields] OR "resists"[All Fields]  **physical:** "physical examination"[MeSH Terms] OR ("physical"[All Fields] AND "examination"[All Fields]) OR "physical examination"[All Fields] OR "physical"[All Fields] OR "physically"[All Fields] OR "physicals"[All Fields]  **resistance:** "resist"[All Fields] OR "resistance"[All Fields] OR "resistances"[All Fields] OR "resistant"[All Fields] OR "resistants"[All Fields] OR "resisted"[All Fields] OR "resistence"[All Fields] OR "resistences"[All Fields] OR "resistent"[All Fields] OR "resistibility"[All Fields] OR "resisting"[All Fields] OR "resistive"[All Fields] OR "resistively"[All Fields] OR "resistivities"[All Fields] OR "resistivity"[All Fields] OR "resists"[All Fields]  **weighted:** "weight's"[All Fields] OR "weighted"[All Fields] OR "weighting"[All Fields] OR "weightings"[All Fields] OR "weights and measures"[MeSH Terms] OR ("weights"[All Fields] AND "measures"[All Fields]) OR "weights and measures"[All Fields] OR "weight"[All Fields] OR "body weight"[MeSH Terms] OR ("body"[All Fields] AND "weight"[All Fields]) OR "body weight"[All Fields] OR "weights"[All Fields]  **exercise:** "exercise"[MeSH Terms] OR "exercise"[All Fields] OR "exercises"[All Fields] OR "exercise therapy"[MeSH Terms] OR ("exercise"[All Fields] AND "therapy"[All Fields]) OR "exercise therapy"[All Fields] OR "exercising"[All Fields] OR "exercise's"[All Fields] OR "exercised"[All Fields] OR "exerciser"[All Fields] OR "exercisers"[All Fields]  **resistive:** "resist"[All Fields] OR "resistance"[All Fields] OR "resistances"[All Fields] OR "resistant"[All Fields] OR "resistants"[All Fields] OR "resisted"[All Fields] OR "resistence"[All Fields] OR "resistences"[All Fields] OR "resistent"[All Fields] OR "resistibility"[All Fields] OR "resisting"[All Fields] OR "resistive"[All Fields] OR "resistively"[All Fields] OR "resistivities"[All Fields] OR "resistivity"[All Fields] OR "resists"[All Fields]  **body building:** "somatotypes"[MeSH Terms] OR "somatotypes"[All Fields] OR ("body"[All Fields] AND "building"[All Fields]) OR "body building"[All Fields]  **power:** "power, psychological"[MeSH Terms] OR ("power"[All Fields] AND "psychological"[All Fields]) OR "psychological power"[All Fields] OR "power"[All Fields] OR "powered"[All Fields] OR "powers"[All Fields] OR "powering"[All Fields]  **lifting:** "lifting"[MeSH Terms] OR "lifting"[All Fields] OR "lifted"[All Fields] OR "liftings"[All Fields] OR "lifts"[All Fields]  **training:** "education"[Subheading] OR "education"[All Fields] OR "training"[All Fields] OR "education"[MeSH Terms] OR "train"[All Fields] OR "train's"[All Fields] OR "trained"[All Fields] OR "training's"[All Fields] OR "trainings"[All Fields] OR "trains"[All Fields]  **frequency:** "epidemiology"[Subheading] OR "epidemiology"[All Fields] OR "frequency"[All Fields] OR "epidemiology"[MeSH Terms] OR "frequence"[All Fields] OR "frequences"[All Fields] OR "frequencies"[All Fields]  **training:** "education"[Subheading] OR "education"[All Fields] OR "training"[All Fields] OR "education"[MeSH Terms] OR "train"[All Fields] OR "train's"[All Fields] OR "trained"[All Fields] OR "training's"[All Fields] OR "trainings"[All Fields] OR "trains"[All Fields]  **volume:** "volum"[All Fields] OR "volume"[All Fields] OR "volumes"[All Fields] OR "voluming"[All Fields]  **training:** "education"[Subheading] OR "education"[All Fields] OR "training"[All Fields] OR "education"[MeSH Terms] OR "train"[All Fields] OR "train's"[All Fields] OR "trained"[All Fields] OR "training's"[All Fields] OR "trainings"[All Fields] OR "trains"[All Fields]  **training:** "education"[Subheading] OR "education"[All Fields] OR "training"[All Fields] OR "education"[MeSH Terms] OR "train"[All Fields] OR "train's"[All Fields] OR "trained"[All Fields] OR "training's"[All Fields] OR "trainings"[All Fields] OR "trains"[All Fields]  **intensity:** "intense"[All Fields] OR "intensely"[All Fields] OR "intensities"[All Fields] OR "intensity"[All Fields] OR "intensively"[All Fields]  **muscle strength:** "muscle strength"[MeSH Terms] OR ("muscle"[All Fields] AND "strength"[All Fields]) OR "muscle strength"[All Fields]  **strength:** "strength"[All Fields] OR "strengths"[All Fields]  **muscle:** "muscle's"[All Fields] OR "muscles"[MeSH Terms] OR "muscles"[All Fields] OR "muscle"[All Fields]  **morphology:** "anatomy and histology"[Subheading] OR ("anatomy"[All Fields] AND "histology"[All Fields]) OR "anatomy and histology"[All Fields] OR "morphology"[All Fields] OR "morphologies"[All Fields]  **RM:** "Results Math"[Journal:__jid101763976] OR "rm"[All Fields]  **physical:** "physical examination"[MeSH Terms] OR ("physical"[All Fields] AND "examination"[All Fields]) OR "physical examination"[All Fields] OR "physical"[All Fields] OR "physically"[All Fields] OR "physicals"[All Fields]  **strength:** "strength"[All Fields] OR "strengths"[All Fields]  **force:** "force"[All Fields] OR "forceful"[All Fields] OR "forcefulness"[All Fields] OR "forces"[All Fields] OR "forcing"[All Fields] OR "forcings"[All Fields]  **development:** "develop"[All Fields] OR "develope"[All Fields] OR "developed"[All Fields] OR "developer"[All Fields] OR "developer's"[All Fields] OR "developers"[All Fields] OR "developing"[All Fields] OR "developments"[All Fields] OR "develops"[All Fields] OR "growth and development"[Subheading] OR ("growth"[All Fields] AND "development"[All Fields]) OR "growth and development"[All Fields] OR "development"[All Fields]  **strength:** "strength"[All Fields] OR "strengths"[All Fields]  **repetition:** "repetition"[All Fields] OR "repetitions"[All Fields]  **medline[Filter]:** "medlinestatus_medline"[all] | 6,991 |
