# Supplementary material for: The Influence of Individual Resistance Training Variables on Muscle Strength: A Systematic Review and Meta-analysis
Source: Sports Med. 2026 Apr 17;56(8):2001–15. doi: 10.1007/s40279-026-02432-5 (PMC13457492; doi:10.1007/s40279-026-02432-5)
Supplement: Supplementary file 5 [file 40279_2026_2432_MOESM3_ESM.docx]

**Supplementary Table 3.** Grading of relative chest, absolute chest, relative quadriceps, and absolute quadriceps outcomes.

| **No. of Studies** | **Risk of Bias** | **Inconsistency** | **Indirectness** | **Imprecision** | **Publication Bias** | **Large Effect** | **Plausible Confounding** | **Dose Response Gradient** | **Certainty of Evidence** |
| --- | --- | --- | --- | --- | --- | --- | --- | --- | --- |
| **Relative Chest** | | |  |  |  |  |  |  |  |
| 86 (3758 [Control = 1851, Exercise Intervention = 1907] participants) | Serious^a^ | Not Serious | Not Serious | Not Serious | Serious^b^ | No | No | Yes | High |
| **Absolute Chest** | | |  |  |  |  |  |  |  |
| 86 (3758 [Control = 1851, Exercise Intervention = 1907] participants) | Serious^a^ | Not Serious | Not Serious | Not Serious | Serious^b^ | No | No | Yes | High |
| **Relative Quadriceps** | | |  |  |  |  |  |  |  |
| 149 (6455 [Control = 3222, Exercise Intervention = 3233] participants) | Serious^a^ | Not Serious | Not Serious | Not Serious | Serious^b^ | No | No | Yes | High |
| **Absolute Quadriceps** | | |  |  |  |  |  |  |  |
| 149 (6455 [Control = 3222, Exercise Intervention = 3233] participants) | Serious^a^ | Not Serious | Not Serious | Not Serious | Serious^b^ | No | No | Yes | High |

**Note.** Explanations: ^a^ Based on the TESTEX Scale, there was a serious risk of bias from study methodological quality. A common limitation among the studies included in the meta-analysis was that they did not specify the method of randomisation despite being randomised controlled trails. In addition, allocation concealment, blinding of assessors, and intention to treat analyses were not regularly implemented among the selected studies. Consideration was applied to the grading due to the nature of clinical exercise studies, where blinding participants and assessors for this particular outcome to be very difficult. As such, the authors found the risk of bias to be serious and downgraded the quality of evidence by one. ^b^ This systematic review and meta-analysis included studies that compared exercise intervention groups against non-exercise control groups. Considering that the outcome of interest was muscle strength development, it is unlikely that non-exercise control groups would be favoured and therefore the publication of negative findings unlikely. Therefore, despite publication bias found to be serious, in the context of the outcome of interest and the intervention/control groups, the authors did not downgrade the quality of evidence.
