# Supplementary material for: The Influence of Individual Resistance Training Variables on Muscle Strength: A Systematic Review and Meta-analysis
Source: Sports Med. 2026 Apr 17;56(8):2001–15. doi: 10.1007/s40279-026-02432-5 (PMC13457492; doi:10.1007/s40279-026-02432-5)
Supplement: Supplementary file 6 [file 40279_2026_2432_MOESM4_ESM.docx]

**Supplementary Table 4**. Methodological quality appraisal for the chest musculature using the PEDro Scale. Quality evaluation results were categorised for each study into; low-risk (PEDro Scale ≥ 6 points), moderate-risk (PEDro Scale 4 to 5 points), and high-risk (PEDro Scale ≤ 3 points).

| **Author, Year** | **Criteria One (Not Counted)** | **Criteria Two** | **Criteria Three** | **Criteria Four** | **Criteria Five** | **Criteria Six** | **Criteria Seven** | **Criteria Eight** | **Criteria Nine** | **Criteria Ten** | **Criteria Eleven** | **Total** | **Quality Evaluation** |
| --- | --- | --- | --- | --- | --- | --- | --- | --- | --- | --- | --- | --- | --- |
| Abe et al., 2000 | YES | 1 | 1 | 1 | 0 | 0 | 0 | 0 | 0 | 1 | 1 | 5 | Moderate risk |
| Abdul-Al et al., 2024 | YES | 0 | 1 | 1 | 0 | 0 | 0 | 1 | 1 | 1 | 1 | 6 | Low risk |
| Ades et al., 1996 | YES | 1 | 0 | 0 | 0 | 0 | 0 | 1 | 0 | 1 | 1 | 4 | Moderate risk |
| Alcaraz et al., 2011 | YES | 1 | 1 | 0 | 0 | 0 | 0 | 0 | 0 | 1 | 1 | 4 | Moderate risk |
| Amiri et al., 2023 | YES | 0 | 1 | 1 | 0 | 0 | 0 | 1 | 1 | 1 | 1 | 6 | Low risk |
| Arazi et al., 2021 | YES | 1 | 1 | 1 | 0 | 0 | 0 | 1 | 1 | 1 | 1 | 7 | Low risk |
| Azarbayjani et al., 2014 | NO | 1 | 0 | 1 | 0 | 0 | 0 | 0 | 0 | 1 | 1 | 4 | Moderate risk |
| Bagheri et al. 2025 | YES | 1 | 1 | 1 | 0 | 0 | 0 | 1 | 1 | 1 | 0 | 6 | Low risk |
| Banks et al., 2024 | NO | 0 | 1 | 0 | 0 | 0 | 1 | 1 | 0 | 1 | 1 | 5 | Moderate risk |
| Beltran Valls et al., 2014 | YES | 1 | 1 | 1 | 0 | 0 | 0 | 0 | 0 | 1 | 1 | 5 | Moderate risk |
| Bermon et al., 1999 | YES | 1 | 0 | 1 | 0 | 0 | 0 | 1 | 0 | 1 | 1 | 5 | Moderate risk |
| Bezerra et al., 2018 | YES | 1 | 0 | 0 | 0 | 0 | 1 | 0 | 0 | 1 | 1 | 4 | Moderate risk |
| Bijeh et al., 2022 | YES | 1 | 1 | 1 | 0 | 1 | 1 | 1 | 1 | 1 | 1 | 9 | Low risk |
| Bocalini et al., 2009 | YES | 1 | 1 | 1 | 0 | 0 | 1 | 0 | 0 | 1 | 1 | 6 | Low risk |
| Brandon et al., 1997 | YES | 1 | 0 | 1 | 0 | 0 | 0 | 1 | 1 | 0 | 1 | 5 | Moderate risk |
| Calatayud et al., 2015 | YES | 1 | 1 | 1 | 0 | 0 | 0 | 0 | 0 | 1 | 1 | 5 | Moderate risk |
| Cassemiro et al., 2017 | YES | 1 | 0 | 0 | 0 | 0 | 0 | 0 | 0 | 1 | 1 | 3 | High risk |
| Cesar et al., 2009 | NO | 1 | 0 | 1 | 0 | 0 | 0 | 1 | 0 | 1 | 1 | 5 | Moderate risk |
| Coetsee et al., 2015 | YES | 1 | 0 | 1 | 0 | 0 | 0 | 1 | 0 | 1 | 1 | 5 | Moderate risk |
| Conceicao et al., 2013 | YES | 1 | 0 | 0 | 0 | 0 | 0 | 1 | 1 | 1 | 1 | 5 | Moderate risk |
| de Lima et al., 2012 | YES | 1 | 1 | 1 | 0 | 0 | 0 | 1 | 1 | 1 | 1 | 7 | Low risk |
| Donges et al., 2013 | YES | 1 | 1 | 0 | 0 | 0 | 0 | 1 | 1 | 1 | 1 | 6 | Low risk |
| Elliott et al., 2002 | YES | 1 | 0 | 0 | 0 | 0 | 0 | 1 | 0 | 0 | 1 | 3 | High risk |
| Farinatti et al., 2013 | YES | 1 | 0 | 1 | 0 | 0 | 1 | 1 | 0 | 1 | 1 | 6 | Low risk |
| Fatouros et al., 2006 | YES | 1 | 0 | 1 | 0 | 0 | 0 | 1 | 0 | 1 | 1 | 5 | Moderate risk |
| Fatouros et al., 2002 | YES | 1 | 0 | 1 | 0 | 0 | 0 | 1 | 0 | 1 | 1 | 5 | Moderate risk |
| Fatouros et al., 2005 | YES | 1 | 1 | 1 | 0 | 0 | 0 | 0 | 0 | 1 | 1 | 5 | Moderate risk |
| Filho et al., 2022 | YES | 0 | 1 | 1 | 0 | 0 | 0 | 0 | 0 | 1 | 1 | 4 | Moderate risk |
| Gerage et al., 2013 | YES | 1 | 0 | 0 | 0 | 0 | 1 | 1 | 0 | 1 | 1 | 5 | Moderate risk |
| Gualano et al., 2014 | YES | 1 | 1 | 1 | 0 | 1 | 1 | 0 | 0 | 1 | 1 | 7 | Low risk |
| Harber et al., 2004 | NO | 1 | 0 | 1 | 0 | 0 | 0 | 1 | 1 | 0 | 1 | 5 | Moderate risk |
| Haykowsky et al., 2000 | YES | 1 | 1 | 1 | 0 | 0 | 0 | 0 | 0 | 0 | 1 | 4 | Moderate risk |
| Hendrickson et al., 2010 | YES | 0 | 1 | 1 | 0 | 0 | 0 | 1 | 0 | 1 | 1 | 5 | Moderate risk |
| Henwood et al., 2006 | YES | 1 | 0 | 1 | 0 | 0 | 0 | 1 | 0 | 1 | 1 | 5 | Moderate risk |
| Henwood et al., 2008 | YES | 1 | 0 | 0 | 0 | 0 | 0 | 0 | 0 | 1 | 1 | 3 | High risk |
| Herda et al., 2020 | NO | 1 | 1 | 1 | 0 | 0 | 0 | 0 | 0 | 1 | 1 | 5 | Moderate risk |
| Hooshmand-Moghadam, et al. 2020 | YES | 1 | 1 | 1 | 0 | 0 | 0 | 1 | 1 | 1 | 1 | 7 | Low risk |
| Huschtscha et al., 2021 | YES | 1 | 1 | 1 | 0 | 0 | 0 | 1 | 0 | 1 | 1 | 6 | Low risk |
| Kalapotharakos et al., 2004 | YES | 1 | 1 | 1 | 0 | 0 | 0 | 1 | 1 | 1 | 1 | 7 | Low risk |
| Kell., 2011 | YES | 1 | 1 | 0 | 0 | 0 | 0 | 1 | 0 | 1 | 1 | 5 | Moderate risk |
| Kim et al., 2011 | NO | 1 | 1 | 1 | 0 | 0 | 0 | 1 | 1 | 1 | 1 | 7 | Low risk |
| Kirk et al., 2007 | YES | 1 | 1 | 1 | 0 | 0 | 0 | 0 | 0 | 1 | 1 | 5 | Moderate risk |
| Kraemer et al., 2004 | NO | 1 | 0 | 1 | 0 | 0 | 0 | 1 | 0 | 1 | 1 | 5 | Moderate risk |
| Lanza et al., 2024 | YES | 0 | 1 | 1 | 0 | 0 | 0 | 1 | 0 | 1 | 1 | 5 | Moderate risk |
| Leite et al., 2015 | YES | 1 | 0 | 0 | 0 | 0 | 1 | 0 | 0 | 1 | 1 | 4 | Moderate risk |
| Libardi et al., 2012 | YES | 1 | 0 | 1 | 0 | 0 | 0 | 1 | 1 | 1 | 1 | 6 | Low risk |
| Libardi et al., 2011 | YES | 1 | 0 | 1 | 0 | 0 | 0 | 0 | 0 | 1 | 1 | 4 | Moderate risk |
| Lo et al., 2011 | NO | 1 | 0 | 1 | 0 | 0 | 0 | 1 | 0 | 1 | 1 | 5 | Moderate risk |
| Manocchia et al., 2013 | YES | 1 | 0 | 1 | 0 | 0 | 0 | 0 | 0 | 0 | 1 | 3 | High risk |
| Marx et al., 2001 | NO | 1 | 1 | 1 | 0 | 0 | 1 | 1 | 1 | 1 | 1 | 8 | Low risk |
| Mate-Munoz et al., 2014 | NO | 1 | 0 | 1 | 0 | 0 | 0 | 0 | 0 | 1 | 1 | 4 | Moderate risk |
| McCartney et al., 1995 | YES | 1 | 0 | 0 | 0 | 0 | 0 | 1 | 0 | 1 | 1 | 4 | Moderate risk |
| McGinley et al. 2007 | NO | 1 | 1 | 0 | 0 | 0 | 1 | 0 | 0 | 1 | 1 | 5 | Moderate risk |
| Morgan et al., 2023 | YES | 0 | 1 | 1 | 0 | 0 | 0 | 1 | 1 | 1 | 1 | 6 | Low risk |
| Nichols et al., 1995 | YES | 1 | 0 | 0 | 0 | 0 | 0 | 1 | 0 | 1 | 1 | 4 | Moderate risk |
| Nichols et al., 1993 | YES | 1 | 1 | 1 | 0 | 0 | 0 | 0 | 0 | 1 | 1 | 5 | Moderate risk |
| Nunes et al., 2019 | YES | 1 | 0 | 1 | 0 | 0 | 0 | 1 | 0 | 1 | 1 | 5 | Moderate risk |
| Olson et al., 2006 | YES | 1 | 1 | 1 | 0 | 0 | 1 | 0 | 0 | 1 | 1 | 6 | Low risk |
| Panton et al., 2001 | NO | 1 | 0 | 1 | 0 | 0 | 0 | 1 | 0 | 1 | 1 | 5 | Moderate risk |
| Prestes et al., 2015 | YES | 1 | 0 | 1 | 0 | 0 | 0 | 0 | 1 | 1 | 1 | 5 | Moderate risk |
| Pyka et al., 1994 | YES | 1 | 0 | 1 | 0 | 0 | 0 | 1 | 0 | 1 | 1 | 5 | Moderate risk |
| Rabelo et al., 2004 | NO | 1 | 0 | 1 | 0 | 0 | 0 | 0 | 0 | 1 | 1 | 4 | Moderate risk |
| Ramirez-Campillo et al., 2014 | YES | 1 | 0 | 1 | 0 | 0 | 0 | 0 | 0 | 1 | 1 | 4 | Moderate risk |
| Rhodes et al., 2000 | YES | 1 | 0 | 1 | 0 | 0 | 0 | 1 | 0 | 1 | 1 | 5 | Moderate risk |
| Santos et al., 2018 | NO | 1 | 0 | 0 | 0 | 0 | 0 | 0 | 0 | 0 | 1 | 2 | High risk |
| Santos et al., 2010 | NO | 1 | 0 | 1 | 0 | 0 | 0 | 1 | 1 | 1 | 1 | 6 | Low risk |
| Shaw et al., 2009 | YES | 1 | 1 | 0 | 0 | 0 | 0 | 1 | 1 | 0 | 1 | 5 | Moderate risk |
| Schlumberger et al., 2001 | NO | 1 | 0 | 0 | 0 | 0 | 1 | 1 | 1 | 1 | 1 | 6 | Low risk |
| Simao et al., 2011 | YES | 1 | 1 | 1 | 0 | 0 | 1 | 1 | 1 | 1 | 1 | 8 | Low risk |
| Simao et al., 2012 | YES | 1 | 0 | 1 | 0 | 0 | 0 | 1 | 1 | 1 | 1 | 6 | Low risk |
| Simons et al., 2006 | YES | 1 | 0 | 1 | 0 | 0 | 1 | 1 | 0 | 0 | 1 | 5 | Moderate risk |
| Snow-Harter et al., 1992 | YES | 1 | 1 | 1 | 0 | 0 | 0 | 0 | 0 | 1 | 1 | 5 | Moderate risk |
| Spineti et al., 2010 | YES | 1 | 1 | 1 | 0 | 0 | 0 | 1 | 1 | 1 | 1 | 7 | Low risk |
| Taaffe et a., 1999 | YES | 1 | 0 | 1 | 0 | 0 | 0 | 1 | 0 | 1 | 1 | 5 | Moderate risk |
| Tanimoto et al., 2008 | NO | 1 | 1 | 1 | 0 | 0 | 0 | 1 | 1 | 1 | 1 | 7 | Low risk |
| Teixeira et al., 2019 | YES | 0 | 1 | 1 | 0 | 0 | 0 | 1 | 1 | 1 | 1 | 6 | Low risk |
| Terrazas et al., 2025 | NO | 1 | 1 | 1 | 0 | 0 | 0 | 1 | 1 | 1 | 1 | 7 | Low risk |
| Terzis et al., 2008 | NO | 1 | 1 | 0 | 0 | 0 | 0 | 0 | 0 | 1 | 1 | 4 | Moderate risk |
| Tomeleri et al., 2020 | YES | 0 | 1 | 1 | 0 | 1 | 1 | 0 | 0 | 1 | 1 | 6 | Low risk |
| Vale et al., 2017 | YES | 1 | 0 | 1 | 0 | 0 | 0 | 0 | 0 | 1 | 1 | 4 | Moderate risk |
| Vincent et al., 2002 | YES | 1 | 1 | 1 | 0 | 0 | 0 | 1 | 0 | 1 | 1 | 6 | Low risk |
| Wallerstein et al., 2012 | YES | 1 | 1 | 0 | 0 | 0 | 0 | 0 | 0 | 1 | 1 | 4 | Moderate risk |
| Willoughby et al., 1998 | YES | 1 | 1 | 1 | 0 | 0 | 0 | 0 | 0 | 1 | 1 | 5 | Moderate risk |
| Wilson et al., 1996 | NO | 1 | 1 | 1 | 0 | 0 | 0 | 1 | 0 | 0 | 1 | 5 | Moderate risk |
| Wood et al., 2001 | YES | 1 | 0 | 1 | 0 | 0 | 0 | 0 | 0 | 1 | 1 | 4 | Moderate risk |
| Yasuda et al., 2011 | NO | 1 | 0 | 1 | 0 | 0 | 0 | 0 | 0 | 1 | 1 | 4 | Moderate risk |

**Note.** Criteria One; eligibility criteria specified (no points awarded), criteria two; subjects were randomly allocated to groups (the precise method of randomisation is not necessary), criteria three; allocation was concealed, criteria four the groups were similar at baseline regarding the most important prognostic factors, criteria five; there was blinding of all subjects, criteria six; there was blinding of all therapists who administered the study, criteria seven; there was blinding of all assessors who measured at least one outcome, criteria eight; measures of at least one key outcome were obtained from more than 85% of the subjects initially allocated to groups, criteria nine; all subjects received the treatment or control, and if not, an intention to treat analysis was performed, criteria ten; the results of between-group statistical comparisons are reported for at least one key outcome, criteria eleven; the study provides both point measures and measures of variability for at least one key outcome.
