# Supplementary material for: The Influence of Individual Resistance Training Variables on Muscle Strength: A Systematic Review and Meta-analysis
Source: Sports Med. 2026 Apr 17;56(8):2001–15. doi: 10.1007/s40279-026-02432-5 (PMC13457492; doi:10.1007/s40279-026-02432-5)
Supplement: Supplementary file 7 [file 40279_2026_2432_MOESM5_ESM.docx]

**Supplementary Table 5**. Methodological quality appraisal for the chest musculature using the TESTEX Checklist. Quality evaluation results were categorised for each study into; low-risk (TESTex Scale ≥ 11 points), moderate-risk (TESTEX Scale 6 to 10 points), and high-risk (TESTEX Scale ≤ 5 points).

|  | Study Quality (Possible Points = 5) | | | | | Study Reporting (Possible Points = 10) | | | | | | |  |  |
| --- | --- | --- | --- | --- | --- | --- | --- | --- | --- | --- | --- | --- | --- | --- |
| Author, Year | Criteria One | Criteria Two | Criteria Three | Criteria Four | Criteria Five | Criteria Six | Criteria Seven | Criteria Eight | Criteria Nine | Criteria Ten | Criteria Eleven | Criteria Twelve | Total | Quality Evaluation |
| Abe et al., 2000 | 1 | 0 | 1 | 1 | 0 | 0 | 0 | 2 | 1 | 0 | 1 | 1 | 8 | Moderate risk |
| Abdul-Al et al., 2024 | 1 | 0 | 1 | 1 | 0 | 1 | 1 | 2 | 1 | 0 | 0 | 1 | 9 | Moderate risk |
| Ades et al. 1996 | 1 | 0 | 0 | 0 | 0 | 1 | 0 | 2 | 1 | 0 | 1 | 1 | 7 | Moderate risk |
| Alcaraz et al., 2011 | 1 | 0 | 1 | 0 | 0 | 1 | 0 | 2 | 1 | 0 | 1 | 1 | 8 | Moderate risk |
| Amiri et al., 2023 | 1 | 0 | 1 | 1 | 0 | 1 | 1 | 2 | 1 | 0 | 1 | 1 | 10 | Moderate risk |
| Arazi et al., 2021 | 1 | 1 | 1 | 1 | 0 | 1 | 1 | 2 | 1 | 0 | 1 | 1 | 11 | Low risk |
| Azarbayjani et al., 2014 | 0 | 0 | 0 | 1 | 0 | 0 | 0 | 2 | 1 | 0 | 1 | 1 | 6 | Moderate risk |
| Bagheri et al. 2025 | 1 | 1 | 1 | 1 | 0 | 3 | 1 | 2 | 0 | 0 | 1 | 1 | 12 | Low risk |
| Banks et al., 2024 | 0 | 0 | 1 | 0 | 1 | 2 | 0 | 2 | 1 | 1 | 1 | 1 | 10 | Moderate risk |
| Beltran Valls et al., 2014 | 1 | 0 | 1 | 1 | 0 | 1 | 0 | 2 | 1 | 0 | 1 | 1 | 9 | Moderate risk |
| Bermon et al., 1999 | 1 | 0 | 0 | 1 | 0 | 2 | 0 | 2 | 1 | 0 | 1 | 1 | 9 | Moderate risk |
| Bezerra et al., 2018 | 1 | 0 | 0 | 0 | 1 | 2 | 0 | 2 | 1 | 0 | 1 | 1 | 9 | Moderate risk |
| Bijeh et al., 2022 | 1 | 1 | 1 | 1 | 1 | 1 | 1 | 2 | 1 | 0 | 1 | 1 | 12 | Low risk |
| Bocalini et al., 2009 | 1 | 0 | 1 | 1 | 1 | 2 | 0 | 2 | 1 | 0 | 1 | 1 | 11 | Low risk |
| Brandon et al., 1997 | 1 | 0 | 0 | 1 | 0 | 1 | 1 | 0 | 1 | 0 | 1 | 1 | 7 | Moderate risk |
| Calatayud et al., 2015 | 1 | 0 | 1 | 1 | 0 | 0 | 0 | 2 | 1 | 0 | 0 | 1 | 7 | Moderate risk |
| Cassemiro et al., 2017 | 1 | 0 | 0 | 0 | 0 | 0 | 0 | 2 | 1 | 0 | 0 | 1 | 5 | High risk |
| Cesar et al., 2009 | 0 | 0 | 0 | 1 | 0 | 1 | 0 | 1 | 1 | 0 | 1 | 1 | 6 | Moderate risk |
| Coetsee et al., 2015 | 1 | 1 | 0 | 1 | 0 | 2 | 0 | 2 | 1 | 0 | 1 | 1 | 10 | Moderate risk |
| Conceicao et al., 2013 | 1 | 0 | 0 | 0 | 0 | 1 | 1 | 2 | 1 | 0 | 1 | 1 | 8 | Moderate risk |
| de Lima et al., 2012 | 1 | 0 | 1 | 1 | 0 | 1 | 1 | 2 | 1 | 0 | 1 | 1 | 10 | Moderate risk |
| Donges et al., 2013 | 1 | 0 | 1 | 0 | 0 | 2 | 1 | 2 | 1 | 0 | 1 | 1 | 10 | Moderate risk |
| Elliott et al., 2002 | 1 | 0 | 0 | 0 | 0 | 2 | 0 | 0 | 1 | 1 | 1 | 1 | 7 | Moderate risk |
| Farinatti et al., 2013 | 1 | 1 | 0 | 1 | 1 | 3 | 0 | 2 | 1 | 0 | 1 | 1 | 12 | Low risk |
| Fatouros et al., 2006 | 1 | 0 | 0 | 1 | 0 | 3 | 0 | 2 | 1 | 0 | 1 | 1 | 10 | Moderate risk |
| Fatouros et al., 2002 | 1 | 0 | 0 | 1 | 0 | 3 | 0 | 2 | 1 | 0 | 1 | 1 | 10 | Moderate risk |
| Fatouros et al., 2005 | 1 | 0 | 1 | 1 | 0 | 2 | 0 | 2 | 1 | 0 | 1 | 1 | 10 | Moderate risk |
| Filho et al., 2022 | 1 | 0 | 1 | 1 | 0 | 0 | 0 | 1 | 1 | 0 | 1 | 1 | 7 | Moderate risk |
| Gerage et al., 2013 | 1 | 0 | 0 | 0 | 1 | 3 | 0 | 2 | 1 | 0 | 1 | 1 | 10 | Moderate risk |
| Gualano et al., 2014 | 1 | 1 | 1 | 1 | 1 | 0 | 0 | 2 | 1 | 0 | 1 | 1 | 10 | Moderate risk |
| Harber et al., 2004 | 0 | 0 | 0 | 1 | 0 | 1 | 1 | 0 | 1 | 0 | 1 | 1 | 6 | Moderate risk |
| Haykowsky et al., 2000 | 1 | 0 | 1 | 1 | 0 | 2 | 0 | 0 | 1 | 0 | 1 | 0 | 7 | Moderate risk |
| Hendrickson et al., 2010 | 1 | 0 | 1 | 1 | 0 | 1 | 0 | 2 | 1 | 0 | 1 | 1 | 9 | Moderate risk |
| Henwood et al., 2006 | 1 | 0 | 0 | 1 | 0 | 3 | 0 | 2 | 1 | 0 | 1 | 1 | 10 | Moderate risk |
| Henwood et al., 2008 | 1 | 0 | 0 | 0 | 0 | 2 | 0 | 2 | 1 | 0 | 1 | 1 | 8 | Moderate risk |
| Herda et al., 2020 | 0 | 1 | 1 | 1 | 0 | 0 | 0 | 2 | 1 | 0 | 1 | 0 | 7 | Moderate risk |
| Hooshmand-Moghadam, et al. 2020 | 1 | 1 | 1 | 1 | 0 | 2 | 1 | 1 | 1 | 0 | 1 | 1 | 11 | Low risk |
| Huschtscha et al., 2021 | 1 | 1 | 1 | 1 | 0 | 3 | 0 | 2 | 1 | 1 | 1 | 1 | 13 | Low risk |
| Kalapotharakos et al., 2004 | 1 | 0 | 1 | 1 | 0 | 2 | 1 | 2 | 1 | 0 | 1 | 1 | 11 | Low risk |
| Kell., 2011 | 1 | 0 | 1 | 0 | 0 | 1 | 0 | 2 | 1 | 0 | 1 | 1 | 8 | Moderate risk |
| Kim et al., 2011 | 0 | 0 | 1 | 1 | 0 | 1 | 1 | 2 | 1 | 0 | 1 | 1 | 9 | Moderate risk |
| Kirk et al., 2007 | 1 | 0 | 1 | 1 | 0 | 2 | 0 | 2 | 1 | 0 | 1 | 1 | 10 | Moderate risk |
| Kraemer et al., 2004 | 0 | 0 | 0 | 1 | 0 | 2 | 0 | 2 | 1 | 0 | 1 | 1 | 8 | Moderate risk |
| Lanza et al., 2024 | 1 | 0 | 1 | 1 | 0 | 1 | 0 | 1 | 1 | 0 | 0 | 1 | 7 | Moderate risk |
| Leite et al., 2015 | 1 | 0 | 0 | 0 | 1 | 0 | 0 | 1 | 1 | 0 | 1 | 1 | 6 | Moderate risk |
| Libardi et al., 2012 | 1 | 0 | 0 | 1 | 0 | 2 | 1 | 2 | 1 | 0 | 1 | 1 | 10 | Moderate risk |
| Libardi et al., 2011 | 1 | 0 | 0 | 1 | 0 | 0 | 0 | 2 | 1 | 0 | 1 | 1 | 7 | Moderate risk |
| Lo et al., 2011 | 0 | 0 | 0 | 1 | 0 | 2 | 0 | 2 | 1 | 0 | 1 | 1 | 8 | Moderate risk |
| Manocchia et al., 2013 | 1 | 0 | 0 | 1 | 0 | 2 | 0 | 0 | 1 | 0 | 1 | 0 | 6 | Moderate risk |
| Marx et al., 2001 | 0 | 0 | 1 | 1 | 1 | 3 | 1 | 2 | 1 | 0 | 1 | 1 | 12 | Low risk |
| Mate-Munoz et al., 2014 | 0 | 0 | 0 | 1 | 0 | 0 | 0 | 2 | 1 | 0 | 1 | 0 | 5 | High risk |
| McCartney et al., 1995 | 1 | 0 | 0 | 0 | 0 | 3 | 0 | 2 | 1 | 0 | 1 | 1 | 9 | Moderate risk |
| McGinley et al. 2007 | 0 | 0 | 1 | 0 | 1 | 0 | 0 | 2 | 1 | 0 | 1 | 1 | 7 | Moderate risk |
| Morgan et al., 2023 | 1 | 0 | 1 | 1 | 0 | 1 | 1 | 2 | 1 | 0 | 1 | 1 | 10 | Moderate risk |
| Nichols et al., 1995 | 1 | 0 | 0 | 0 | 0 | 3 | 0 | 2 | 1 | 0 | 0 | 1 | 8 | Moderate risk |
| Nichols et al., 1993 | 1 | 0 | 1 | 1 | 0 | 2 | 0 | 1 | 1 | 0 | 1 | 1 | 9 | Moderate risk |
| Nunes et al., 2019 | 1 | 0 | 0 | 1 | 0 | 2 | 0 | 2 | 1 | 0 | 1 | 1 | 9 | Moderate risk |
| Olson et al., 2006 | 1 | 0 | 1 | 1 | 1 | 1 | 0 | 2 | 1 | 0 | 1 | 1 | 10 | Moderate risk |
| Panton et al., 2001 | 0 | 0 | 0 | 1 | 0 | 1 | 0 | 2 | 1 | 0 | 1 | 1 | 7 | Moderate risk |
| Prestes et al., 2015 | 1 | 0 | 0 | 1 | 0 | 0 | 1 | 2 | 1 | 0 | 1 | 1 | 8 | Moderate risk |
| Pyka et al., 1994 | 1 | 0 | 0 | 1 | 0 | 3 | 0 | 1 | 1 | 0 | 1 | 1 | 9 | Moderate risk |
| Rabelo et al., 2004 | 0 | 0 | 0 | 1 | 0 | 0 | 0 | 2 | 1 | 0 | 1 | 1 | 6 | Moderate risk |
| Ramirez-Campillo et al., 2014 | 1 | 0 | 0 | 1 | 0 | 0 | 0 | 2 | 1 | 0 | 1 | 1 | 7 | Moderate risk |
| Rhodes et al., 2000 | 1 | 0 | 0 | 1 | 0 | 3 | 0 | 1 | 1 | 0 | 1 | 1 | 9 | Moderate risk |
| Santos et al., 2018 | 0 | 0 | 0 | 0 | 0 | 0 | 0 | 0 | 1 | 0 | 1 | 1 | 3 | High risk |
| Santos et al., 2010 | 0 | 0 | 0 | 1 | 0 | 1 | 1 | 2 | 1 | 0 | 1 | 1 | 8 | Moderate risk |
| Shaw et al., 2009 | 1 | 0 | 1 | 0 | 0 | 1 | 1 | 0 | 1 | 0 | 1 | 1 | 7 | Moderate risk |
| Schlumberger et al., 2001 | 0 | 0 | 0 | 0 | 1 | 2 | 1 | 1 | 1 | 0 | 1 | 1 | 8 | Moderate risk |
| Simao et al., 2011 | 1 | 0 | 1 | 1 | 1 | 1 | 1 | 2 | 1 | 0 | 1 | 1 | 11 | Low risk |
| Simao et al., 2012 | 1 | 0 | 0 | 1 | 0 | 2 | 1 | 1 | 1 | 0 | 1 | 1 | 9 | Moderate risk |
| Simons et al., 2006 | 1 | 0 | 0 | 1 | 1 | 2 | 0 | 0 | 1 | 0 | 1 | 1 | 8 | Moderate risk |
| Snow-Harter et al., 1992 | 1 | 0 | 1 | 1 | 0 | 2 | 0 | 2 | 1 | 1 | 1 | 1 | 11 | Low risk |
| Spineti et al., 2010 | 1 | 0 | 1 | 1 | 0 | 2 | 1 | 2 | 1 | 0 | 1 | 1 | 11 | Low risk |
| Taaffe et a., 1999 | 1 | 0 | 0 | 1 | 0 | 2 | 0 | 2 | 1 | 0 | 1 | 1 | 9 | Moderate risk |
| Tanimoto et al., 2008 | 0 | 0 | 1 | 1 | 0 | 1 | 1 | 2 | 1 | 0 | 1 | 1 | 9 | Moderate risk |
| Teixeira et al., 2019 | 1 | 0 | 1 | 1 | 0 | 1 | 1 | 1 | 1 | 0 | 1 | 1 | 9 | Moderate risk |
| Terrazas et al., 2025 | 0 | 1 | 1 | 1 | 0 | 1 | 1 | 2 | 1 | 0 | 1 | 1 | 10 | Moderate risk |
| Terzis et al., 2008 | 0 | 0 | 1 | 0 | 0 | 2 | 0 | 2 | 1 | 0 | 1 | 1 | 8 | Moderate risk |
| Tomeleri et al., 2020 | 1 | 0 | 1 | 1 | 1 | 0 | 0 | 2 | 1 | 0 | 1 | 1 | 9 | Moderate risk |
| Vale et al., 2017 | 1 | 0 | 0 | 1 | 0 | 2 | 0 | 2 | 1 | 0 | 1 | 1 | 9 | Moderate risk |
| Vincent et al., 2002 | 1 | 1 | 1 | 1 | 0 | 3 | 0 | 2 | 1 | 0 | 1 | 1 | 12 | Low risk |
| Wallerstein et al., 2012 | 1 | 0 | 1 | 0 | 0 | 0 | 0 | 2 | 1 | 0 | 1 | 1 | 7 | Moderate risk |
| Willoughby et al., 1998 | 1 | 0 | 1 | 1 | 0 | 0 | 0 | 1 | 1 | 0 | 1 | 1 | 7 | Moderate risk |
| Wilson et al., 1996 | 0 | 1 | 1 | 1 | 0 | 2 | 0 | 0 | 1 | 0 | 1 | 1 | 8 | Moderate risk |
| Wood et al., 2001 | 1 | 0 | 1 | 1 | 0 | 2 | 0 | 2 | 1 | 0 | 1 | 1 | 10 | Moderate risk |
| Yasuda et al., 2011 | 0 | 0 | 0 | 1 | 0 | 0 | 0 | 2 | 1 | 0 | 1 | 1 | 6 | Moderate risk |

**Note.** Criteria one; eligibility criteria specified, criteria two; randomisation specified, criteria three; allocation concealment of all patients at the time of randomisation, criteria four; groups similar at baseline, criteria five; blinding of assessor for at least one key outcome, criteria six; outcome measures assessed in at least 85% of patients, criteria seven; intention to treat analysis conducted when a patient withdraws, criteria eight; between group statistical comparisons reported, criteria nine; point measures and measures of variability for all reported outcome measures, criteria ten; activity monitoring in control groups, criteria eleven; relative exercise intensity remained constant, criteria twelve; exercise volume and energy expenditure clearly reported.
