# Supplementary material for: The Influence of Individual Resistance Training Variables on Muscle Strength: A Systematic Review and Meta-analysis
Source: Sports Med. 2026 Apr 17;56(8):2001–15. doi: 10.1007/s40279-026-02432-5 (PMC13457492; doi:10.1007/s40279-026-02432-5)
Supplement: Supplementary file 10 [file 40279_2026_2432_MOESM8_ESM.docx]

**Supplementary Table 8.** Population Demographic and Intervention Data

| **Article** | **Group Assignment** | **Training Status** | **Sample Size  M (F / MF)** | **Age: Mean (Years)** | **Mass (kg)** | **Stature (cm)** | **Strength Assessment** | **Functional Movement** | **Number of Sets (Median)** | **Number of Repetitions (Median)** | **Number of Exercises** | **Intensity (%1RM)** | **Total Prescribed Absolute Intensity (kg)** | **Frequency (Median)** | **Duration (Weeks)** |
| --- | --- | --- | --- | --- | --- | --- | --- | --- | --- | --- | --- | --- | --- | --- | --- |
| Abdul-Al et al., 2024 | Control 1 | Untrained | 0 (17/0) | 65.47±2.52 | 68.44±16.31 | 152±5 | 1RM | Bench Press / Leg Press |  |  |  |  |  |  |  |
|  | Exercise 1 |  | 0 (16/0) | 65.18±3.52 | 66.56±11.45 | 156±7 |  |  | 3 | 10 | 8 | 75 | 19042 | 2 | 52 |
|  | Exercise 2 |  | 0 (16/0) | 65.41±3.43 | 69.53±11.04 | 155±5 |  |  | 3 | 10 | 8 | 75 | 18897 | 2 | 52 |
| Abe et al., 2000 | Control 1 | Untrained | 6 (0/0) | 42.5±7.2 | 87.3±13.1 | 177.6±8.5 | 1RM | Knee Extension / Chest Press |  |  |  |  |  |  |  |
|  | Control 2 |  | 0 (7/0) | 44.6±5.7 | 63.6±9.6 | 165.4±7.6 |  |  |  |  |  |  |  |  |  |
|  | Exercise 1 |  | 17 (0/0) | 37.7±7.2 | 87.6±12.8 | 178.4±4.9 |  |  | 2 | 10 | 6 | 65 | 9374 | 3 | 12 |
|  | Exercise 2 |  | 0 (20/0) | 41±4.1 | 64.7±9.3 | 164.3±6.9 |  |  | 2 | 10 | 6 | 65 | 4511 | 3 | 12 |
| Ades et al., 1996 | Control 1 | Untrained | 5 (0/0) | 70.7±5 | 76.5±10 |  | 1RM | Knee Extension / Bench Press |  |  |  |  |  |  |  |
|  | Control 2 |  | 0 (7/0) |  | 71.9±12.5 |  |  |  |  |  |  |  |  |  |  |
|  | Exercise 1 |  | 6 (0/0) | 69.9±4 | 79.5±12 |  |  |  | 3 | 8 | 7 | 65 | 2153 | 3 | 12 |
|  | Exercise 2 |  | 0 (6/0) |  | 70.9±9.6 |  |  |  | 3 | 8 | 7 | 65 | 1193 | 3 | 12 |
| Aguiar et al., 2015 | Control 1 | Untrained | 9 (0/0) | 20±1.8 | 75±8.8 | 176.4±8.1 | 1RM | Knee Extension |  |  |  |  |  |  |  |
|  | Exercise 1 |  | 9 (0/0) | 21.1±1.8 | 73.3±6.8 | 177.4±7 |  |  | 3 | 18 | 1 | 50 | 841 | 2 | 8 |
|  | Exercise 2 |  | 9 (0/0) | 21.5±2.6 | 76.3±5.1 | 178.6±4.2 |  |  | 4 | 18 | 1 | 50 | 861 | 2 | 8 |
| Ahtiainen et al., 2011 | Control 1 | Untrained | 10 (0/0) | 25±3 | 76±11 | 185±4 | 1RM | Leg Press |  |  |  |  |  |  |  |
|  | Control 2 |  | 18 (0/0) | 64±8 | 76±4 | 172±8 |  |  |  |  |  |  |  |  |  |
|  | Exercise 1 |  | 7 (0/0) | 28±5 | 79±6 | 185±3 |  |  | 5 | 9 | 8 | 65 | 5321 | 2 | 21 |
|  | Exercise 2 |  | 8 (0/0) | 61±5 | 80±5 | 177±3 |  |  | 5 | 9 | 8 | 65 | 4778 | 2 | 21 |
| Alcaraz et al., 2011 | Control 1 | Trained | 7 (0/0) | 22.7±3.3 | 75.2±8.1 | 176±60 | 1RM | Bench Press |  |  |  |  |  |  |  |
|  | Exercise 1 |  | 15 (0/0) |  |  |  |  |  | 5 | 6 | 6 | 88 | 6172 | 3 | 8 |
|  | Exercise 2 |  | 11 (0/0) |  |  |  |  |  | 5 | 6 | 6 | 88 | 5731 | 3 | 8 |
| Amiri & Sheikholeslami-Vatani 2023 | Control 1 | Untrained | 0 (0/15) | 62.5±5.7 | 74.08±18.8 | 168.16±9.5 | 1RM | Bench Press / Knee Extension |  |  |  |  |  |  |  |
|  | Exercise 1 |  | 0 (0/15) | 61.3±5.7 | 75.37±10.7 | 162.37±6.35 |  |  | 3 | 10 | 7 | 75 | 2773 | 3 | 10 |
| Apel et al., 2011 | Control 1 | Trained | 14 (0/0) | 22±2.3 | 75.7±7.1 | 177±5 | 10RM | Barbell Squat |  |  |  |  |  |  |  |
|  | Exercise 1 |  | 14 (0/0) | 23±2.8 | 77±7.9 | 178±6 |  |  | 4 | 7 | 9 | 73 | 9419 | 4 | 12 |
|  | Exercise 2 |  | 14 (0/0) | 22±1.9 | 81.8±9.7 | 177±6 |  |  | 4 | 6 | 9 | 73 | 9339 | 4 | 12 |
| Arazi et al., 2021 | Control 1 | Trained | 10 (0/0) | 20.4±1.4 | 69.1±8 | 178±8 | 1RM | Bench Press / Leg Press |  |  |  |  |  |  |  |
|  | Exercise 1 |  | 12 (0/0) | 19.8±1.8 | 64.2±5.7 | 175±5 |  |  | 4 | 9 | 7 | 75 | 3948 | 2 | 8 |
|  | Exercise 2 |  | 13 (0/0) | 19.9±1.6 | 70.6±8.2 | 177±4 |  |  | 2 | 9 | 7 | 75 | 7994 | 4 | 8 |
| Arsenis et al., 2021 | Control 1 | Untrained | 0 (0/16) | 20.75±1.12 | 76.81±7.38 | 179.81±4.66 | Isokinetic Dynamometer | Knee Extension |  |  |  |  |  |  |  |
|  | Exercise 1 |  | 0 (0/16) | 20.63±1.45 | 75.94±7.8 | 176±4.14 |  |  | 4 | 6 | 1 |  |  | 2 | 8 |
| Bagheri et al., 2025 | Control 1 | Untrained | 15 (0/0) | 31±4 | 83.3±6.6 | 170±4 | 10RM | Bench Press / Leg Press |  |  |  |  |  |  |  |
|  | Exercise 1 |  | 15 (0/0) | 30±4 | 82.8±6 | 170.9±6.8 |  |  | 4 | 11 | 6 | 65 | 852 | 3 | 12 |
|  | Exercise 2 |  | 15 (0/0) | 30±3 | 83.4±5.9 | 171.2±4 |  |  | 4 | 11 | 6 | 65 | 1280 | 3 | 12 |
|  | Exercise 3 |  | 15 (0/0) | 31±5 | 82.6±6 | 171.3±3.7 |  |  | 4 | 11 | 6 | 65 | 803 | 3 | 12 |
| Banks et al., 2024 | Control 1 | Trained | 3 (5/0) | 21.38±2.2 | 68±8.9 | 169±10 | 1RM | Bench Press / Squat |  |  |  |  |  |  |  |
|  | Exercise 1 |  | 5 (7/0) | 25.75±3 | 74.5±14.3 | 168±10 |  |  | 3 | 8 | 5 |  |  | 3 | 10 |
| Bell et al., 2000 | Control 1 | Trained | 5 (0/0) | 22.3±3.3 | 73.4±11.6 | 176±9.3 | 1RM | Leg Press |  |  |  |  |  |  |  |
|  | Control 2 |  | 0 (5/0) |  |  |  |  |  |  |  |  |  |  |  |  |
|  | Exercise 1 |  | 7 (0/0) |  |  |  |  |  | 4 | 8 | 8 | 78 | 9346 | 3 | 12 |
|  | Exercise 2 |  | 0 (4/0) |  |  |  |  |  | 4 | 8 | 8 | 78 | 5317 | 3 | 12 |
| Beltran Valls et al., 2014 | Control 1 | Untrained | 0 (0/10) | 72±3.2 | 72±9.49 |  | 1RM | Knee Extension / Chest Press |  |  |  |  |  |  |  |
|  | Exercise 2 |  | 0 (0/13) | 72±3.6 | 70±7.2 |  |  |  | 4 | 11 | 4 | 70 | 2876 | 2 | 12 |
| Bemben et al., 2022 | Control 1 | Untrained | 8 (0/0) | 25.6±4 | 84.8±17.2 | 172.9±3.6 | 1RM | Knee Extension |  |  |  |  |  |  |  |
|  | Exercise 1 |  | 12 (0/0) | 20.9±2.9 | 82.8±24.4 | 179.9±7.5 |  |  | 3 | 10 | 6 | 65 | 4719 | 3 | 6 |
|  | Exercise 2 |  | 9 (0/0) | 20.6±1.7 | 71±7.8 | 176.7±5.7 |  |  | 3 | 13 | 6 | 48 | 3545 | 3 | 6 |
| Beneka et al., 2005 | Control 1 | Untrained | 8 (0/0) | 68.9±4 | 89.1±11.4 | 167±50 | Isokinetic Dynamometer | Knee Extension |  |  |  |  |  |  |  |
|  | Control 2 |  | 0 (8/0) | 65.7±4.3 | 73.4±8.6 | 162±90 |  |  |  |  |  |  |  |  |  |
|  | Exercise 1 |  | 8 (0/0) | 70.7±2.3 | 87.8±12.5 | 165±90 |  |  | 3 | 13 | 3 | 50 |  | 3 | 16 |
|  | Exercise 2 |  | 0 (8/0) | 68±5.1 | 75.1±9.3 | 159±80 |  |  | 3 | 9 | 3 | 70 |  | 3 | 16 |
|  | Exercise 3 |  | 8 (0/0) | 71.7±2.7 | 90.4±10.2 | 163±80 |  |  | 3 | 5 | 3 | 90 |  | 3 | 16 |
|  | Exercise 4 |  | 0 (8/0) | 66±5.5 | 73.9±8.8 | 161±50 |  |  | 3 | 13 | 3 | 50 |  | 3 | 16 |
|  | Exercise 5 |  | 8 (0/0) | 70.8±3.2 | 87.2±14.2 | 166±30 |  |  | 3 | 9 | 3 | 70 |  | 3 | 16 |
|  | Exercise 6 |  | 0 (8/0) | 68.9±6.7 | 77.2±6.9 | 162±30 |  |  | 3 | 5 | 3 | 90 |  | 3 | 16 |
| Bermon et al., 1999 | Control 1 | Untrained | 0 (0/16) | 70.5±3.6 |  |  | 1RM | Leg Press / Bench Press |  |  |  |  |  |  |  |
|  | Exercise 1 |  | 0 (0/16) | 70.1±4 |  |  |  |  | 3 | 8 | 3 | 80 |  | 3 | 8 |
| Bezerra et al., 2018 | Control 1 | Untrained | 0 (0/8) | 63±6.96 | 64.51±5.38 | 163±70 | 1) 5RM; 2) 12RM | 1) Leg Press; 2) Chest Press |  |  |  |  |  |  |  |
|  | Exercise 1 |  | 0 (0/11) | 64.27±5.33 | 80.98±15.72 | 170±8 |  |  | 3 | 7 | 6 |  |  | 3 | 9 |
|  | Exercise 2 |  | 0 (0/11) | 65±4.21 | 74.62±15.95 | 166±9 |  |  | 3 | 7 | 6 |  |  | 3 | 9 |
| Bijeh et al., 2022 | Control 1 | Untrained | 15 (0/0) | 65.63±3.16 | 61.85±3.79 |  | 1RM | Chest Press / Leg Press |  |  |  |  |  |  |  |
|  | Exercise 1 |  | 15 (0/0) |  | 65.85±3.74 |  |  |  | 4 | 15 | 6 | 60 | 2074 | 3 | 12 |
| Bishop et al., 1996 | Control 1 | Untrained | 8 (0/0) | 20.50 |  |  | 1RM | Leg Press |  |  |  |  |  |  |  |
|  | Exercise 1 |  | 8 (0/0) |  |  |  |  |  | 5 | 7 | 4 |  |  | 4 | 6 |
| Bocalini et al., 2009 | Control 1 | Untrained | 0 (12/0) | 67±8 | 69±7 |  | 1RM | Knee Extension / Chest Press |  |  |  |  |  |  |  |
|  | Exercise 1 |  | 0 (23/0) | 69±9 | 68±6 |  |  |  | 3 | 10 | 12 | 85 | 3550 | 3 | 24 |
| Botton et al., 2016 | Control 1 | Untrained | 0 (14/0) | 22.7±2.8 | 58±5.7 | 163.6±6.2 | 1RM | Knee Extension |  |  |  |  |  |  |  |
|  | Exercise 1 |  | 0 (15/0) | 24.3±3.7 | 57±4.8 | 160.2±5.8 |  |  | 3 | 10 | 8 |  |  | 2 | 12 |
| Brandon et al., 1997 | Control 1 | Untrained | 0 (0/42) | 72.1±5.3 | 72.7±11.9 | 164.9±8.4 | 1RM | Knee Extension / Arm Cross |  |  |  |  |  |  |  |
|  | Exercise 1 |  | 0 (0/43) | 72.1±5.5 | 72.4±12.3 | 165.6±9.7 |  |  | 3 | 10 | 11 | 60 | 11647 | 3 | 16 |
| Brandon et al., 2004 | Control 1 | Untrained | 0 (0/25) | 71.4±7 | 73.4±12.8 | 165.2±8 | 1RM | Knee Extension |  |  |  |  |  |  |  |
|  | Exercise 1 |  | 0 (0/30) | 71.8±6 | 73.5±14.2 | 165±7 |  |  | 3 | 10 | 11 | 60 |  | 2 | 104 |
| Calatayud et al., 2015 | Control 1 | Trained | 0 (0/10) | 21.9±2.1 | 67.5±6.3 | 171.6±7.6 | 1RM | Bench Press |  |  |  |  |  |  |  |
|  | Exercise 1 |  | 0 (0/10) | 22.7±3.3 | 67.7±8.8 | 173.1±7 |  |  | 5 | 6 | 1 |  |  | 2 | 5 |
| Campos et al., 2002 | Control 1 | Untrained | 5 (0/0) | 31.6±9.8 | 80.8±23.3 | 178.1±5.5 | 1RM | Barbell Squat |  |  |  |  |  |  |  |
|  | Exercise 1 |  | 9 (0/0) | 21.1±1.5 | 80.1±8.4 | 179.8±5.8 |  |  | 4 | 4 | 3 |  |  | 3 | 8 |
|  | Exercise 2 |  | 11 (0/0) | 20.7±2.9 | 79.5±7.8 | 179.6±7.4 |  |  | 3 | 10 | 3 |  |  | 3 | 8 |
|  | Exercise 3 |  | 7 (0/0) | 20.4±3.5 | 70.2±9.5 | 174.3±8.6 |  |  | 2 | 24 | 3 |  |  | 3 | 8 |
| Carroll et al., 1998 | Control 1 | Untrained | 0 (0/6) | 18.8±1.7 | 67.4±3 | 176±4 | 1RM | Barbell Squat |  |  |  |  |  |  |  |
|  | Exercise 1 |  | 0 (0/5) | 19.4±0.9 | 71.1±18.7 | 178±11 |  |  | 3 | 10 | 7 |  |  | 2 | 9 |
|  | Exercise 2 |  | 0 (0/6) | 17.7±0.5 | 69.5±11.6 | 175±11 |  |  | 3 | 10 | 7 |  |  | 3 | 6 |
| Cassemiro et al., 2017 | Control 1 | Untrained | 0 (13/0) | 20.6±1.1 | 56.35±7.51 | 162±27 | 1RM | Leg Press / Pectoral |  |  |  |  |  |  |  |
|  | Exercise 1 |  | 0 (14/0) | 22.5±1.78 | 53.81±6.5 | 161±7 |  |  | 3 | 6 | 11 | 80 | 9915 | 3 | 12 |
| Cesar et al., 2009 | Control 1 | Untrained | 0 (10/0) | 20.3±1.3 | 58.1±8.8 | 166±6 | 1RM | Leg Press / Bench Press |  |  |  |  |  |  |  |
|  | Exercise 1 |  | 0 (9/0) | 21±2.9 | 57.2±7.9 | 166±9 |  |  | 3 | 15 | 8 |  |  | 3 | 12 |
| Charette et al., 1991 | Control 1 | Untrained | 0 (6/0) | 67.7±4.1 | 58.8±4.1 | 163±9 | 1RM | Leg Press |  |  |  |  |  |  |  |
|  | Exercise 1 |  | 0 (13/0) | 69.8±4.3 | 59.2±6.85 | 162±7 |  |  | 3 | 6 | 7 | 70 | 4493 | 3 | 12 |
| Chtourou et al., 2012 | Control 1 | Untrained | 10 (0/0) | 22.9±1.3 | 72±8.8 | 180±5 | 1RM | Barbell Squat |  |  |  |  |  |  |  |
|  | Exercise 1 |  | 5 (0/0) |  |  |  |  |  | 4 | 5 | 3 | 100 | 5160 | 3 | 8 |
|  | Exercise 2 |  | 5 (0/0) |  |  |  |  |  | 4 | 5 | 3 | 100 | 5352 | 3 | 8 |
|  | Exercise 3 |  | 5 (0/0) |  |  |  |  |  | 4 | 5 | 3 | 100 | 4944 | 3 | 8 |
|  | Exercise 4 |  | 5 (0/0) |  |  |  |  |  | 4 | 5 | 3 | 100 | 5136 | 3 | 8 |
| Coetsee et al., 2015 | Control 1 | Untrained | 19 (0/0) | 22.9±1.3 | 76.8±13.7 | 168.7±7.9 | 10RM | Leg Press / Bench Press |  |  |  |  |  |  |  |
|  | Exercise 1 |  | 22 (0/0) |  | 73.3±15.5 | 167.8±7.8 |  |  | 3 | 10 | 7 | 80 | 5466 | 3 | 16 |
| Conceicao et al., 2013 | Control 1 | Untrained | 0 (10/0) | 53±5.7 | 64.25±5.03 | 160±5 | 1RM | Leg Press / Bench Press |  |  |  |  |  |  |  |
|  | Exercise 1 |  | 0 (10/0) | 53.4±3.95 | 64.58±9.22 | 157±6 |  |  | 3 | 9 | 9 |  |  | 3 | 16 |
| Cook et al., 2018 | Control 1 | Untrained | 3 (0/0) | 19.7±1.5 | 67±4.9 | 174±3 | 1RM | Knee Extension |  |  |  |  |  |  |  |
|  | Exercise 1 |  | 3 (0/0) | 19.3±1.2 | 74.5±9.8 | 173±2 |  |  | 3 | 10 | 2 | 70 | 1109 | 3 | 6 |
| Cormie et al., 2010 | Control 1 | Trained | 8 (0/0) | 23.4±4.4 | 77.5±8.1 | 179.3±6.7 | 1RM | Barbell Squat |  |  |  |  |  |  |  |
|  | Exercise 1 |  | 8 (0/0) |  | 82.2±13.7 |  |  |  | 3 | 4 | 1 | 80 |  | 3 | 10 |
| Correa et al., 2012 | Control 1 | Untrained | 0 (17/0) | 67±5 |  | 158.1±10.2 | 1RM | Knee Extension |  |  |  |  |  |  |  |
|  | Exercise 1 |  | 0 (41/0) |  |  |  |  |  | 3 | 16 | 3 |  |  | 2 | 6 |
|  | Exercise 2 |  | 0 (14/0) |  |  |  |  |  | 3 | 13 | 3 |  |  | 2 | 12 |
|  | Exercise 3 |  | 0 (13/0) |  |  |  |  |  | 3 | 13 | 3 |  |  | 2 | 12 |
| Correa et al., 2015 | Control 1 | Untrained | 0 (11/0) | 58.9±5.8 | 67.6±6.01 | 158.5±3.2 | 1RM | Knee Extension |  |  |  |  |  |  |  |
|  | Exercise 1 |  | 0 (12/0) |  | 65.4±10.8 |  |  |  | 3 | 15 | 8 |  |  | 3 | 11 |
|  | Exercise 2 |  | 0 (13/0) |  | 70.1±9.9 |  |  |  | 1 | 15 | 8 |  |  | 3 | 11 |
| Cunha et al., 2020 | Control 1 | Untrained | 0 (21/0) | 68.04±4.38 | 63.71±12.57 | 155.67±6.01 | 1RM | Knee Extension |  |  |  |  |  |  |  |
|  | Exercise 1 |  | 0 (21/0) | 70.09±5.95 | 68.37±15.31 | 156.46±7.68 |  |  | 1 | 13 | 8 |  |  | 3 | 12 |
|  | Exercise 2 |  | 0 (20/0) | 68.6±4.44 | 62.61±12.35 | 155.12±5.24 |  |  | 3 | 13 | 8 |  |  | 3 | 12 |
| de Lima et al., 2012 | Control 1 | Untrained | 0 (8/0) | 23.4±1.29 | 61.6±8.58 | 167±6 | 1RM | Leg Press / Bench Press |  |  |  |  |  |  |  |
|  | Exercise 1 |  | 0 (10/0) | 25.2±4.35 | 64.14±9.79 | 163±6 |  |  | 3 | 23 | 8 |  |  | 4 | 12 |
|  | Exercise 2 |  | 0 (10/0) | 27.4±2.8 | 61.72±7.82 | 164±7 |  |  | 3 | 23 | 8 |  |  | 4 | 12 |
| de Oliveira Júnior et al., 2021 | Control 1 | Untrained | 0 (14/0) | 59.8±9.43 | 61.21±17.98 | 160±4 | 1RM | Leg Press |  |  |  |  |  |  |  |
|  | Exercise 1 |  | 0 (13/0) | 58.53±8.03 | 67.65±13.78 | 159±6 |  |  | 2 | 10 | 4 |  |  | 3 | 12 |
|  | Exercise 2 |  | 0 (13/0) | 59.31±8.37 | 67.75±13.37 | 159±5 |  |  | 4 | 10 | 4 |  |  | 3 | 12 |
| de Souza et al., 2014 | Control 1 | Untrained | 7 (0/0) | 22.1±2.4 | 82.2±25 | 177±4.8 | 1RM | Leg Press |  |  |  |  |  |  |  |
|  | Exercise 1 |  | 11 (0/0) | 25.9±6.4 | 73.5±16.1 | 172±4.3 |  |  | 4 | 9 | 3 |  |  | 2 | 8 |
| Dobbs et al., 2018 | Control 1 | Untrained | 0 (7/0) | 62.71±9.46 | 67.96±12.28 | 166.5±5.44 | 3-5RM | Leg Press |  |  |  |  |  |  |  |
|  | Exercise 1 |  | 0 (0/8) | 66.25±7.09 | 80.12±16 | 165.44±6.6 |  |  | 3 | 10 | 3 | 73 |  | 3 | 8 |
| Donges et al., 2013 | Control 1 | Untrained | 8 (0/0) | 49.5±2.6 | 92.2±6.9 | 176.5±0.01 | 5RM | Leg Press / Chest Press |  |  |  |  |  |  |  |
|  | Exercise 1 |  | 13 (0/0) | 51.7±2.1 | 96.4±3.3 | 180.3±1.3 |  |  | 4 | 8 | 9 | 80 | 5270 | 3 | 12 |
| Dong-il et al., 2016 | Control 1 | Untrained | 0 (8/0) | 47.8±6.8 | 61.5±10.1 | 160.5±3.8 | 1RM | Knee Extension |  |  |  |  |  |  |  |
|  | Exercise 1 |  | 0 (9/0) | 55.3±6.2 | 54.7±8.6 | 155.7±4.1 |  |  | 3 | 15 | 4 | 40 | 1711 | 3 | 12 |
|  | Exercise 2 |  | 0 (9/0) | 52±2.8 | 56±4.7 | 158.2±2.7 |  |  | 3 | 15 | 4 | 70 | 3374 | 3 | 12 |
| Early et al., 2020 | Control 1 | Untrained | 0 (0/10) | 23±3 | 71.9±15.3 | 168±8 | 1RM | Knee Extension |  |  |  |  |  |  |  |
|  | Exercise 1 |  | 0 (0/10) |  | 79.6±15.9 | 170±10 |  |  | 3 | 10 | 6 | 60 | 3207 | 3 | 8 |
| Eidukaite et al., 2023 | Control 1 | Untrained | 2 (4/0) | 68.3±6.9 | 70.1±10.3 |  | Isokinetic Dynamometer | Knee Extension |  |  |  |  |  |  |  |
|  | Exercise 1 |  | 2 (4/0) | 72.3±2.6 | 67.5±9.4 |  |  |  | 3 | 7 | 4 | 78 | 211 | 2 | 12 |
| Elliott et al., 2002 | Control 1 | Untrained | 0 (7/0) | 49-62 | 59.99±7.69 |  | 10RM | Knee Extension / Bench Press |  |  |  |  |  |  |  |
|  | Exercise 1 |  | 0 (8/0) |  | 68.41±13.06 |  |  |  | 3 | 8 | 5 | 80 | 662 | 3 | 8 |
| Englund et al., 2019 | Control 1 | Untrained | 0 (0/35) | 80.3±6.3 | 77.1±14.9 | 170±10 | Isokinetic Dynamometer | Knee Extension |  |  |  |  |  |  |  |
|  | Exercise 1 |  | 0 (0/35) | 77.4±4.4 | 78±13.1 | 170±10 |  |  | 3 | 11 | 5 | 80 |  | 3 | 12 |
| Evangelista et al., 2019 | Control 1 | Untrained | 0 (0/16) | 27.1±4.1 | 78.8±12.9 | 177±8 | 1RM | Barbell Squat |  |  |  |  |  |  |  |
|  | Exercise 1 |  | 0 (0/21) | 25.1±3.2 | 78.1±15.3 | 175±7 |  |  | 3 | 10 | 3 |  |  | 2 | 8 |
| Eyre et al., 2024 | Control 1 | Untrained | 7 (0/0) | 22±3 | 76.7±7.1 | 182±4 | 1RM | Knee Extension |  |  |  |  |  |  |  |
|  | Exercise 1 |  | 15 (0/0) | 25±6 | 82.8±12.7 | 183±7 |  |  | 5 | 4 | 1 |  |  | 3 | 8 |
|  | Exercise 2 |  | 16 (0/0) | 24±5 | 79.6±8.3 | 182±6 |  |  | 5 | 4 | 1 |  |  | 3 | 8 |
| Farinatti et al., 2013 | Control 1 | Untrained | 0 (10/0) | 68±4 | 58.3±10.7 | 150±1 | 10RM | Knee Extension / Bench Press |  |  |  |  |  |  |  |
|  | Exercise 1 |  | 0 (10/0) | 72±5 | 56.1±15 | 149±3 |  |  | 1 | 10 | 10 | 70 | 515 | 1 | 16 |
|  | Exercise 2 |  | 0 (11/0) | 66±7 | 63.5±15.7 | 153±5 |  |  | 1 | 10 | 10 | 70 | 1107 | 2 | 16 |
|  | Exercise 3 |  | 0 (10/0) | 68±4 | 62.2±10.1 | 154±5 |  |  | 1 | 10 | 10 | 70 | 1720 | 3 | 16 |
| Fatouros et al., 2006 | Control 1 | Untrained | 10 (0/0) | 69.8±5.1 | 80.3±9.8 | 165±90 | 1RM | Leg Press / Chest Press |  |  |  |  |  |  |  |
|  | Exercise 1 |  | 14 (0/0) | 71.1±3.6 | 81.2±12 | 163±120 |  |  | 3 | 15 | 10 | 48 | 2313 | 3 | 16 |
|  | Exercise 2 |  | 12 (0/0) | 69.7±3.8 | 80.1±9.2 | 165±160 |  |  | 3 | 10 | 10 | 63 | 3063 | 3 | 16 |
|  | Exercise 3 |  | 14 (0/0) | 70.8±2.8 | 82±10.1 | 167±110 |  |  | 3 | 8 | 10 | 83 | 3996 | 3 | 16 |
| Fatouros et al., 2002 | Control 1 | Untrained | 8 (0/0) | 70.5±5.9 | 81.2±10.3 | 166±50 | 1RM | Leg Press / Chest Press |  |  |  |  |  |  |  |
|  | Exercise 1 |  | 8 (0/0) | 70.3±2.3 | 80.3±9.4 | 166±80 |  |  | 3 | 10 | 10 | 70 | 3279 | 3 | 16 |
| Fatouros et al., 2005 | Control 1 | Untrained | 14 (0/0) | 71.2±4.1 | 81.3±4.9 | 164±80 | 1RM | Leg Press / Chest Press |  |  |  |  |  |  |  |
|  | Exercise 1 |  | 18 (0/0) | 70.3±4.4 | 81.9±5.1 | 163±130 |  |  | 3 | 15 | 10 | 53 | 3098 | 3 | 24 |
|  | Exercise 2 |  | 20 (0/0) | 72.4±3.5 | 82.1±5.1 | 165±90 |  |  | 3 | 7 | 10 | 83 | 5044 | 3 | 24 |
| Fernandez-Lezaun et al., 2017 | Control 1 | Untrained | 11 (0/0) | 70±2 | 83±7 | 174±4 | 1RM | Leg Press |  |  |  |  |  |  |  |
|  | Exercise 1 |  | 11 (0/0) | 70±2 | 85±13 | 174±5 |  |  | 3 | 10 | 7 | 68 | 3953 | 1 | 39 |
|  | Exercise 2 |  | 7 (0/0) | 71±4 | 91±15 | 176±3 |  |  | 3 | 10 | 6 | 68 | 8439 | 2 | 39 |
|  | Exercise 3 |  | 11 (0/0) | 70±3 | 91±14 | 176±6 |  |  | 3 | 10 | 6 | 68 | 12253 | 3 | 39 |
|  | Control 2 |  | 0 (9/0) | 69±2 | 65±9 | 160±4 |  |  |  |  |  |  |  |  |  |
|  | Exercise 4 |  | 0 (12/0) | 70±3 | 67±9 | 160±6 |  |  | 3 | 10 | 7 | 68 | 2769 | 1 | 39 |
|  | Exercise 5 |  | 0 (14/0) | 68±3 | 74±13 | 163±4 |  |  | 3 | 10 | 6 | 68 | 6016 | 2 | 39 |
|  | Exercise 6 |  | 0 (13/0) | 69±3 | 70±9 | 160±5 |  |  | 3 | 10 | 6 | 68 | 8949 | 3 | 39 |
| Ferraresi et al., 2011 | Control 1 | Untrained | 10 (0/0) | 21.8±2.1 | 77.1±13.5 | 180±5 | 1RM | Leg Press |  |  |  |  |  |  |  |
|  | Exercise 1 |  | 10 (0/0) | 21.2±2.5 | 75.7±6.3 | 178±5 |  |  | 5 | 10 | 1 | 80 |  | 2 | 12 |
| Filho et al., 2022 | Control 1 | Untrained | 0 (19/0) | 68±5 | 67.88±11.32 | 156±6 | 10RM | Chest Press / Leg Press |  |  |  |  |  |  |  |
|  | Control 2 |  |  |  |  |  |  |  |  |  |  |  |  |  |  |
|  | Exercise 1 |  | 0 (20/0) | 65±4 | 70±14.09 | 154±5 |  |  | 3 | 13 | 5 | 60 | 1061 | 2 | 8 |
|  | Exercise 2 |  |  |  |  |  |  |  | 1 | 23 | 5 |  |  | 2 | 12 |
|  | Exercise 3 |  | 0 (18/0) | 66±4 | 71±14.58 | 157±5 |  |  | 3 | 13 | 5 | 60 | 1039 | 2 | 8 |
|  | Exercise 4 |  |  |  |  |  |  |  | 3 | 10 | 5 | 50 | 1855 | 2 | 12 |
|  | Exercise 5 |  | 0 (17/0) | 67±4 | 60.59±10.26 | 155±7 |  |  | 3 | 13 | 5 | 60 | 951 | 2 | 8 |
|  | Exercise 6 |  |  |  |  |  |  |  | 3 | 10 | 5 |  |  | 2 | 12 |
|  | Exercise 7 |  | 0 (21/0) | 66±5 | 73.91±13.07 | 157±5 |  |  | 3 | 13 | 5 | 60 | 1129 | 2 | 8 |
|  | Exercise 8 |  |  |  |  |  |  |  | 5 | 5 | 5 |  |  | 2 | 12 |
| Fimland et al., 2009 | Control 1 | Trained | 9 (0/0) | 24±2 | 77±7 | 183±6 | 1RM | Leg Press |  |  |  |  |  |  |  |
|  | Exercise 1 |  | 10 (0/0) | 23±3 | 80±7 | 184±6 |  |  | 4 | 4 | 1 | 88 | 3485 | 3 | 8 |
| Fisher et al., 2013 | Control 1 | Trained | 12 (0/0) | 24.5±7.5 | 84.4±11 | 179.3±4.5 | 1RM | Deadlift |  |  |  |  |  |  |  |
|  | Exercise 1 |  | 12 (0/0) | 26.5±7 | 82.1±8.3 | 178.4±6.8 |  |  | 1 | 10 | 1 | 80 | 1131 | 1 | 10 |
| Fragala et al., 2014 | Control 1 | Untrained | 5 (0/0) | 68.4±4.3 | 89±19.8 |  | < 10RM | Knee Extension |  |  |  |  |  |  |  |
|  | Control 2 |  | 0 (6/0) | 70.7±6.6 | 69.6±11.1 |  |  |  |  |  |  |  |  |  |  |
|  | Exercise 1 |  | 5 (0/0) | 67.3±5 | 88±21.2 |  |  |  | 3 | 12 | 8 | 78 |  | 2 | 6 |
|  | Exercise 2 |  | 0 (5/0) | 74.4±7.5 | 73.1±11 |  |  |  | 3 | 12 | 8 | 78 |  | 2 | 6 |
| Gerage et al., 2013 | Control 1 | Untrained | 0 (14/0) | 66.2±4.1 | 61.1±11.7 | 157.5±7.1 | 1RM | Knee Extension / Bench Press |  |  |  |  |  |  |  |
|  | Exercise 1 |  | 0 (15/0) | 65.5±5 | 57.3±6.5 | 156.7±5.1 |  |  | 2 | 13 | 8 |  |  | 3 | 12 |
| Gomes et al., 2017 | Control 1 | Untrained | 0 (10/0) | 68.8±5.41 | 67.19±7.2 | 150±253 | 1RM | Leg Press / Bench Press |  |  |  |  |  |  |  |
|  | Exercise 1 |  | 0 (10/0) | 66.1±2.77 | 66.14±13.71 | 154±209 |  |  | 3 | 9 | 8 | 80 | 2329 | 3 | 12 |
| Goto et al., 2005 | Control 1 | Trained | 8 (0/0) | 23.3±0.9 | 66.3±2.2 | 174.3±1.2 | 1RM | Knee Extension |  |  |  |  |  |  |  |
|  | Exercise 1 |  | 9 (0/0) | 23.1±0.9 | 65.2±3 | 171.7±2.3 |  |  | 4 | 10 | 3 | 75 | 2793 | 2 | 12 |
|  | Exercise 2 |  | 9 (0/0) | 21.9±0.7 | 66.4±2.4 | 170.3±1.7 |  |  | 4 | 10 | 3 | 75 | 2661 | 2 | 12 |
| Griffen et al., 2022 | Control 1 | Untrained | 9 (0/0) | 67±6 | 79±10.2 | 177±3 | 1RM | Leg Press |  |  |  |  |  |  |  |
|  | Exercise 1 |  | 9 (0/0) | 67±3 | 78.2±11.7 | 177±6 |  |  | 3 | 8 | 6 | 80 | 3053 | 2 | 12 |
| Gualano et al., 2014 | Control 1 | Untrained | 0 (15/0) | 66.3±6 | 63.4±8.8 | 155±7 | 1RM | Bench Press / Leg Press |  |  |  |  |  |  |  |
|  | Exercise 1 |  | 0 (15/0) | 63.6±3.6 | 66.8±9 | 154±4 |  |  | 3 | 10 | 6 |  |  | 2 | 24 |
| Hagerman et al., 2000 | Control 1 | Untrained | 9 (0/0) | 66.2±6.5 | 80.2±4.5 | 178.5±5.8 | 1RM | Leg Press |  |  |  |  |  |  |  |
|  | Exercise 1 |  | 9 (0/0) | 63.7±5 | 83.8±17.6 | 178.2±8.5 |  |  | 4 | 7 | 3 | 88 | 9151 | 2 | 16 |
| Harber et al., 2004 | Control 1 | Untrained | 4 (0/0) | 20.5±1 | 74.2±23.6 |  | 1RM | Leg Press / Chest Press |  |  |  |  |  |  |  |
|  | Exercise 1 |  | 8 (0/0) | 23.6±1.8 | 80.9±24.33 |  |  |  | 2 | 12 | 10 | 50 | 11720 | 3 | 10 |
| Haykowsky et al., 2000 | Control 1 | Untrained | 10 (0/0) | 68±4 |  |  | 1RM | Leg Press / Bench Press |  |  |  |  |  |  |  |
|  | Exercise 1 |  | 10 (0/0) | 68±3 |  |  |  |  |  | 7 | 8 | 70 | 12931 | 3 | 16 |
| Hendrickson et al., 2010 | Control 1 | Untrained | 0 (10/0) | 20±0.5 | 65.7±2.6 | 164.8±1.8 | 1RM | Bench Press / Squat |  |  |  |  |  |  |  |
|  | Exercise 1 |  | 0 (17/0) | 21±0.5 | 64.5±1.9 | 164.7±1.9 |  |  | 3 | 8 | 6 |  |  | 3 | 8 |
| Henwood et al., 2006 | Control 1 | Untrained | 0 (0/22) | 69.1±3.6 | 72.3±11.1 | 168.8±7.6 | 1RM | Leg Press / Chest Press |  |  |  |  |  |  |  |
|  | Exercise 1 |  | 0 (0/23) | 70.7±5.5 | 73±12.6 | 164.1±7.9 |  |  | 3 | 8 | 6 | 60 | 3216 | 2 | 8 |
|  | Exercise 2 |  | 0 (0/22) | 70.2±5 | 76.7±10.9 | 167.9±7.5 |  |  | 3 | 8 | 6 | 75 | 4267 | 2 | 8 |
| Henwood et al., 2008 | Control 1 | Untrained | 0 (0/15) | 69.3±3.87 | 71.9±11.23 | 167.9±8.13 | 1RM | Leg Press / Chest Press |  |  |  |  |  |  |  |
|  | Exercise 1 |  | 0 (0/19) | 71.2±5.67 | 71.7±8.72 | 163.3±7.41 |  |  | 3 | 8 | 6 | 60 | 2934 | 2 | 24 |
|  | Exercise 2 |  | 0 (0/19) | 69.6±4.8 | 76.5±10.9 | 167.6±7.85 |  |  | 3 | 8 | 6 | 75 | 3602 | 2 | 24 |
| Herda et al., 2021 | Control 1 | Untrained | 0 (0/13) | 67.3±6.85 | 69±14.98 | 169.1±7.6 | 1) 5RM; 2) 12RM | 1) Leg Press; 2) Bench Press |  |  |  |  |  |  |  |
|  | Exercise 1 |  | 0 (0/21) |  | 74.48±12.77 |  |  |  | 3 | 10 | 6 |  |  | 2 | 6 |
| Holviala et al., 2012 | Control 1 | Untrained | 21 (0/0) | 56.7±9.9 | 77.5±10.4 | 175.3±6.5 | 1RM | Leg Press |  |  |  |  |  |  |  |
|  | Exercise 1 |  | 30 (0/0) | 56.5±7.6 | 83.9±10.1 | 178.1±6.2 |  |  |  |  | 10 | 70 | 4568 | 2 | 21 |
| Holviala et al., 2014 | Control 1 | Untrained | 0 (20/0) | 27±3 | 69±8 | 161±5 | 1RM | Leg Press |  |  |  |  |  |  |  |
|  | Control 2 |  | 17 (0/0) | 25±3 | 78±12 | 175±7 |  |  |  |  |  |  |  |  |  |
|  | Exercise 1 |  | 0 (48/0) |  | 68±10 | 164±6 |  |  | 2 | 11 | 11 | 60 | 2657 | 2 | 21 |
|  | Exercise 2 |  | 41 (0/0) |  | 77±9 | 177±6 |  |  | 2 | 11 | 11 | 60 | 3958 | 2 | 21 |
| Hooshmand-Moghadam et al., 2020 | Control 1 | Untrained | 15 (0/0) | 66.1±3 | 61.9±3.8 | 168.3±3.3 | 1RM | Chest Press / Leg Press |  |  |  |  |  |  |  |
|  | Exercise 1 |  | 15 (0/0) | 66.3±3.4 | 64.9±3.7 | 167.9±3.6 |  |  | 4 | 15 | 6 | 60 | 2074 | 3 | 12 |
| Huschtscha et al., 2021 | Control 1 | Untrained | 0 (0/10) | 56.1±6.36 | 67.8±6.43 | 160±7.01 | 1RM | Leg Press / Chest Press |  |  |  |  |  |  |  |
|  | Exercise 1 |  | 0 (0/10) | 58±9.79 | 70±17.54 | 170±7.01 |  |  | 3 | 9 | 10 | 80 | 9377 | 3 | 12 |
| Jeong et al., 2020 | Control 1 | Untrained | 0 (13/0) | 22±2 | 53.7±4.7 | 162.9±3.4 | Isokinetic Dynamometer | Knee Extension |  |  |  |  |  |  |  |
|  | Exercise 1 |  | 0 (12/0) | 24±2 | 54.6±5.4 | 162±4.6 |  |  | 3 | 16 | 8 | 50 |  | 3 | 10 |
| Kalapotharakos et al., 2004 | Control 1 | Untrained | 0 (0/10) | 64.4±3.4 | 75±11.6 | 159±8 | 1RM | Knee Extension / Chest Press |  |  |  |  |  |  |  |
|  | Exercise 1 |  | 0 (0/11) | 64.6±5.1 | 78.6±17.4 | 163±7 |  |  | 3 | 8 | 8 | 80 | 3422 | 3 | 12 |
|  | Exercise 2 |  | 0 (0/12) | 65.7±4.2 | 78.7±10.6 | 159±7 |  |  | 3 | 15 | 8 | 60 | 2596 | 3 | 12 |
| Kalapotharakos et al., 2007 | Control 1 | Untrained | 9 (0/0) | 67.5±4.8 | 78.6±5.3 | 170±4 | 1RM | Knee Extension |  |  |  |  |  |  |  |
|  | Exercise 1 |  | 9 (0/0) | 68.4±5.5 | 81.1±4.9 | 173±3.7 |  |  | 3 | 15 | 6 | 60 | 1080 | 3 | 10 |
| Karabulut et al., 2010 | Control 1 | Untrained | 11 (0/0) | 57±3.65 | 82.4±15.26 | 174.1±8.29 | 1RM | Leg Press |  |  |  |  |  |  |  |
|  | Exercise 1 |  | 13 (0/0) | 57.5±2.88 | 85.1±16.25 | 177.3±6.13 |  |  | 3 | 8 | 5 | 80 | 6611 | 3 | 6 |
| Katsoulis & Amara 2023 | Control 1 | Untrained | 0 (15/0) | 73.3±3.6 | 67.5±12.1 | 160.1±8.2 | 1RM | Leg Press |  |  |  |  |  |  |  |
|  | Exercise 1 |  | 0 (14/0) | 74.3±4.9 | 61.9±10.9 | 150±6.7 |  |  | 3 | 12 | 5 | 40 | 277 | 1 | 12 |
|  | Exercise 2 |  | 0 (17/0) | 74.4±4.5 | 66.7±10.9 | 159.2±6.7 |  |  | 3 | 12 | 5 | 40 | 626 | 2 | 12 |
|  | Exercise 3 |  | 0 (17/0) | 74.8±4.9 | 68.5±14.3 | 158.9±6.3 |  |  | 3 | 12 | 5 | 40 | 791 | 3 | 12 |
| Kell., 2011 | Control 1 | Trained | 0 (0/20) | 23.1±4.8 | 70.1±4.7 | 174±5 | 10RM | Barbell Squat / Bench Press |  |  |  |  |  |  |  |
|  | Exercise 1 |  | 20 (0/0) | 22.7±4.1 | 78.2±4.1 | 178±5 |  |  | 4 | 9 | 9 | 64 |  | 4 | 12 |
|  | Exercise 2 |  | 0 (20/0) | 22.5±4.6 | 59.4±5 | 170±6 |  |  | 4 | 9 | 9 | 64 |  | 4 | 12 |
| Kim et al., 2011 | Control 1 | Untrained | 0 (8/0) | 21.5±2.26 | 62.6±9.33 | 167.6±5.93 | 1RM | Leg Press / Chest Press |  |  |  |  |  |  |  |
|  | Exercise 1 |  | 0 (13/0) | 20.8±2.88 | 72.3±12.98 | 170.3±7.93 |  |  | 3 | 8 | 5 | 80 | 2515 | 3 | 4 |
| Kirk et al., 2007 | Control 1 | Untrained | 8 (0/0) | 20±3 | 89.2±9.8 | 178±5.8 | 1RM | Leg Press / Chest Press |  |  |  |  |  |  |  |
|  | Exercise 1 |  | 11 (0/0) | 21±2 | 91.7±11.1 | 180±7 |  |  | 1 | 5 | 9 | 88 | 16223 | 3 | 24 |
| Kraemer et al., 2004 | Control 1 | Untrained | 0 (6/0) | 23.1±2.4 | 65.9±11.2 | 164.5±6.2 | 1RM | Barbell Squat / Bench Press |  |  |  |  |  |  |  |
|  | Exercise 1 |  | 0 (18/0) | 22.4±3.5 | 64.1±8.8 | 163.7±7.5 |  |  | 3 | 8 | 8 |  |  | 3 | 24 |
|  | Exercise 2 |  | 0 (21/0) | 23.8±3.7 | 63.2±7 | 165.4±5.4 |  |  | 3 | 10 | 9 |  |  | 3 | 24 |
|  | Exercise 3 |  | 0 (21/0) | 22.7±4 | 66.7±10.7 | 165.5±7.2 |  |  | 3 | 8 | 8 |  |  | 3 | 24 |
|  | Exercise 4 |  | 0 (19/0) | 23.5±3.7 | 65.7±12.1 | 166.7±6.2 |  |  | 3 | 10 | 8 |  |  | 3 | 24 |
| Lamas et al., 2012 | Control 1 | Trained | 7 (0/0) | 24.1±2.7 | 74.1±9.6 | 178.9±11 | 1RM | Barbell Squat |  |  |  |  |  |  |  |
|  | Exercise 1 |  | 11 (0/0) | 22.5±3.8 | 75.2±7.3 | 178.7±4.3 |  |  | 6 | 7 | 1 | 81 | 2825 | 3 | 8 |
|  | Exercise 2 |  | 11 (0/0) | 24.2±4.1 | 76±8.9 | 177±5.9 |  |  | 6 | 7 | 1 | 45 | 1590 | 3 | 8 |
| Lanza et al., 2024 | Control 1 | Untrained | 11 (0/0) | 21.8±5 | 79.2±12.2 | 178±8 | 1RM | Bench Press |  |  |  |  |  |  |  |
|  | Exercise 1 |  | 13 (0/0) | 24.3±4.4 | 73.4±10.7 | 175±5 |  |  | 4 | 12 | 1 | 53 | 909 | 3 | 10 |
| Lee et al., 2022 | Control 1 | Untrained | 0 (0/8) | 66.5±5 | 68.9±15.8 | 167.2±10.2 | 1RM | Leg Press |  |  |  |  |  |  |  |
|  | Exercise 1 |  | 0 (0/10) | 66.6±7.3 | 66.5±11.5 | 167.9±5.7 |  |  | 3 | 10 | 5 | 70 | 5967 | 2 | 12 |
| Leite et al., 2015 | Control 1 | Trained | 0 (7/0) | 46±6.52 | 56.8±5.02 | 162±5.58 | 10RM | Leg Press / Bench Press |  |  |  |  |  |  |  |
|  | Exercise 1 |  | 0 (7/0) |  |  |  |  |  | 3 | 10 | 8 |  |  | 3 | 12 |
| Letieri et al., 2018 | Control 1 | Untrained | 0 (12/0) | 69±6.39 | 67.24±5.28 | 150±3 | Isokinetic Dynamometer | Unilateral Knee Extension |  |  |  |  |  |  |  |
|  | Exercise 1 |  | 0 (10/0) | 66.75±4.43 | 69.5±11.73 | 152±4 |  |  | 4 | 7 | 4 | 75 |  | 3 | 16 |
|  | Exercise 2 |  | 0 (12/0) | 71.27±4.73 | 65.17±8.32 | 151±5 |  |  | 4 | 15 | 4 | 25 |  | 3 | 16 |
| Libardi et al., 2012 | Control 1 | Untrained | 13 (0/0) | 49.1±5.78 | 73.3±13.31 | 172±7 | 1RM | Leg Press / Bench Press |  |  |  |  |  |  |  |
|  | Exercise 1 |  | 11 (0/0) | 49.27±4.81 | 81.51±15.14 | 172±6 |  |  | 3 | 9 | 8 |  |  | 3 | 16 |
| Libardi et al., 2011 | Control 1 | Untrained | 13 (0/0) | 49.1±5.5 | 73.3±13.3 | 170±10 | 1RM | Leg Press / Bench Press |  |  |  |  |  |  |  |
|  | Exercise 1 |  | 12 (0/0) | 48.6±5 | 82.8±15.1 | 170 |  |  | 3 | 9 | 9 |  |  | 3 | 16 |
| Lixandrao et al., 2016 | Control 1 | Untrained | 0 (0/8) | 65.7±4.6 | 69.2±16.1 | 161.1±10.1 | 1RM | Leg Press |  |  |  |  |  |  |  |
|  | Exercise 1 |  | 0 (0/6) | 60.3±2.7 | 68.5±14.3 | 163.3±9 |  |  | 4 | 10 | 1 | 75 | 3325 | 2 | 10 |
| Lo et al., 2011 | Control 1 | Untrained | 10 (0/0) | 21.1±1.66 | 67.2±10.66 | 173±5.56 | 1RM | Knee Extension / Chest Press |  |  |  |  |  |  |  |
|  | Exercise 1 |  | 10 (0/0) | 20.2±1.4 | 65±8.91 | 170.7±4.44 |  |  | 2 | 10 | 10 | 75 | 6471 | 3 | 24 |
| Lopes et al., 2016 | Control 1 | Untrained | 0 (11/0) | 65±3.1 | 70.9±3 | 154±5.6 | 1RM | Leg Press |  |  |  |  |  |  |  |
|  | Exercise 1 |  | 0 (14/0) | 69±7.3 | 72±9.7 | 155±5.6 |  |  | 3 | 8 | 7 |  |  | 3 | 12 |
|  | Exercise 2 |  | 0 (12/0) | 67±7.4 | 67.2±7 | 153±5.5 |  |  | 4 | 7 | 7 | 61 | 3774 | 3 | 12 |
| Manocchia et al., 2013 | Control 1 | Trained | 0 (0/14) | 39.6±15.8 | 76.8±15.8 |  | 1) 3RM; 2) 12RM | 1) Clean and Jerk; 2) Bench Press |  |  |  |  |  |  |  |
|  | Exercise 1 |  | 0 (0/23) | 40.8±12.9 | 76.6±14.4 |  |  |  | 3 | 13 | 7 |  |  | 2 | 10 |
| Martín-Hernández et al., 2013 | Control 1 | Untrained | 8 (0/0) | 20.2±0.8 | 75.6±6.4 | 178.4±5.3 | 1RM | Knee Extension |  |  |  |  |  |  |  |
|  | Exercise 1 |  | 11 (0/0) | 20.7±2.3 | 75.2±10.5 | 180.1±5.8 |  |  | 3 | 8 | 1 | 85 | 1257 | 2 | 5 |
| Marx et al., 2001 | Control 1 | Untrained | 0 (10/0) | 22.2±5.7 | 55.6±5.7 | 165.7±5.7 | 1RM | Leg Press / Bench Press |  |  |  |  |  |  |  |
|  | Exercise 1 |  | 0 (12/0) | 23.2±4.5 | 55.4±6.5 | 166.1±5.4 |  |  | 1 | 10 | 10 |  |  | 3 | 24 |
|  | Exercise 2 |  | 0 (12/0) | 22.6±3.7 | 65.7±5.7 | 165.4±4.4 |  |  | 3 | 9 | 10 |  |  | 4 | 24 |
| Maté-Muñoz et al., 2014 | Control 1 | Untrained | 12 (0/0) | 22.3±2.4 | 75.4±9.9 | 176±7 | 1RM | Barbell Squat / Bench Press |  |  |  |  |  |  |  |
|  | Exercise 1 |  | 10 (0/0) | 21.8±1.1 | 71.8±6.5 | 178±5 |  |  | 3 | 15 | 8 |  |  | 3 | 7 |
| McBride et al., 2003 | Control 1 | Untrained | 0 (0/10) | 22.43±1.89 | 70.55±7.77 | 171.3±7.21 | 1RM | Leg Press |  |  |  |  |  |  |  |
|  | Exercise 1 |  | 0 (0/9) | 22.09±3.43 | 83.74±29.44 | 172.83±10.47 |  |  | 1 | 8 | 4 |  |  | 2 | 12 |
|  | Exercise 2 |  | 0 (0/9) | 20.04±1.22 | 70.68±23.03 | 169.44±11.79 |  |  | 6 | 8 | 4 |  |  | 2 | 12 |
| McCartney et al., 1995 | Control 1 | Untrained | 0 (19/0) | 65±2.8 | 67.1±9.3 | 162.6±5.5 | 1RM | Leg Press / Bench Press |  |  |  |  |  |  |  |
|  | Control 2 |  | 0 (23/0) | 73±3.5 | 60±6.8 | 157.4±5.1 |  |  |  |  |  |  |  |  |  |
|  | Exercise 1 |  | 0 (17/0) | 65±2.4 | 62.5±9.5 | 160.8±5.9 |  |  | 3 | 11 | 7 | 65 | 9209 | 2 | 42 |
|  | Exercise 2 |  | 0 (20/0) | 71±1.8 | 67.1±8.6 | 162.2±5.7 |  |  | 3 | 11 | 7 | 65 | 8434 | 2 | 42 |
|  | Control 3 |  | 16 (0/0) | 63±2.4 | 83.1±10.3 | 177.1±6.4 |  |  |  |  |  |  |  |  |  |
|  | Control 4 |  | 8 (0/0) | 72±2.8 | 82.9±10.1 | 177±3.2 |  |  |  |  |  |  |  |  |  |
|  | Exercise 3 |  | 22 (0/0) | 64±2.4 | 81.3±8.5 | 175.2±5.8 |  |  | 3 | 11 | 7 | 65 | 14666 | 2 | 42 |
|  | Exercise 4 |  | 17 (0/0) | 73±3.1 | 75.9±6.6 | 173.5±4.7 |  |  | 3 | 11 | 7 | 65 | 14197 | 2 | 42 |
| McGinley et al., 2007 | Control 1 | Untrained | 9 (0/0) | 25.2±4.4 | 78.7±8.5 | 180.1±7.1 | 12RM | Chest Press |  |  |  |  |  |  |  |
|  | Exercise 1 |  | 12 (0/0) | 26.9±4.8 | 82.1±13.3 | 180.7±6.7 |  |  | 2 | 10 | 5 | 65 | 2293 | 2 | 8 |
| Mohammad Ali et al., 2014 | Control 1 | Untrained | 10 (0/0) | 22.9±1.66 | 69.89±5.93 | 173.8±10.32 | 1RM | Leg Press / Bench Press |  |  |  |  |  |  |  |
|  | Exercise 1 |  | 10 (0/0) | 23.1±1.37 | 67.12±5.61 | 167.8±4.49 |  |  | 3 | 10 | 9 | 70 | 4821 | 3 | 12 |
| Morgan et al., 2023 | Control 1 | Untrained | 0 (8/0) | 20.9±1.8 | 63.8±13 | 162.6±6.2 | 1RM | Bench Press / Barbell Squat |  |  |  |  |  |  |  |
|  | Exercise 1 |  | 0 (8/0) | 20.8±1.2 | 66.8±8.4 | 160.8±2 |  |  | 3 | 6 | 9 | 85 | 1326 | 5 | 11 |
|  | Exercise 2 |  | 0 (8/0) | 19.8±1.7 | 60.1±10.1 | 163.8±3.9 |  |  | 4 | 13 | 9 | 55 | 1381 | 5 | 11 |
| Mosti et al., 2014 | Control 1 | Untrained | 0 (15/0) | 21.5±2.2 | 66.1±6.3 | 168.1±6.8 | 1RM | Leg Press |  |  |  |  |  |  |  |
|  | Exercise 1 |  | 0 (15/0) | 22.7±2.2 | 64.5±12.2 | 168.3±7.1 |  |  | 4 | 4 | 1 | 88 | 2230 | 3 | 12 |
| Nascimento et al., 2022 | Control 1 | Untrained | 0 (14/0) | 59.8±9.43 | 61.21±17.98 | 160±4 | 1RM | Leg Press |  |  |  |  |  |  |  |
|  | Exercise 1 |  | 0 (13/0) | 59.31±8.37 | 67.75±13.37 | 159±5 |  |  | 6 | 10 | 4 | 80 | 4518 | 3 | 12 |
|  | Exercise 2 |  | 0 (13/0) | 58.53±8.03 | 67.65±13.78 | 159±6 |  |  | 3 | 10 | 4 | 80 | 5221 | 3 | 12 |
| Nichols et al., 1995 | Control 1 | Untrained | 0 (0/20) | 67±5.6 | 72.8±13.2 | 165.6±9.23 | 1RM | Leg Press / Chest Press |  |  |  |  |  |  |  |
|  | Exercise 1 |  | 0 (0/20) | 67.8±4.9 | 73.1±13.4 | 168±8.7 |  |  | 3 | 12 | 6 | 50 | 2070 | 2 | 12 |
| Nichols et al., 1993 | Control 1 | Untrained | 0 (15/0) | 65.3±5.42 | 69.6±11.62 | 164.3±5.81 | 1RM | Knee Extension / Bench Press |  |  |  |  |  |  |  |
|  | Exercise 1 |  | 0 (15/0) | 67.8±6.19 | 68.8±10.84 | 162.8±5.04 |  |  | 3 | 9 | 7 | 80 | 13789 | 3 | 24 |
| Nunes et al., 2019 | Control 1 | Untrained | 0 (12/0) | 59±5.59 | 74±14.4 | 150 | 1RM | Knee Extension / Bench Press |  |  |  |  |  |  |  |
|  | Exercise 1 |  | 0 (10/0) | 64.2±8.04 | 64.1±10.21 | 150±7 |  |  | 3 | 10 | 8 | 70 | 9606 | 3 | 16 |
|  | Exercise 2 |  | 0 (12/0) | 59.7±5.98 | 66.7±13.14 | 150±8 |  |  | 6 | 10 | 8 | 70 | 10087 | 3 | 16 |
| Olson et al., 2006 | Control 1 | Untrained | 0 (15/0) | 38±7.75 | 75.5±9.29 | 170 | 1RM | Leg Press / Bench Press |  |  |  |  |  |  |  |
|  | Exercise 1 |  | 0 (15/0) | 38±3.87 | 75.5±12.39 |  |  |  | 3 | 9 | 9 |  |  | 2 | 52 |
| Panton et al., 2001 | Control 1 | Untrained | 0 (0/10) | 70±3 | 69.7±9.5 | 169±8 | 1RM | Knee Extension / Chest Press |  |  |  |  |  |  |  |
|  | Exercise 1 |  | 0 (0/11) | 67±7 | 74±10.9 | 167±9 |  |  | 3 | 8 | 10 | 70 |  | 3 | 12 |
| Pinto et al., 2014 | Control 1 | Untrained | 0 (17/0) | 66±8 | 68.3±12.1 | 159.1±9.2 | 1RM | Knee Extension |  |  |  |  |  |  |  |
|  | Exercise 1 |  | 0 (19/0) |  |  |  |  |  | 3 | 16 | 3 |  |  | 2 | 6 |
| Prestes et al., 2015 | Control 1 | Untrained | 0 (10/0) | 66.9±7.56 | 58.94±6.66 | 152±5 | 1RM | Leg Press / Bench Press |  |  |  |  |  |  |  |
|  | Exercise 1 |  | 0 (20/0) | 69.2±6.05 | 62.69±12.03 | 152±6 |  |  | 3 | 10 | 10 |  |  | 2 | 16 |
|  | Exercise 2 |  | 0 (19/0) | 65.52±4.72 | 66.71±9.5 | 152±5 |  |  | 3 | 10 | 10 |  |  | 2 | 16 |
| Pyka et al., 1994 | Control 1 | Untrained | 0 (0/14) | 68.8±5.24 | 66±8.61 | 165±8.61 | 1RM | Leg Press / Bench Press |  |  |  |  |  |  |  |
|  | Exercise 1 |  | 0 (0/8) | 67.2±3.98 | 69.6±13.27 | 163±8.96 |  |  | 3 | 8 | 12 | 75 | 64214 | 3 | 52 |
| Rabelo et al., 2004 | Control 1 | Untrained | 0 (20/0) | 63.65±7.17 | 66.1±7.3 | 158±4.8 | 1RM | Leg Press / Bench Press |  |  |  |  |  |  |  |
|  | Exercise 1 |  | 0 (21/0) | 66.48±4.09 | 63±9.3 | 152±7.3 |  |  | 3 | 8 | 8 | 50 | 3427 | 3 | 10 |
|  | Exercise 2 |  | 0 (20/0) | 63.9±3.78 | 63.4±10.2 | 155±5.4 |  |  | 3 | 8 | 8 | 80 | 6460 | 3 | 10 |
| Ramirez-Campillo et al., 2014 | Control 1 | Untrained | 0 (15/0) | 66.7±4.9 | 65.2±7.2 | 148.6±5 | 1RM | Leg Press / Bench Press |  |  |  |  |  |  |  |
|  | Exercise 1 |  | 0 (15/0) | 68.7±6.4 | 71.1±15.5 | 151.6±5.9 |  |  | 3 | 8 | 8 | 75 | 2627 | 3 | 12 |
| Rana et al., 2008 | Control 1 | Untrained | 0 (8/0) | 22.9±6.79 | 72.5±42.43 | 163.6±12.73 | 1RM | Barbell Squat |  |  |  |  |  |  |  |
|  | Exercise 1 |  | 0 (9/0) | 20.6±5.7 | 64.1±23.7 | 165.6±14.7 |  |  | 3 | 8 | 3 |  |  | 3 | 6 |
|  | Exercise 2 |  | 0 (7/0) | 22.3±10.32 | 64.7±21.96 | 161.9±21.96 |  |  | 3 | 25 | 3 |  |  | 3 | 6 |
|  | Exercise 3 |  | 0 (10/0) | 19.4±4.11 | 63.9±26.56 | 168±13.28 |  |  | 3 | 8 | 3 |  |  | 3 | 6 |
| Reis et al., 2012 | Control 1 | Untrained | 0 (24/0) | 53.86±5.11 | 73.31±12.81 | 156±6 | 1RM | Knee Extension |  |  |  |  |  |  |  |
|  | Exercise 1 |  | 0 (27/0) | 52.91±4.02 | 68.66±12.81 | 155±7 |  |  | 3 | 11 | 6 | 73 | 237 | 2 | 12 |
| Rhodes et al., 2000 | Control 1 | Untrained | 0 (18/0) | 68.2±3.5 | 61.7±12.9 | 159.3±4.5 | 1RM | Leg Press / Bench Press |  |  |  |  |  |  |  |
|  | Exercise 1 |  | 0 (20/0) | 68.8±3.2 | 68.4±12 | 160.9±5.5 |  |  | 3 | 8 | 6 | 75 | 19740 | 3 | 52 |
| Santos et al., 2018 | Control 1 | Trained | 15 (0/0) | 24.3±2.8 | 72.6±5.4 | 173±6 | 1RM | Leg Press / Bench Press |  |  |  |  |  |  |  |
|  | Exercise 1 |  | 15 (0/0) |  |  |  |  |  | 4 | 15 | 10 | 60 |  | 3 | 12 |
|  | Exercise 2 |  | 15 (0/0) |  |  |  |  |  | 4 | 15 | 10 | 60 |  | 3 | 12 |
|  | Exercise 3 |  | 15 (0/0) |  |  |  |  |  | 4 | 15 | 10 | 60 |  | 3 | 12 |
| Santos et al., 2010 | Control 1 | Untrained | 0 (8/0) | 25.4±2.4 | 54.1±3.5 | 160±4.1 | 12RM | Bench Press |  |  |  |  |  |  |  |
|  | Exercise 1 |  | 0 (8/0) | 26.8±1.6 | 55.1±3.3 | 161±2.7 |  |  | 3 | 11 | 8 |  |  | 3 | 8 |
|  | Exercise 2 |  | 0 (8/0) | 24±2.3 | 60.3±4.5 | 164.3±6 |  |  | 3 | 11 | 8 |  |  | 3 | 8 |
| Schlumberger et al., 2001 | Control 1 | Trained | 0 (9/0) | 25.3±9.3 | 63.6±25.2 | 168.3±13.2 | 1RM | Knee Extension / Chest Press |  |  |  |  |  |  |  |
|  | Exercise 1 |  | 0 (9/0) | 29.1±27.6 | 70.5±39.6 | 162.8±26.4 |  |  | 1 | 8 | 7 |  |  | 2 | 6 |
|  | Exercise 2 |  | 0 (9/0) | 24.4±8.7 | 62±13.2 | 168.6±8.7 |  |  | 3 | 8 | 7 |  |  | 2 | 6 |
| Schneider et al., 2003 | Control 1 | Untrained | 7 (0/0) | 32±6 | 86±7 | 179±4 | 1RM | Barbell Squat |  |  |  |  |  |  |  |
|  | Exercise 1 |  | 7 (0/0) | 33±7 | 78±10 | 180±9 |  |  | 3 | 10 | 3 | 73 | 14086 | 3 | 16 |
| Seo et al., 2023 | Control 1 | Untrained | 0 (13/0) | 50.8±5.8 | 69.9±7.8 | 159.2±4.7 | Isokinetic Dynamometer | Knee Extension |  |  |  |  |  |  |  |
|  | Exercise 1 |  | 0 (15/0) |  | 72.7±10.3 | 158.1±6 |  |  | 4 | 9 | 8 | 75 |  | 3 | 12 |
| Shabkhiz et al., 2020 | Control 1 | Untrained | 12 (0/0) | 72.08±5.33 | 74.16±12.2 |  | 1RM | Knee Extension |  |  |  |  |  |  |  |
|  | Exercise 1 |  | 12 (0/0) |  |  |  |  |  | 3 | 10 | 8 | 70 | 4505 | 3 | 12 |
| Sharman et al., 2001 | Control 1 | Untrained | 3 (0/0) | 62.7±4.6 | 81±8.6 | 173.7±2.2 | 1RM | Barbell Squat |  |  |  |  |  |  |  |
|  | Control 2 |  | 0 (3/0) | 62±3.5 | 73.7±8.6 | 168.2±5 |  |  |  |  |  |  |  |  |  |
|  | Exercise 1 |  | 7 (0/0) | 66.4±4.8 | 83.2±13.2 | 172.5±6.6 |  |  | 3 | 5 | 6 |  |  | 2 | 24 |
|  | Exercise 2 |  | 0 (7/0) | 64.6±3.6 | 72.3±9.9 | 162.4±6.3 |  |  | 3 | 5 | 6 |  |  | 2 | 24 |
| Shaw et al., 2009 | Control 1 | Untrained | 12 (0/0) | 25±2.4 | 80.3±12.8 | 179.3±2.4 | 10RM | Leg Press / Chest Press |  |  |  |  |  |  |  |
|  | Exercise 1 |  | 13 (0/0) | 25±3.5 | 69.1±8.5 | 175.5±5.6 |  |  | 3 | 15 | 8 | 60 | 184 | 3 | 16 |
| Sheoran et al. 2023 | Control 1 | Untrained | 18 (23/0) | 69.19±5.85 | 75.22±10.82 | 167.65±7.42 | Isokinetic Dynamometer | Knee Extension |  |  |  |  |  |  |  |
|  | Exercise 1 |  |  | 70.15±5.33 | 78.07±15.21 | 165.3±7.65 |  |  | 3 | 7 | 4 | 78 |  | 2 | 12 |
| Simao et al., 2011 | Control 1 | Untrained | 0 (20/0) | 34±2.1 | 63.4±2 | 160±4 | 10RM | Leg Press / Bench Press |  |  |  |  |  |  |  |
|  | Exercise 1 |  | 0 (20/0) | 35±2 | 60±2.4 | 162±3 |  |  | 3 | 10 | 8 |  |  | 3 | 16 |
| Simao et al., 2012 | Control 1 | Untrained | 9 (0/0) | 25.9±3.6 | 73.9±9.9 | 171.2±6.3 | 1RM | Bench Press |  |  |  |  |  |  |  |
|  | Exercise 1 |  | 11 (0/0) | 30.2±1.1 | 79.5±13.1 | 173.6±7.2 |  |  | 3 | 9 | 4 |  |  | 2 | 12 |
|  | Exercise 2 |  | 10 (0/0) | 29.8±1.9 | 79.9±10.6 | 172±6.8 |  |  | 3 | 9 | 4 |  |  | 2 | 12 |
| Simons et al., 2006 | Control 1 | Untrained | 0 (0/21) | 83.5±6.2 |  |  | 1RM | Leg Press / Bench Press |  |  |  |  |  |  |  |
|  | Exercise 1 |  | 0 (0/21) |  |  |  |  |  | 1 | 10 | 6 | 75 | 4268 | 2 | 16 |
| Snow-Harter et al., 1992 | Control 1 | Untrained | 0 (8/0) | 19.9±5.05 | 61.2±6.2 | 167±9.6 | 1RM | Bench Press |  |  |  |  |  |  |  |
|  | Exercise 1 |  | 0 (12/0) |  | 59.8±4.6 | 165±4.5 |  |  | 3 | 10 | 14 | 75 | 8677 | 3 | 32 |
| Spineti et al., 2010 | Control 1 | Untrained | 9 (0/0) | 25.8±3.6 | 73.9±9.9 | 171.1±6.3 | 1RM | Bench Press |  |  |  |  |  |  |  |
|  | Exercise 1 |  | 11 (0/0) | 29.7±1 | 79.4±13.1 | 173.6±7.2 |  |  | 3 | 9 | 4 |  |  | 2 | 12 |
|  | Exercise 2 |  | 10 (0/0) | 30.5±1.7 | 81.8±15.4 | 173±6.5 |  |  | 3 | 9 | 4 |  |  | 2 | 12 |
| Strandberg et al., 2015 | Control 1 | Untrained | 0 (21/0) | 68±1 | 67.1±8.2 | 165±4.6 | 1RM | Knee Extension |  |  |  |  |  |  |  |
|  | Exercise 1 |  | 0 (21/0) | 68±2 | 65.2±8 | 163±5.3 |  |  | 3 | 10 | 5 | 80 | 1889 | 2 | 24 |
| Taaffe et al., 1999 | Control 1 | Untrained | 0 (0/14) | 68.9±3.6 | 80.4±10.3 | 173.7±8.3 | 1RM | Leg Press / Bench Press |  |  |  |  |  |  |  |
|  | Exercise 1 |  | 0 (0/14) | 68.5±3.6 | 70.2±14.4 | 170.6±12.2 |  |  | 3 | 8 | 8 | 80 | 6568 | 1 | 24 |
|  | Exercise 2 |  | 0 (0/14) | 69.4±3 | 70.3±8.9 | 174.5±6.9 |  |  | 3 | 8 | 8 | 80 | 13682 | 2 | 24 |
|  | Exercise 3 |  | 0 (0/11) | 71±4.1 | 72.4±13 | 172.2±11.2 |  |  | 3 | 8 | 8 | 80 | 20028 | 3 | 24 |
| Tanimoto et al., 2008 | Control 1 | Untrained | 12 (0/0) | 19.8±0.7 | 64.2±4 | 174.3±7.2 | 1RM | Barbell Squat / Chest Press |  |  |  |  |  |  |  |
|  | Exercise 1 |  | 12 (0/0) | 19.6±0.6 | 62.5±4.8 | 174.1±5.5 |  |  | 3 | 8 | 5 | 58 | 5396 | 2 | 13 |
|  | Exercise 2 |  | 12 (0/0) | 19.5±0.5 | 63.8±4 | 174.8±4.3 |  |  | 3 | 8 | 5 | 85 | 7794 | 2 | 13 |
| Teixeira et al., 2019 | Control 1 | Untrained | 0 (10/0) | 55.8±3.9 | 72.5±8.5 | 158±7 | 1RM | Bench Press / Knee Extension |  |  |  |  |  |  |  |
|  | Exercise 1 |  | 0 (10/0) | 56±4.5 | 66.2±8.2 | 157±7 |  |  | 2 | failure | 8 | 67.5 |  | 3 | 16 |
| Terrazas et al., 2025 | Control 1 | Untrained | 0 (0/10) | 23±2.9 | 69.2±21 | 164±10 | 1RM | Bench Press / Leg Press |  |  |  |  |  |  |  |
|  | Exercise 1 |  | 0 (0/10) | 23.5±2.8 | 73±19.3 | 167±10 |  |  | 3 | 10 | 6 | 75 | 1405 | 2 | 8 |
| Terzis et al., 2008 | Control 1 | Untrained | 4 (0/0) | 21±1 | 81.1±2.2 | 181±1 | 1RM | Barbell Squat / Bench Press |  |  |  |  |  |  |  |
|  | Exercise 1 |  | 6 (0/0) |  | 83.4±2.7 |  |  |  | 3 | 6 | 9 |  |  | 3 | 14 |
| Tieland et al., 2015 | Control 1 | Untrained | 0 (65/0) | 79.5±8.06 | 74±12.91 | 166±8 | 1RM | Leg Press |  |  |  |  |  |  |  |
|  | Exercise 1 |  | 0 (62/0) | 78.4±7.87 | 78.5±14.17 |  |  |  | 3 | 11 | 6 | 63 | 5625 | 2 | 24 |
| Toien et al., 2018 | Control 1 | Untrained | 15 (0/0) | 23±4 | 79.7±9.1 | 183±7 | 1RM | Knee Extension |  |  |  |  |  |  |  |
|  | Exercise 1 |  | 19 (0/0) | 24±2 | 77.5±8.7 | 181±6 |  |  | 4 | 4 | 1 | 93 | 2952 | 3 | 8 |
| Toien et al., 2021 | Control 1 | Untrained | 8 (0/0) | 24±5 | 82.2±7.6 | 185±8 | 1RM | Leg Press |  |  |  |  |  |  |  |
|  | Exercise 1 |  | 8 (0/0) | 24±3 | 77.2±7.9 | 179±5 |  |  | 4 | 5 | 2 | 90 | 2938 | 3 | 8 |
| Tomeleri et al., 2020 | Control 1 | Untrained | 0 (15/0) | 68.6±5.1 | 66.2±11.2 | 158.8±6.1 | 1RM | Chest Press / Knee Extension |  |  |  |  |  |  |  |
|  | Exercise 1 |  | 0 (15/0) | 71.4±6 | 66.1±11 | 155.2±6 |  |  | 3 | 13 | 8 |  |  | 3 | 12 |
| Tracy & Enoka, 2006 | Control 1 | Untrained | 0 (0/9) | 74.2±4.9 | 70.3±9.6 | 169±12 | 1RM | Knee Extension |  |  |  |  |  |  |  |
|  | Exercise 1 |  | 0 (0/21) | 71.1±4.2 | 73.6±15 | 167±13 |  |  | 3 | 10 | 1 | 30 | 1063 | 3 | 16 |
| Ünlü et al., 2020 | Control 1 | Untrained | 7 (0/0) | 21.3±2.4 | 67.6±8.8 | 178.4±6.3 | 1RM | Knee Extension |  |  |  |  |  |  |  |
|  | Exercise 1 |  | 6 (0/0) | 20.5±1.9 | 72.8±8.2 | 178.8±8.5 |  |  | 3 | 9 | 1 | 108 | 6388 | 3 | 12 |
|  | Exercise 2 |  | 7 (0/0) | 20.1±1.4 | 70.7±16.7 | 180.1±7.4 |  |  | 3 | 9 | 1 | 83 | 5134 | 3 | 12 |
|  | Exercise 3 |  | 7 (0/0) | 21.6±0.5 | 68.7±8.2 | 176.7±4.9 |  |  | 3 | 7 | 1 | 83 | 5304 | 3 | 12 |
|  | Exercise 4 |  | 7 (0/0) | 22.4±1.5 | 71.3±7.4 | 178.7±1.9 |  |  | 3 | 7 | 1 | 55 | 3218 | 3 | 12 |
|  | Exercise 5 |  | 7 (0/0) | 20.9±2.3 | 72.1±8.9 | 180.6±7.2 |  |  | 3 | 9 | 1 | 68 | 4442 | 3 | 12 |
| Van Roie et al., 2020 | Control 1 | Untrained | 14 (0/0) | 70.5±5.1 | 80.2±10.2 | 177.6±6.5 | 1RM | Leg Press |  |  |  |  |  |  |  |
|  | Exercise 1 |  | 11 (0/0) | 68.2±2.7 | 81.1±9.9 | 170.1±5.4 |  |  | 3 | 11 | 3 |  |  | 3 | 12 |
| Vechin et al., 2015 | Control 1 | Untrained | 0 (0/7) | 66±5 | 69.3±16.1 | 161±1 | 1RM | Leg Press |  |  |  |  |  |  |  |
|  | Exercise 1 |  | 0 (0/8) | 62±3 | 68.7±15.3 | 160±1 |  |  | 4 | 10 | 1 | 75 | 3186 | 2 | 12 |
| Vincent et al., 2002 | Control 1 | Untrained | 0 (0/16) | 71±4.7 | 73.1±13.8 | 169.9±10 | 1RM | Leg Press / Chest Press |  |  |  |  |  |  |  |
|  | Exercise 1 |  | 0 (0/24) | 67.6±6.3 | 77.4±19.3 | 167.2±11.5 |  |  | 1 | 13 | 13 | 50 | 7155 | 3 | 24 |
|  | Exercise 2 |  | 0 (0/22) | 66.6±6.7 | 74.1±14.8 | 167.1±9.7 |  |  | 1 | 8 | 13 | 80 | 11549 | 3 | 24 |
| Walker et al., 2013 | Control 1 | Untrained | 10 (0/0) | 30±4 | 84±14 | 180±6 | 1RM | Leg Press |  |  |  |  |  |  |  |
|  | Exercise 1 |  | 12 (0/0) | 29±5 | 79±11 | 181±6 |  |  | 3 | 11 | 9 | 75 | 5088 | 2 | 20 |
| Wallerstein et al., 2012 | Control 1 | Untrained | 0 (0/13) | 63±4 | 67.2±10.8 | 159.8±6.8 | 1RM | Leg Press / Chest Press |  |  |  |  |  |  |  |
|  | Exercise 1 |  | 0 (0/14) | 63.6±4 | 79.7±17.2 | 163.9±9.8 |  |  | 3 | 9 | 7 | 80 | 3745 | 2 | 16 |
|  | Exercise 2 |  | 0 (0/16) | 64.9±3.9 | 63.9±11.9 | 157.4±7.7 |  |  | 4 | 6 | 7 | 38 | 1575 | 2 | 16 |
| Whitehead et al., 2018 | Control 1 | Untrained | 0 (0/10) | 21.3±1.8 | 80±2.6 | 177.3±9.4 | 1RM | Barbell Squat |  |  |  |  |  |  |  |
|  | Exercise 1 |  | 0 (0/10) |  |  |  |  |  | 3 | 10 | 6 | 75 | 1426 | 2 | 8 |
| Willoughby., 2004 | Control 1 | Untrained | 10 (0/0) | 20.87±2.76 | 78.67±6.23 | 176.45±7.12 | 1RM | Leg Press |  |  |  |  |  |  |  |
|  | Exercise 1 |  | 12 (0/0) |  |  |  |  |  | 3 | 7 | 3 | 88 |  | 3 | 12 |
| Willoughby & Pelsue., 1998 | Control 1 | Untrained | 4 (0/0) | 67.28±2.33 | 87.92±26.87 | 176.93±3.51 | 1RM | Leg Press / Chest Press |  |  |  |  |  |  |  |
|  | Exercise 1 |  | 7 (0/0) | 71.8±6.32 | 86.36±13.42 | 175.39±10.08 |  |  | 3 | 18 | 9 | 63 |  | 3 | 12 |
|  | Exercise 2 |  | 7 (0/0) | 68.7±3.62 | 95.81±18.38 | 176.47±11.83 |  |  | 3 | 9 | 9 | 78 |  | 3 | 12 |
| Willoughby & Rosene., 2001 | Control 1 | Untrained | 6 (0/0) | 20.41±1.73 | 85.49±14.28 | 180.44±3.72 | 1RM | Leg Press |  |  |  |  |  |  |  |
|  | Exercise 1 |  | 8 (0/0) |  |  |  |  |  | 3 | 7 | 3 | 88 |  | 3 | 12 |
| Wilson et al., 1996 | Control 1 | Trained | 14 (0/0) | 24.5±4.9 | 75.9±10.8 | 175±4.2 | 1RM | Barbell Squat / Bench Press |  |  |  |  |  |  |  |
|  | Exercise 1 |  | 14 (0/0) | 22.4±4.7 | 81.1±14.2 | 180±3.5 |  |  | 5 | 8 | 2 |  |  | 2 | 8 |
| Wood et al., 2001 | Control 1 | Untrained | 0 (0/6) | 68±5.4 | 78.1±6.8 | 168.8±3.4 | 1) 5RM; 2) 12RM | 1) Knee Extension; 2) Chest Press |  |  |  |  |  |  |  |
|  | Exercise 1 |  | 0 (0/10) | 69.8±6 | 77.7±6.2 | 168.8±3.5 |  |  | 2 | 10 | 8 | 75 | 11188 | 3 | 12 |
| Yasuda et al., 2011 | Control 1 | Untrained | 10 (0/0) | 23.6±1.6 | 62.2±8.5 | 171±8 | 1RM | Bench Press |  |  |  |  |  |  |  |
|  | Exercise 1 |  | 10 (0/0) | 25.3±2.9 | 63±8.9 | 172±5 |  |  | 3 | 10 | 1 | 75 | 638 | 3 | 6 |
| Ziegler et al., 2019 | Control 1 | Untrained | 0 (0/13) | 66±6.29 | 77.5±16.5 | 175±1 | 1RM | Knee Extension |  |  |  |  |  |  |  |
|  | Exercise 1 |  | 0 (0/12) | 67±5.79 | 77.1±12.2 |  |  |  | 3 | 9 | 7 |  |  | 3 | 52 |

**Note.** Untrained (< 6-moths of resistance training experience) and trained (> 6-months of resistance training experience), sex reported as ‘male (female / male & female combined)’. The absolute intensity (kg lifted) was calculated from the relative intensity prescribed (i.e. 80% 1RM) and the recorded maximal strength reported for each available exercise, absolute intensity was adjusted when mid-testing strength was reported.
